# Dominant HPAIV H5N1 genotypes of Germany 2021/2022 are linked to high virulence in Pekin ducklings

**DOI:** 10.1038/s44298-024-00062-0

**Published:** 2024-11-06

**Authors:** Ronja Piesche, Angele Breithaupt, Anne Pohlmann, Ann Kathrin Ahrens, Martin Beer, Timm Harder, Christian Grund

**Affiliations:** 1 Friedrich- Loeffler- Institute, Institute of Diagnostic Virology, Greifswald, Germany; 2 Friedrich- Loeffler- Institute, Department of Experimental Animal Facilities and Biorisk Management (ATB), Greifswald, Germany

**Keywords:** Virology, Influenza virus

## Abstract

Highly pathogenic avian influenza viruses (HPAIV) of H5 clade 2.3.4.4b pose an ongoing threat worldwide. It remains unclear whether this panzootic situation would favor low virulent phenotypes expected by the ‘avirulence hypothesis’ of viral evolution. Assessing virulence in Pekin ducklings in an intramuscular infection model revealed that the two genotypes that dominated the epidemiological situation in Germany during the period 2021 and 2022 (EU-RL:CH and EU-RL:AB) were of high virulence. In contrast, rare genotypes were of intermediate virulence. The genetic constellation of these reassortants pointed to an important role of the viral polymerase complex (RdRP), particularly the PB1 genome segment, in shaping virulence in ducklings. Occulo-nasal infection of ducklings confirmed the phenotypes for two representative viruses and indicated a more efficient replication for the high virulence strain. These observations would be in line with the ‘virulence-transmission trade-off’ model for describing HPAIV epidemiology in wild birds in Germany.

## Introduction

Highly pathogenic avian influenza virus (HPAIV) causes severe disease and mortality in both wild and domestic poultry species on a global scale and therefore poses a significant threat to conservation efforts and the global agricultural industry^1^. Lately, HPAIV H5 of the so-called Goose/Guangdong lineage (gs/GD) lineage have spread to almost all continents^2,3^, most recently even to the Antarctic region. Mainly detected in avian species, the virus is emerging increasingly also in marine and terrestrial mammals, epitomizing the risk of avian-to-mammalian spillover infection and subsequent adaptation^4,5^. As a member of the genus of alpha-influenza viruses within the family of Orthomyxoviridae, HPAIV is an enveloped virus and contains a single-stranded negative-sense RNA genome. The genome is divided in eight segments coding for a total of up to 17 proteins. Each virion comes with a RNA-dependent RNA polymerase (RdRP)^6^, composed by the association of three polymerase proteins: polymerase basic 2 (PB2), polymerase basic 1 (PB1), and polymerase acidic (PA), with viral genomic RNA encapsidated by the nucleoprotein (NP). Two glycoproteins embedded in viral membrane, the hemagglutinin (HA) and the neuraminidase (NA)^7^ mediate viral attachment to the cellular α-2.3/ 2.6-N-acetyl neuraminic acid receptors with subsequent cell entry and sialidase activity to facilitate release of the new virion from infected cell^8–10^. Today, 16 HA subtypes and 11 NA subtypes have been identified in avian species with wild Anseriformes considered as their natural reservoir. Recently, an H19 subtype has also been suggested in birds^11^. Within the reservoir species, co-infections with different subtypes are considered the sources for versatile reassortment events that are associated with emergence of new genotypes with gene constellations differing from their parental viruses. The majority of avian influenza viruses (AIV), however, induce only a local infection in the respiratory- and gastro-enteric tract and are of low pathogenicity (LPAIV). A molecular marker for low- pathogenic (LP) phenotypes is a monobasic proteolytic cleavage site within the HA-precursor protein (HA_0_). In contrast to all other 14 HA subtypes, in H5 and H7, the proteolytic site can mutate to a polybasic proteolytic cleavage site that enables systemic viral replication, thereby causing severe clinical disease, classified as notifiable highly pathogenic avian influenza (HPAI) in galliform birds^10^. Mutations from LPAIV to HPAIV are mostly associated with galliform infections, although few exceptions have occurred. Such de novo emergence events of HPAI from LPAIV precursor have been documented 39 times since the 1950s^12^, causing outbreaks of different magnitudes but mainly restricted to poultry. The majority of these highly virulent viruses were not able to prevail over a prolonged period of time within a population, due also to consequent restriction measures, such as depopulation, applied to eradicate these viruses. In this respect HPAIV H5 of the gs/GD lineage in 1996 is a notable exception as its successors persist and emerge until today^12–14^. Based on the HA gene, 10 clades (0-9) can be distinguished within the gs/GD lineage^15^, which in turn have established themselves in different regions such as Asia, America, Africa and Europe. Today, the ongoing panzootic is driven by clade 2.3.4.4b viruses. Precursor viruses of clade 2.3.4.4 split, before 2013, into two separated clusters, A (Buan/ Donglim- like) and B (Gochang-like), which had been introduced to Europe in 2014 and 2016 respectively^16,17^. Reassortants of the introduced HPAI H5N8 clade 2.3.4.4b viruses were subsequently the origin of today’s HP H5Nx genotypes that established endemicity in wild birds in Europe^18,19^ and disseminated to the Americas^14^ and Africa. Since 2020, swarms of reassortants were observed with up to five different NA subtypes: Initially, H5N8 and H5N5 strains^16^, sharing high identities with Asian sequences were encountered which were replaced by H5N1 genotypes, that subsequently dominated the epizootic in Germany^19,20^ and Europe. Between October 2021 and beginning of 2022 sixteen different H5N1 genotypes have been discovered in Germany from outbreaks in poultry and cases in wild birds. Within this swarm, certain genotypes seem to dominate the epizootic. For example, genotype DE 21-10 N1.2 (EU-RL:CH) was the most frequently detected one at the end of 2021^21^, but in 2022 was superseded by genotype DE 21-10 N1.5 (EU-RL:AB). For an overview of frequency of different genotypes in Germany and Europe see the associated dashboard^22^. While a lot of efforts have been undertaken to characterize the plethora of emerging genotypes, information on the phenotypic characteristics that might shape the HPAIV H5 pandemic are limited. In particular it is not addressed so far whether dominance of certain genotypes is associated with adaptation and concomitant decrease of virulence as postulated according to the “avirulence hypothesis” originally put forward by Theobald Smith^23^. According to this model, high virulence is in opposition to an expansion of a given pathogen population, as a fast “immobilization” of the host shortens the infection cascade and restricts further spread. On the opposite, in case of subclinical infections the mobile host can be a source of spread of the pathogen for a longer period of time, i.e. lower virulence in the host would help a virus to spread and eventually persist in a population. However, when replication of a pathogen can be assumed to cause some inevitable damage to the host, an increase in the number of pathogens increases both transmission and virulence. This consideration was taken into account when the “virulence-transmission trade-off” hypothesis was introduced^24^. This model, like the “avirulence hypothesis” assumes, that from an evolutionary point of view, it is not beneficial for the pathogen to kill its host before being passed to another host. However, within this model evolution will lead to intermediate virulence, but, depending circumstances, initial virulence may also increase. To study virulence of different HPAIV genotypes in a relevant reservoir species we selected the Pekin duck (*Anas platyrhynchos var. domesticus*). Pekin ducks are the domesticated form of the mallard duck (*Anas platyrhynchos*)^25^, the most abundant wild duck species in the Northern hemisphere, and a well-known natural host of gs/GD HPAIV. Mallards exhibit complex migratory patterns, and frequently switch between partly resident and partly migratory behavior, making them an important species for both dispersal and local maintenance of AIVs^13,26,27^. In general, Anseriformes are highly susceptible to both LPAIV and HPAIV^28^, but clinical disease due to HPAIV infection is often mild compared to commercial galliform poultry species^27,29–31^. In recent years, clinical outcome of infections with clade 2.3.4.4 HPAIVs in ducks have been shown to vary depending on the virus strain, from mild courses of disease induced by viruses of 2014, to severe lethal courses including neurological disorders after 2016^29,32,33^. It remains unclear whether and to what extent virulence in mallard ducks, a globally prevailing wild water bird species, might influence the dominance of certain genotypes during an epizootic. Results obtained during active surveillance efforts, detecting HPAIV shedding in hunted mallards, i.e. birds capable to fly^34^ supported the hypothesis that virulence of circulating HPAIV might have decreased and that lower virulence for mallards might go along with dominance of such pathotypes in wild birds in general. This hypothesis was further promoted by experimental infection studies and field investigations^35–38^ demonstrating subclinical infection of HPAIV H5 infection in Pekin and mallard ducks. In general, such observations might be in line with the “avirulence hypothesis”. To investigate whether such “adaptation” took place during the epizootic 2021/22, we pathotyped in ducklings HPAIV H5N1 genotypes that co-circulated at the end of 2021 (week 52) and compared that to subsequent detection rates of HPAIV H5N1 genotypes in Germany. Pathotyping followed the previously published approach of inoculating HPAIV parenterally into week-old ducklings^32^. However, due to tiny size of veins at this age, a less error- prone parenteral inoculation route, i.e. intramuscular (i.m.) was chosen. This system, referred to as the “intramuscular pathogenicity index” (IMPI) testing has been shown to provide a finer distinction of pathogenicity in semiquantitative terms compared to the more categorical yes/no results that are usually obtained with chickens and the intravenous inoculation system (IVPI). Based on the IMPI results our studies point out that higher assertiveness of individual genotypes in the field went hand in hand with a significant higher virulence in Pekin ducklings. These data on altered pathogenicity in the Pekin duckling model shed light on the driving forces of the current panzootic, with dominance of the most virulent variants thereby superseding HPAIV strains of lower virulence.

## Methods

### Viruses

Details of the viruses used in this study are given in Table 1. Basis for selecting the viruses was the database featured in the public dashboard “HPAIV genotypes in Germany” ^22^, which holds sequence data from the National Reference Laboratory for Avian Influenza in Germany. Retrospectively, these data indicate dominance of certain genotypes. For the phenotypic characterization we focused on genotypes present in the 52nd calendar week of December 2021, a week when 7 different genotypes were detected in Germany. It was possible to obtain isolates of 6 genotypes of week 52 (#3, #4, #6, #7, #8, #9), while isolation of a seventh genotype detectable in week 52, namely DE 21-12 N1.6, failed. In addition, derived from the two sites genotype #8 was exclusively detected, genotype #5 and two further isolates of genotype #4 (#4:1 and #4:2) were included. The three isolates of genotype #4 take its massive and long-term presence throughout 2022 into consideration and represent isolates from three timepoints throughout 2022, i.e. February (#4:1), June (#4:2), and July (#4:3). Viruses were isolated from swab samples of deceased birds sent to the national reference laboratory for avian influenza at the Friedrich-Loeffler-Institute (FLI). For virus isolation, samples were first enriched in cell culture (Immortalized chicken hepatocytes ([LMH cell line, ATCC (CRL-2117), 1997]) by infecting them with 500 µl of 1:10 prediluted and filtrated (Millex® Syringe- driven Filter Unit −0.22 µm) (SigmaAldrich) viruses and then incubating them for at least 96 h at 37 ⪚C and 5% CO_2_ concentration, in Dulbecco’s Modified Eagle Medium (DMEM) (Thermo Fisher Scientific), supplemented with 0.86 g/L NaHCO3 (Carl Roth) and 10% Fetal Calf Serum (FCS). At the end of the period, the cells were visually checked for a cytopathic effect (CPE) such as formation of virus- associated circular plaques or general cell necrosis. Cell culture supernatants were examined for viral hemagglutination capacity using a hemagglutination assay (HA)^39^. HA assay positive supernatants were then added in a quantity of 200 µl for propagation in the allantoic cavity of 10-day-old embryonated eggs from chicken specific pathogens free (SPF). Inoculated eggs were then incubated at 37.5 °C for at least 96 h and death of the eggs was monitored every 12 h by candling. Amnio-allantoic fluids (AAF) were then harvested from eggs harboring dead embryos and centrifuged for 10 min at 1500 revolution per minute (rpm) before storage of the supernatants at -70 °C until further use.

**Table 1:** Characteristics of German HPAIV-H5N1 clade 2.3.4.4b genotypes investigated in this study

Genotype			Accession number^a^	Virus isolate	TCID_50_/dose^b^	IMPI
code	Germany-RL^c^	EU-RL^d^				
#1	DE 20-10 N8		accession 5146288	*A/chicken/Germany-NW/AI 3705/2021 (H5N8)*	1.40 × 10^6^	2.52
#2	DE 02-21 N8		accession 4804850	*A/seal/Germany-SH/AI 5373/2021 (H5N8)*	2.86 × 10^3^	2.69
#3	DE 21-10 N1.2	CH	accession 18006762	*A/European herring gull/Germany-MV/AI 1411/2022 (H5N1)*	5.02 × 10^7^	2.92
#4:1	DE 21-10 N1.5	AB	accession 17693298	*A/European herring gull/Germany-SH/AI 1196/2022 (H5N1)*	9.90 × 10^6^	3.00
#4:2	DE 21-10 N1.5	AB	accession 17693301	*A/brent goose/Germany-SH/AI 2407/2022 (H5N1)*	5.48 × 10^7^	2.95
#4:3	DE 21-10 N1.5	AB	accession 16096050	*A/chicken/Germany-NI/AI 4286/2022 (H5N1)*	2.86 × 10^7^	2.99
#5	DE 21-11 N1.1		accession 17693297	*A/European herring gull/Germany-SH/AI 7088/2021 (H5N1)*	2.40 × 10^7^	1.65
#6	DE 21-12 N1.2		accession 18006937	*A/chicken/Germany-MV/AI 1026/2022 (H5N1)*	2.00 × 10^6^	1.52
#7	DE 21-12 N1.3		accession 10261376	*A/pigeon/Germany-NW/AI 951/2022 (H5N1)*	7.47 × 10^6^	2.96
#8	DE 21-12 N1.4		accession 5098132	*A/red knot/Germany-SH/AI 616/2022 (H5N1)*	8.28 × 10^5^	1.22
# 9	DE 21-12 N1.5		accession 18006938	*A/black-headed gull/Germany-HH/AI 1073/2022 (H5N1)*	2.40 × 10^7^	1.26

^a^Accession number according to the gisaid.org EpiFlu^TM^ platform.

^b^TCID50/dose per 0.1 ml inoculum.

^c^genotype designation by the national reference laboratory for avian influenza in Germany (RL); code for the country and federal state code is given according to ISO 3166.

^d^genotype designation by the EU reference Laboratory (RL) for avian influenza (provided where available).

Each isolate used for animal experiments was characterized by full genome sequencing and subsequent genotyping as described in ref.^40^.

### Virus titration

Immortalized chicken hepatocytes [LMH cell line, ATCC (CRL-2117), 1997] were cultivated at 37 °C and 5% CO_2_ concentration, in Dulbecco’s Modified Eagle Medium (DMEM) (Thermo Fisher Scientific), supplemented with 0,86 g/L NaHCO_3_ (Carl Roth) and 10% Fetal Calf Serum (FCS). For virus titration, LMH cells were incubated in 96 well plates without FCS but supplemented with TPCK Trypsin (2 µg/ml). For determination of tissue culture infectious dose 50 (TCID_50_), four serial tenfold dilution rows were prepared of each virus stock and each dilution row was added in triplicates on 96 well plates with 50 µl per well. Development of cytopathic effects (CPE) was visually scored by microscopic investigation. After 72 h of incubation formation of CPE had fully developed in form of virus- associated circular plaques or general cell necrosis. TCID_50_ was then calculated according to the Reed & Muench formula^41^.

### Animal experiments

All animal experiments were in accordance with relevant guidelines and regulations of the state office for agriculture, food safety and fishery in Western pomerania (LALLF) and have been have been approved by an independent ethics commission and granted permission by the state office for agriculture, food safety and fishery in Western pomerania (LALLF) under the registration number LALLF 7221.3-2-009/19. Pathotyping of HPAIV was done by intramuscular (i.m.) inoculation of one-week old Pekin ducklings (*Anas platyrhynchos var. domesticus*) as described^32^. Shortly, one day old ducklings were obtained from a commercial hatchery. The parental flocks are routinely screened serologically to exclude AIV infections at a prevalence of 5% (90% confidence). In addition, 10 ducklings from the same batch were euthanized for testing and all yielded seronegative results as well as negative PCRs from swab samples. After an acclimatization phase of one week, all animals were healthy and randomly separated by allocating individual picked birds alternating to nine different groups with ten birds each. Each bird was inoculated with 0.1 ml of a 1/10 diluted AAF from infected eggs into the caudal femoral muscles. The group size, virus quantity and dilution scheme were based on IVPI standards^39^. Birds of each group were kept on the floor of separated rooms and were allowed to move freely within an enclosure of 6.46 m^2^. They had access to food, drinking water ad libitum with an additional water tub for bathing and dry places with infrared lamps to warm up.

Subsequently, birds were clinically monitored for 10 days which included daily veterinary check- ups and supervision by animal care staff supported by a 24 h daily video surveillance program, accessible to all persons involved in the experiment. The scoring scheme provided categorization of animals as healthy [0], sick [1] severely sick [2] or dead [3], a wording adopted from the IVPI definition format. “Sick” reflects a general body condition, where animals appear weak, less enthusiastic to follow the cage mates. At this stage animals respond to external impulses and are willing to eat and drink. “Severely sick” encompasses a body condition where vital sings as described above are missing and/or sings of central nervous disorders are evident, and corresponds to the defined human endpoints of disease. Animals reaching these endpoints in the context of the experiment were euthanized by stunning by means of a blunt blow to the head and subsequent bleeding by opening the carotid arteries on both sides, with final dislocation of the cervical vertebrae and severing of the spinal cord at the neck (Annex IV of Directive 2010/63/EU; No. 2 c). These animals were scored as dead from the following day on. Based on such scoring, a clinical intramuscular pathogenicity index (IMPI) was calculated according to the standard protocol for the intravenous pathogenicity index (IVPI) of avian influenza viruses^39^.

For occulo-nasal (o.n.) infection, one-week old Pekin ducklings, raised as described for the IMPI, per groups of ten ducklings, were inoculated with 0.1 ml of the same virus stock from respective genotypes, again 1/10 diluted as for the IMPI (Supplementary Table 1). Per animal 1 drop of virus suspension was conjunctivelly applied while the rest of inoculum was applied through the nares onto the choanal epithelia. From one day post infection (dpi) on, two additional ducklings were housed together with the ten inoculated animals to serve as sentinels for virus transmission. The animals were kept under the same conditions as the within the IMPI trials, i.e. they had unrestricted access to bathing water and ad libitum feeding and drinking supply. Clinical supervision by veterinarians included daily health checks as well as supervision by trained animal care staff for the whole duration of the experiment. Clinical score values per animal for an observation time of 10 days were used to calculate a group clinical score according to the previous IMPI, similar to calculation of the IVPI used in chickens. In addition, choanal and cloacal swab samples were taken daily for the first seven dpi, added into tubes with 1 ml cell culture medium supplemented with Enrofloxacin (Baytril®; 20 µg/ml) and stored at −70 °C until further analysis. Due to the extended survival of ducklings during the IMPI, the monitoring period of the o.n. experiment was prolonged for up to 21 days after inoculation.

A postmortem examination with tissue sampling was performed on acutely diseased animals for o.n. inoculated animals or from surviving animals on the last day of the experiment i.e. on10 dpi for the IMPI groups and 21 dpi for the o.n. experiment. Samples of brain, lung, duodenum/pancreas, kidney, liver and heart were taken, added into tubes containing 1 ml of cell culture medium with Enrofloxacin (Baytril®; 20 µg/ml) and stored at −70 °C until further analysis. Blood was collected from all remaining ducklings at the final day of the experiment.

### Pathology

Histological evaluation was performed on three ducks each, that were either moribund and euthanized, i.e. o.n. inoculated animals infected with genotype #4:3 (3 dpi) or genotype #8 (5-6 dpi), respectively (Fig. 3; Supplementary Figures 2–4). In addition, for genotype #8 organ samples were taken from animals that survived until the end of the observation time, i.e. 21 dpi for o.n. inoculated and 10 dpi for i.m. inoculated animals. Samples from nasal conchae, lung, heart, kidney, brain, and spinal cord (thoracal, lumbar) were fixed in 10% neutral buffered formalin. Tissues were paraffin-embedded and 2–4 μm-thick sections were stained with hematoxylin and eosin (H&E). Immunohistochemistry (IHC) was performed for viral antigen detection using a primary antibody against the M protein of IAV (ATCC clone HB-64) as described in ref. ^42^. Slides were scanned using a Hamamatsu S60 scanner and analyzed using NDPview.2 plus software (Version 2.8.24, Hamamatsu Photonics, K.K. Japan). HE stained sections of all tissues were evaluated and described. Following IHC, the distribution of IAV matrix protein was recorded on an ordinal scoring scale with scores 0 = no antigen, 1 = oligofocal, affected cells/tissue < 5% or up to 3 foci per tissue; 2 = multifocal, 6%–40% affected; 3 = coalescing, 41%–80% affected; 4 = diffuse, > 80% affected. The target cells were identified based on the morphology. Evaluation and interpretation were performed by a board-certified pathologist (DiplECVP) in a blinded fashion.

### Real- time RT-qPCR

For further processing swab samples of the o.n. inoculated birds (Supplementary Table 2) were thawed and vortexed in a Thermomixer (Thermomixer comfort® Eppendorf) at room temperature for 5 min. Organ samples were directly homogenized in tubes containing a steel bead by a tissue lyser (TissueLyser II Qiagen, Hilden, Germany) while oscillating for 3 minutes at 30 Hz. RNA was extracted from 100 µl of centrifuged supernatant from swab- and organ samples by the Macherey-Nagel NucleoMag® VET-Kit according to the Manufacturer´s instructions using the Biosprint 96 extraction robot (Qiagen, Hilden, Germany) and eluted in 100 µL elution buffer.

Subsequently, preparations were tested by real-time RT-PCR (RT-qPCR) for the presence of influenza A virus specific matrix gene RNA (M1.4) using the AgPath ID One-Step RT-PCR Kit (Ambion-Applied Biosystems) as described in ref. ^43^. PCR tests were carried out in a CFX96™Real-Time-System C1000™ thermal cycler (BioRad, Munich, Germany). Estimation of virus genome equivalents per milliliter (VE/mL) in each sample is based on correlation of the individual Cq values to an intra-assay calibration curve of a defined HPAIV H5N1 virus stock. The value is derived from standard titration experiments of infectivity of a virus suspension in cell culture medium. The log_10_ dilutions are tested in parallel by RT-qPCR. The resulting correlation curve of TCID_50_ and Cq values are used to derive the VE value.

### Serology

Blood samples were collected from all ducklings reaching the respective end of the experiment alive. Blood was drawn into a tube containing heparin, plasma separated by centrifugation and then subjected to heat inactivation at 56 °C for 30 minutes. Plasma samples of the ducklings were screened by ELISA for Influenza A-specific antibodies using two different commercial test kits as indicated for the specific sera (IDEXX AI MultiS- Screen Ab Test or ID screen® Influenza A Antibody Competition Multi- species ELISA). The cut-off for the IDEXX ELISA used was <0.5 S/N (Signal to noise ratio) for positive sera, while sera with a value of >0.5 were assessed as negative. Positive results were set for the ID- vet ELISA at a cut-off of <45% S/N (%) inhibition, while results >50% S/N (%) inhibition were considered negative. The range in between was to be considered questionable. Subsequently, hemagglutination inhibition (HI) assays were performed on ELISA- positive samples using homologous and heterologous antigens AIV, respectively, according to standard procedures^39^. By tilting the plates, the agglutination of the plate could be assessed, whereby the HI titre of a sample corresponds to the highest serum dilution, which causes a complete inhibition of 4 HAU (Hemagglutination Units) of the antigen^39^. For HI assays, duck samples were pretreated with KJO_4_-solution to remove non-specific inhibitors.

### Statistics

Statistical analyses on significance were performed as indicated where appropriate. Differences in IMPI clinical scores between all groups was statistically evaluated using unpaired two-tailed Mann- Whitney- U tests. The data on oropharyngeal and cloacal excretion of the two occulo-nasally infected groups #4:3 and #8 were statistically evaluated using Kruskal-Wallis One-Way- ANOVA on ranks, Tukeys tests and following unpaired two-tailed Mann-Whitney-U tests when appropriate. *P*- values as well as associated effect sizes are given in the text or in the supplemental material (Supplementary Figure 1; Supplementary Table 6; Supplementary Table 6.1). Statistical analysis was applied with help of the statistical analysis tool GraphPad Prism software version 8.0.1 (GraphPad Software, San Diego, CA, USA) as well as SigmaPlot™ version 11 (SigmaPlot Software, Grafiti LCC, Palo Alto, CA, USA).

### Sequencing

Full-genome sequencing for genotype confirmation of all isolates from passaged viruses on LMH cells as well as AIV-positive organ samples was done as described^40^: after initial RNA extraction via the Qiagen Mini Viral Kit (Qiagen, Germany) and subsequent AIV-End-RT-PCR applying Superscript III One-Step, sequencing was executed by a nanopore-based amplification method. After purification of the PCR products with AMPure XP Magnetic Beads (Beckman-Coulter, USA), full-genome sequencing utilized the Mk1C MinION platform (Oxford Nanopore Technologies, ONT, UK) in combination with the Rapid Barcoding Kit (SQK-RBK004, ONT) for sample multiplexing. Sequencing was directed according to the manufacturer’s instructions with a R9.4.1 flow cell.

### Genotyping

The designation of genotypes was applied on a national level, based on Germany as country of virus isolates’ origin, the year and month of the first detection of a particular genotype within a typed sample, and finally the NA subtype as described in refs. ^19,40,44^. Matching nomenclature proposed at the European level applied by the European Reference Laboratory (EU-RL) was used where appropriate. Consecutive hash numbers enable an easier identification of the genotypes used here (Table 1).

### Phylogenetic analysis

Based on the classification of different genotypes, closely related sequences were selected by blasting the non-redundant nucleotide database at the NCBI (National Center for Biotechnology Information; NCBI; Bethesda, MD, USA) and the EpiFlu™ platform of the Global Initiative on Sharing All Influenza Dara (GISAID) (https://blast.ncbi.nlm.nih.gov/; https://platform.epicov.org/), entering accession numbers or FASTA sequences of genotypes used in our trials as well as applying search pattern filters on the Influenza Type A, H1-10, N 1-10, animal hosts, location between Europe, Asia and America as well as on clade 2.3.4.4 viruses. Additional filters were applied on required segments such as PB2 and PB1. For further information on retrieved sequences please see Supplementary Table 5. Sequences got downloaded and further analyzed using MEGA X: Molecular Evolutionary Genetics Analysis across computing platforms software version 10.2.4 (Kumar, Stecher, Li, Knyaz, and Tamura 2018)^45^. The alignment was fed with additional, related sequences^19,46^. Sequences were finally aligned using the MUSCLE algorithm contained in MEGA X: Molecular Evolutionary Genetics Analysis across computing platforms software version 10.2.4 (Kumar, Stecher, Li, Knyaz, and Tamura 2018) applying the following parameters: maximum 16 iterations, cluster method UPGMA. Phylogenetic analyses were run on nucleotide sequences using the Phylogeny tool in MEGA X: Molecular Evolutionary Genetics Analysis across computing platforms software version 10.2.4 (Kumar, Stecher, Li, Knyaz, and Tamura 2018). More precisely, Maximum likelihood (ML) trees were computed utilizing a General Time Reversible Model (GTR) + gamma (G) with rapid bootstrapping and search for the best scoring ML tree supported with 1000 bootstrap replicates. Correspondingly protein sequences were translated and phylogenetic trees based on the ML method with 1000 bootstrap values were created again using the MEGA X Phylogeny tool. The resulting phylogenetic nucleotide and translated protein trees were color- coded according to their genotype- distribution using a vector graphics editor (CorelDRAW 2017) and representative virus isolates referencing certain genotypes were marked by a dot.

## Results

### HPAIV H5N1 genotypes of clade 2.3.4.4b differ in virulence for ducklings

**Fig. 1:**
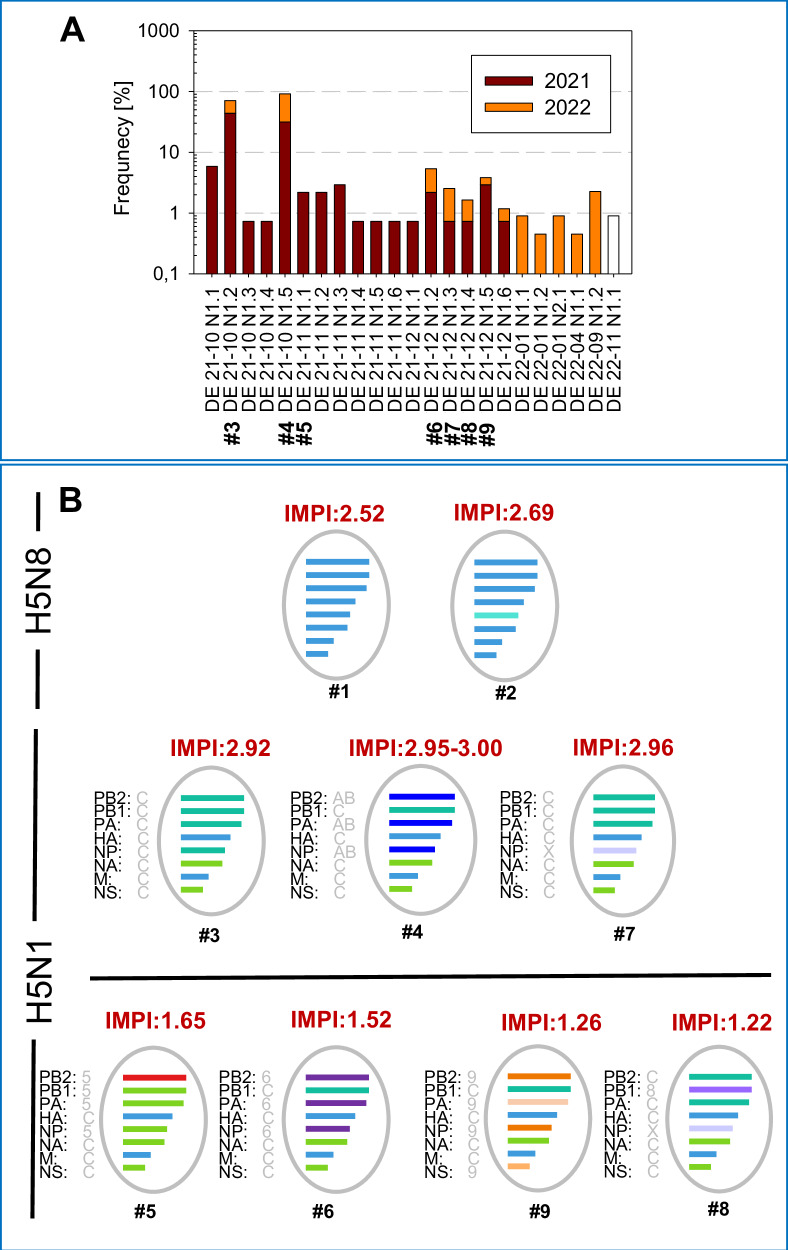
Geno- and phenotype of co-circulating HPAIV H5 in Germany during 2021-2022. HPAIV H5N1 clade 2.3.4.4b genotypes co-circulating in December 2021 (week 52) in Germany were characterized with respect to their duckling virulence by IMPI and compared to 2 HPAIV H5N8 genotypes, the subtype dominating HP epidemics until April 2021. **A** Detection frequency of genotypes prevalent in Germany during 2021 and 2022 are given and tested viruses are indicated (#). **B** Depiction of genetic composition of the tested genotypes, based on analyses described previously^21^. The genotypes are shown with their IMPI score, that is given above the virus cartoons. The horizontal black line separates genotypes with high (upper panel) from those with intermediate virulence (below). The origin/relation of the segments to other genotypes is given by letters besides the segment names with designation based on the EU-RL nomenclature).

Phylogenetic analyses on current genotypes in combination with the epidemiological information reveal, that despite the diversity of simultaneously circulating genotypes, the current epizootic as a whole was dominated by single, individual genotype (Fig. 1A)^44^. For the phenotypical characterization of co-circulating HPAIV H5N1 clade 2.3.4.4b genotypes we focused on the last week of December 2021 (week 52), a time period when seven different genotypes of HPAIV H5N1 were present in Germany and compared the virulence to frequency of detection in subsequent year 2022. Representative virus isolates of six out of these seven genotypes present in December could be obtained from samples obtained in 2021-2022 by cultivation on LMH-cells. No isolate could be obtained from one genotype, namely DE 21-12 N1.6. Conformity with original samples was demonstrated by full- genome sequencing, verifying their distinct genotypes (Fig. 1B, genotypes #3 - 9). For comparison, one H5N1 genotype first detected in November (#5) as well as two older H5N8 clade 2.3.4.4b viruses representing two further distinct genotypes (#1, #2) were included in the pathotyping approach. Both H5N8 genotypes circulated in Germany until April 2021, but have since than been replaced by H5N1^20^. Genotype #5 as well as two more viruses of genotype #4 (#4:1 and #4:2) were included as they are derived from the same sites as genotype #8 (January 2022), representing viruses from November 2021 (#5).

In seven-day old pekin ducklings, both H5N8 viruses of genotypes #1 and #2 induced 100% mortality within three to six dpi, resulting in an IMPI score of 2.52 and 2.69, respectively (Fig. 1B; graphical representation and data for individual animals are listed in Supplementary Figure 1; Supplementary Table 10). However, their pathogenicity was exceeded by genotype #3 (IMPI 2.92), a genotype emerged in October of 2021 and dominating the German HPAIV epidemic with a total of 60 (43.8%) of the sequenced HPAIV H5N1 viruses up to the end of 2021 (Supplementary Table 1). In frequency of detection genotype #4 was the second highest disseminated genotype, detected in 43 (31.4%) of HPAIV H5N1 viruses sequenced in 2021 (Fig. 1A; Supplementary Table 1). Compared to both precursor H5N8 viruses, IMPI scores of three genotype #4 virus isolates were significantly higher (*p* < 0.005, r_U_ > 0.6) with scores of 3.00 (#4:1), 2.95 (#4:2) and 2.99 (#4:3), respectively, see Supplementary Figure 1; Supplementary Table 6 for statistical analysis) and inducing mortality within 2 dpi. These later three HPAI H5N1 2.3.4.4b viruses of the same genotype #4 have been recovered in February 2022 (#4:1), April 2022 (#4:2) and July 2022 (#4:3) and were included to evaluate consistency of IMPI scores (Table 1). The results indicate, that the virulence of this genotype was stable and even slightly higher than genotype #3: Whereas for genotype #3, 7 of the 10 inoculated ducklings were alive on one dpi, two genotype #4 isolates induced death in all (#4:1) and nine (#4:3) ducklings within one day, resulting in significant higher IMPI scores (#4:1 *p* = 0.0031, r_U_ = 0.7 and #4:3 *p* = 0.0174, r_U_ = 0.6). With regards to frequency of detection the order of the genotypes changed in the following year 2022, with 133 of 222 sequenced viruses belonging to genotype #4 (59.9%), in contrast to only 60 (27.0%) of the sequenced viruses from 2022 belonging to genotype #3. Nevertheless, during 2021 and 2022 genotypes #3 and #4, highly virulent for ducklings by IMPI, together made up 75 and almost 90% of all sequenced HPAIV in Germany.

Of almost equally high duck virulence was an isolate representing genotype #7, inducing 100% mortality within 24 h (IMPI of 2.96). This particular virus was recovered from a wood pigeon (*Columba palumbus*) that was euthanized during an outbreak in a wild bird sanctuary in January 2022^47^. However, this genotype remained a minor population detected generally, with one detection up to the end of 2021 and four in 2022 in poultry and wild birds (see Supplementary Table 1), constituting 1.8% of sequenced viruses in the period 2021 and 2022.

**Fig. 2:**
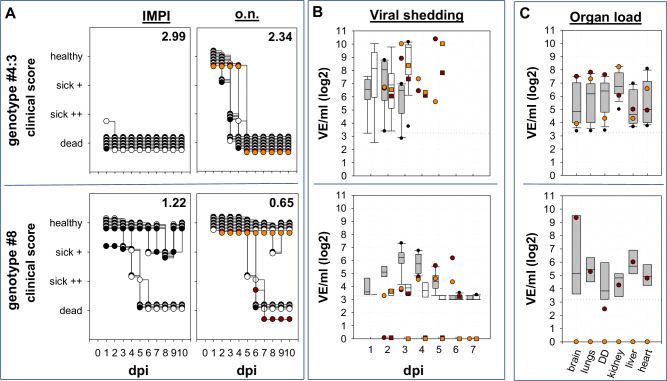
Clinical course and viral shedding after o.n. HPAIV H5N1 infection with genotypes of high and moderate duckling virulence. Depicted are individual daily clinical scores after i.m (IMPI) and o.n. inoculation with genotypes #4:3 and #8 respectively. Two different sentinel animals in each of the o.n. inoculated groups are individually marked by orange and red dots, respectively. **A** Clinical disease was observed up to 10 dpi and to better compare both types of infection clinical scores (numbering in the top right corner of each graph) were determined after ten days. In the stacked format of the graph white and black dots are used only to contrast different individual inoculated ducks. **B** Viral shedding was tested by pharyngeal - (grey boxes) and cloacal swabs (transparent boxes) by RT-qPCR and results are standardized to virus equivalents (VE/ml) as described in material and methods. Viral shedding from two sentinel animals per group is given individually by orange and red dots for pharyngeal shedding as well as squares for cloacal shedding. Please see Supplementary Table 6 for further information on statistical analysis. The dotted horizontal line represents the cut-off. **C** Likewise, organ samples from o.n. inoculated ducklings of both groups were investigated, taken either at the time of euthanasia or at the end of the observation time. Again, sentinel animals are indicated by an orange and a red dot.

The remaining three genotypes that co-circulated in December 2021 (#6, #8 and #9) remained rare detections, representing 10 (2.8%), 3 (0.8%) and 6 (1.7%) of the total number of viruses sequenced in Germany for the period up to the end of 2022. Obtained virus isolates from 2022 from genotypes #6, #8 and #9 were of moderate duck virulence with significant lower IMPI scores compared to dominate genotype #3 (#8 *p* = <0.0001, r_U_ = 1; #9 *p* = <0.0001, r_U_ = 1) and genotype #4 (*p* = <0.005, r_U_ = 1) (Supplementary Figure 1; Supplementary Table 6): besides birds that succumbed to infection two to six days after inoculation, single birds showed only moderate or no clinical signs at all up to the end of the observation period, resulting in IMPI scores between 1.22 and 1.52. For example, in the group with the lowest IMPI, i.e. the genotype #8 virus, only four out of nine birds died or had to be euthanized because of severe signs of disease dominated by disorders of the central nervous system (CNS), while five birds had recovered from slight apathy at day 7 to 9 after infection (dpi) (Fig. 2; Supplementary Fig. 1).

### Outcome of infection with moderate HPAIV H5N1 genotypes

**Fig. 3:**
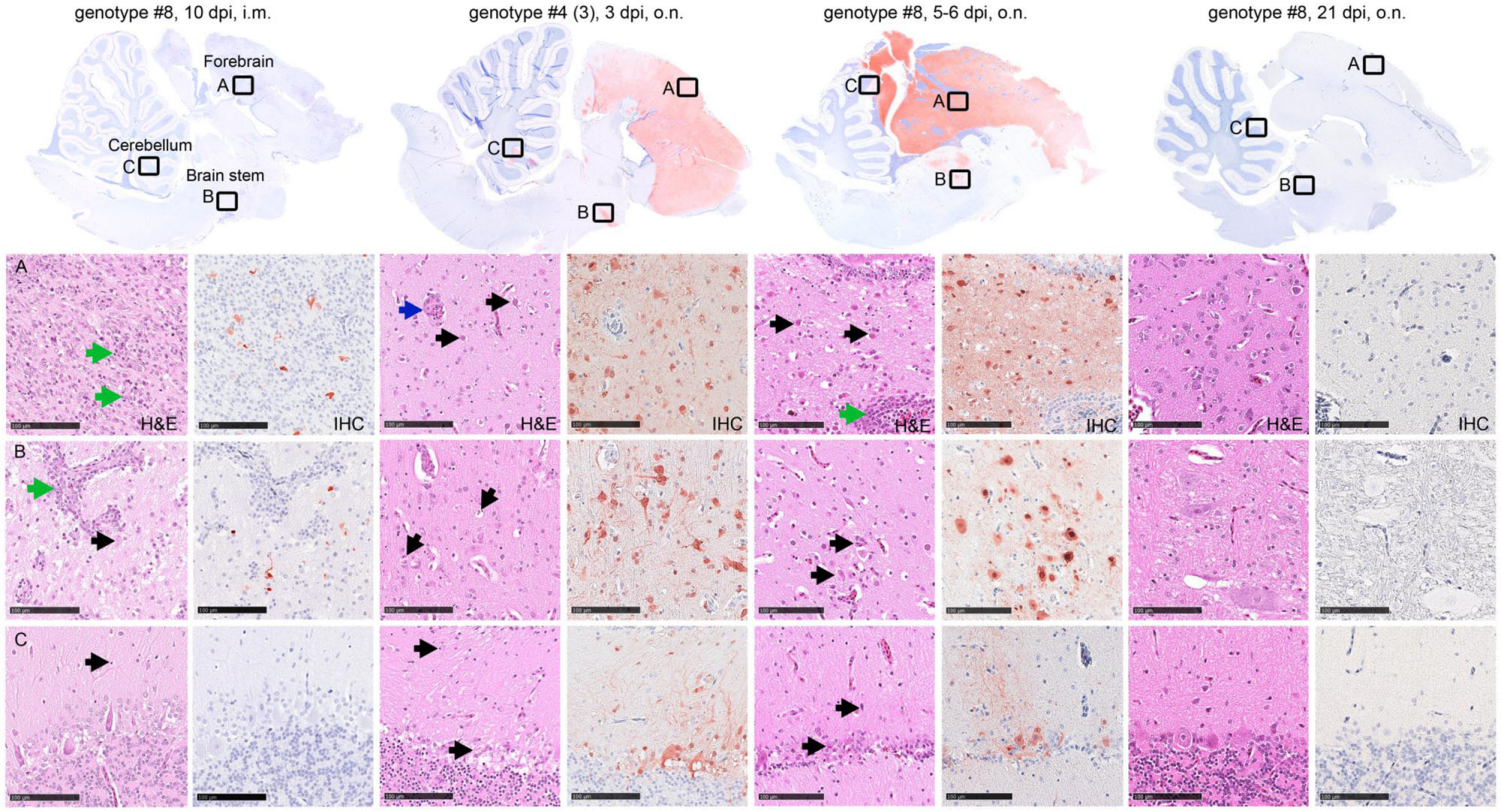
Pathological findings and Influenza A virus antigen detection in the brain. Shown are HE staining and antigen detection in different brain areals of ducks inoculated with genotype #8 (10 dpi, i.m.), genotype #4:3 (3 dpi, o.n.) or genotype #8 (5-6 dpi, o.n.; 21 dpi, o.n.). Necrotizing meningoencephalitis, with intralesional virus antigen detection, was present following i.m. inoculation with genotype 8 and in moribund/deceased birds after o.n. inoculation with genotype #8 or genotype #4 (3). The (**A**) cerebrum and (**B**) brain stem, were most affected, the (**C**) cerebellum was significantly less affected. Blue arrows indicate activation of blood vessels, black arrow point to necrotic neurons and glial cells, green arrows show immune cell infiltrates, perivascular and disseminated in the neuropil. Dpi days post infection,i.m. intramuscularly, o.n. occulo-nasally, hematoxylin-eosin stain, H&E, immunohistochemistry IHC. Bar 100 µm.

All ducklings that survived until the end of the observation period of 10 days had seroconverted by ELISA (Supplementary Table 4) proving they all had become infected. In addition, organ samples taken at the end of the experiment from ducklings of the genotype #8-infected group- showing the lowest IMPI value - tested virus positive: Viral RNA was detected in the brain of all 5 ducklings on 10 dpi (9.1 × 10^3^– 2.8 × 10^5^ VE/mL). However, attempts to isolate infectious virus from late brain samples of genotype #8-infected ducklings failed. To a lesser extent, residual viral RNA was detected in the heart (4 of 5), the lungs (3 of 5) and the duodenum (2 of 5) of genotype #8 infected ducklings. Likewise, ducklings infected with genotypes #6 or #9, survived the ten-day observation period (*n* = 6 of each group), showing no (#9: *n* = 4) or only mild signs of depression (#6: *n* = 4) but harboring viral RNA in the brain (#9: *n* = 4; #6: *n* = 6) (Supplementary Table 3). The detailed histological evaluation of 3 ducks, euthanized 10 days after i.m. inoculation with genotype #8, revealed that virus antigen was still detectable multifocally in neurons and oligo focally in glia cells of the CNS in 2 out of 3 animals. Although clinical signs were lacking at 10 dpi, all ducks showed a moderate to severe, subacute, necrotizing meningoencephalitis predominantly in the cerebrum and brainstem, but hardly in the cerebellum (Fig. 3). No virus antigen was detected in the remaining examined tissues, however, 2 out of 3 ducks exhibited mild, chronic interstitial pneumonia, partially with cellular debris in the air sac and one animal showed minimal, chronic myocarditis. No lesions were recorded in the kidneys (data not shown). For details on virus antigen detection, see supplementary table (Supplementary Table 9).

These results revealed striking differences in duckling pathogenicity between different genotypes of HPAIV H5N1 of clade 2.3.4.4b, indicating that epidemiologically dominant genotypes were more virulent for ducks. Interestingly, ducklings surviving infection up to day 10 dpi with genotypes with moderate virulence, although clinically inconspicuous, showed meningoencephalitis and virus antigen-positive neurons and glial cells in the brain, confirming a general neurotropism of the different HPAIV H5 genotypes.

### Genotype-related distinct virulence for ducklings is also verified by the occulo-nasal inoculation route

To investigate the influence of the inoculation route on duckling virulence, one isolate of high (genotype #4:3; IMPI: 2.99) and moderate duckling virulence (genotype #8; IMPI: 1.22) were tested in ducklings applying occulo-nasally inoculation route, mimicking a more natural route of infection compared to parenteral inoculation. Like after the i.m. inoculation, the virulence of the two genotypes differed dramatically in ducklings: Whereas genotype #4 induced mortality in all ten inoculated and both sentinel ducks, six out of ten ducklings inoculated with genotype #8 and one sentinel animal survived infection and showed no clinical signs after day 8 p.i. All seven surviving ducklings from genotype #8 group were subsequently observed for 21 dpi and remained clinically unremarkable and seroconverted (Supplementary Table 4; 4.1). However, compared to the i.m. inoculation, the course of disease after o.n. inoculation was delayed resulting in lower clinical scores (CS) for both genotypes: CS of 2.34 vs. IMPI of 2.99 (*p* < 0.0001, rU=1) for genotype #4 and CS of 0.65 vs. IMPI of 1.22 (*p* = 0.0383, rU=0.6) for genotype #8 (Fig. 2A; Supplementary Table 6). Concerning viral replication, the higher virulence of genotype #4 virus was associated with a faster increase of viral shedding in o.n. inoculated ducklings (Fig. 2B). Starting at 1 dpi already 9 out of 10 pharyngeal (1.75 × 10^3^ to 3.46 × 10^7^ VE/mL).The highest viral load was detected in pharyngeal swabs from both sentinal animals with 1.02 × 10^10^ and 2.38 × 10^10^ VE/mL on 2 and 5 dpi respectively. Pharyngeal viral shedding remained on that high level up to the death of the animals. In comparison, in the group of genotype #8 inoculated ducklings, 8 out of 10 pharyngeal swabs were virus positive on 1 dpi, but at a considerable lower level (5.13 × 10^3^ to 2.87 × 10^5^ VE/mL) than in the genotype #4 group. During the following days, pharyngeal viral shedding increased, with 10 out of 10 swab samples testing positive on 3 and 4 dpi, with the peak of viral shedding observed on day 4 (5.49 × 10^3^–2.15 × 10^7^ VE/mL) remaining up to 28 times lower (Supplementary Table 2) compared to genotype #4 inoculated ducklings. Remarkably, cloacal shedding remained at very low levels in this group: within the first 7 dpi, only single cloacal swabs tested positive at low loads with significant differences in shedding between groups on 3 dpi (*p* = 0.004, r_U_ = 0.94). Nevertheless, both sentinel animals of the genotype #8 group tested positive in pharyngeal swab samples from day 3 or 6 dpi, demonstrating successful virus transmission. Blood samples taken on the last day of the observation period, i.e., 21 dpi, confirmed seroconversion in all six surving inoculated and one sentinel animal of the genotype #8 group by ELISA (Supplementary Table 4) and also by HI. Testing with different antigens, sera showed preferential binding to homologous antigen with HI-titers between 6-8 [log_2_], compared to HI-titers between 4 and 6 [log_2_] to heterologous antigens from previous years (2014 and 2016) (Supplementary Table 4.1).By occulo-nasal inoculation mimicking a more natural route of infection the dualism of highly and moderately virulent genotypes of HPAIV H5N1 clade 2.3.4.4b was confirmed.

### Clinical manifestations after occulo-nasal inoculation reveal dominance of neurological signs

Diseased animals inoculated with highly virulent genotype #4 had to be euthanized on day 3 because of progredient CNS disorders. Virological examination of these birds revealed presence of influenza A virus RNA in almost all tested organs (Supplementary Table 2), with the highest viral load detected in the kidneys, with virus loads ranging from 2.09 × 10^3^ VE/mL to 1.86 × 10^6^ VE/mL. Histopathological analysis of organs of three acutely deceased birds revealed an acute, necrotizing meningoencephalomyelitis in all birds with intralesional virus antigen detection particularly in the forebrain (score 4) and brain stem (score 1-3), and to a lesser extent in the cerebellum (score 0-3) (Fig. 3) and spinal cord with dorsal root ganglia (score 1-3) (Supplementary Figure 2). Target cells comprised neurons, glia cells, ventricle and central canal epithelium. Furthermore, a severe, acute necrotizing rhinitis, sinusitis, air sacculitis and pneumonia was recorded, with intralesional virus antigen (score 2-3) detected in the olfactory and respiratory epithelium of the conchae, in the respiratory sinus and air sac epithelium as well as in the pulmonary bronchial, parabronchial and capillary epithelium (Supplementary Figure 3; 3.1).

Likewise, organ samples from four ducklings that died in the moderately virulent genotype #8 group between day 5 and 6 p.i., had particularly high viral loads in the brain (2.53 × 10^5^ to 4.77 × 10^8^ VE/mL) and infectious virus was recovered from the euthanized birds. Also a sentinel animal to this group, that was euthanized on day 6 p.i., while suffering from central nervous disorder, had a particular high viral load in the brain (2.21 × 10^9^ VE/mL) (Supplementary Table 2). Lower viral loads were also detected in the lungs, gastrointestinal tract, kidneys, heart, and especially liver, indicating systemic viral spread. Histological examination of three ducklings that succumbed during acute infection, identified a moderate, acute to subacute, necrotizing meningoencephalitis with oligofocal to diffuse antigen detection (score 1–4) showing the same distribution pattern and target cells as described after 3 dpi for genotype #4, except for the spinal cord, that appeared unaffected. Oligofocally, individual ducks tested positive for virus matrix protein in the submucosal glands of the beak, the sinus and air sac as well as in the myocardium associated with necrosis (Supplementary Figure 3; 4). Although lacking virus antigen, the regenerative lesions in the olfactory and sinus epithelium (Supplementary Figure 3) and mild interstitial pneumonia (not shown) indicate past infection. No findings were recorded for the kidneys at 5-6 dpi.

Remarkably, at the end of the experiment on day 21 p.i. residual amounts of viral RNA were still detected by RT-qPCR in the brain of four out of six inoculated surviving animals; but not in other organs tested (Supplementary Table 2). The evaluation of the H&E and IHC slides did not yield any pathologic findings or virus antigen in the remaining tissues (Supplementary Figures 2–4). Similar to the IMPI group of the same genotype, at this late stage, no infectious virus was recoverable. Histological examination did not find any specific lesions in the brain (Fig. 3), but identified an irregular architecture of the olfactory mucosa and squamous metaplasia in the respiratory epithelium of the conchae in 2 out of 3 ducks, lacking antigen labelling (Supplementary Figure 3). Again, this could be an indication of a past infection with regeneration of the affected mucosal areas.

### Genotype-related molecular determinants distinguishing highly from moderately virulent genotypes in ducklings

**Fig. 4:**
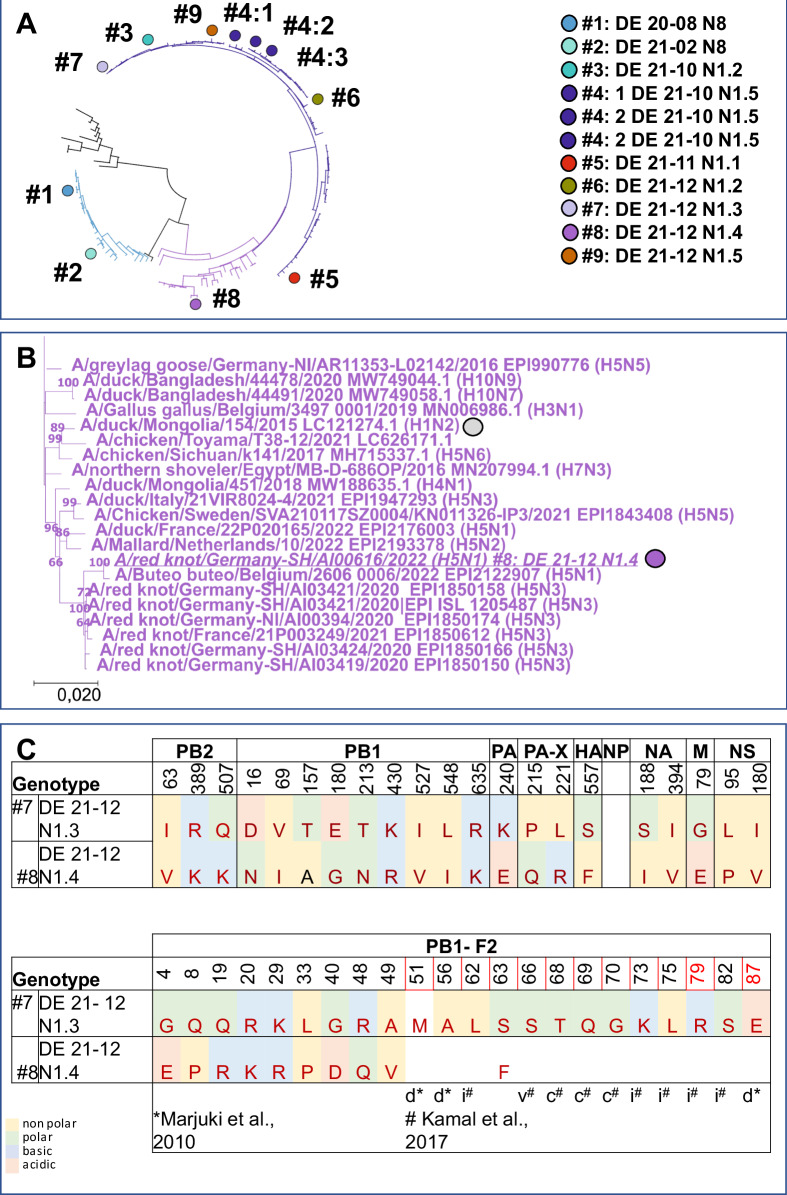
Analysis of PB1. Schematic representation of the phylogenetic relationship based on the PB1 gene of the genotypes tested. The unique color scheme has been used to distinguish genotypes in Germany since 2020, adapted from a custom palette out of the dashboard “HPAIV genotypes in Germany”^22^ (**A**). **B** Shown is a detailed image of the genotype #8 group, presenting related viruses included in the phylogenetic analysis with genotype #8 and the oldest virus with a related PB1 gene indicated by dots (**B**). **C** Differences of deduced amino acids between highly duckling virulent genotype #7 and moderate duckling virulent genotype #8 are given for PB1 and PB1-F2. Sites recognized as being part of a inflammatory motif (i), cytotoxic motif (c) or correlated to virulence in mice but not in ducks (v) are indicated as reviewed^79^ Kamal et al. In addition, sites associated with reduces pathogenicity in ducks (d) as described in ref. ^85^, are indicated but did not show differences.

Molecular comparison of the various HPAIV H5N1 genotypes, as depicted in Fig. 1B, reveals that only 3 genes of genotype #3, the virus that dominated the HPAIV H5N1 epidemic in December 2021, are present in all other viruses. Besides the HA gene only NA- and M-gene of all investigated HPAIV H5N1 viruses are belonging to the same phylogenetic branch. However, the number of reassorted genes varied considerably for the different genotypes studied: For the other two HPAIV H5N1 genotypes with IMPI score of >2, either a single gene (genotype #7: NP) or three genes coding for proteins that are part of the polymerase complex (genotype #4: PB2, PA and NP) differ compared to genotype #3. Likewise, in the group of viruses with moderate duckling pathogenicity (IMPI < 2), an exchange of up to 4 segments could be observed. In addition to genes encoding for proteins of the polymerase complex (PB2, PB1, PA and NP), the NS gene (genotype #9) is derived from genetically divergent branches as genotype #3. The single exception within the group of viruses with moderate duckling virulence, is genotype #8: Compared to highly virulent genotype #7, only the PB1 gene is exchanged. Even though genotypes #7 and #8 share a related NP-gene that differs from genotype #3, this exchange alone obviously did not affect the IMPI. This points to PB1 as an important determinant for the decrease of duckling virulence of genotype #8. Phylogeny of the PB1 gene clearly distinguishes genotype #8 from the other HPAIV H5N1 viruses studied (Fig. 4A). This specific branch of genotype #8 (Fig. 4B) encloses HPAIV H5N3 from the same species than the studied H5N1 genotype #8 virus, i.e. red knots, circulating in 2020 and 2021 in Germany and France^48^. Besides, closely related PB1 genes were present in a variety of subtypes of low pathogenicity like H1, H3, H4 or H10 that were detected in samples from wild birds, with the earliest detection in 2015 (A/duck/Mongolia/154/2015 (H1N2), grey dot). At protein level, genotype #8 virus accumulated altogether nine mutations in PB1 compared to genotype #7 (Fig. 4C). These mutations within the PB1 stretch all over the protein, but did not include the catalytic residues 446 or 447 (Fig. 4C). However, at position 180, located in the nuclear localization motif^49^, glutamic acid in the highly virulent genotype #7 was replaced by a Glycine (E180G) in the moderately virulent genotype #8 (Fig. 4C). Besides, amino acid (aa) substitutions in PB1-F2, a protein translated from an alternative open reading frame (ORF) of segment 2 were present. Altogether, deduced PB1-F2 proteins of genotype #7 and #8 differed in 10 of the 90 aa (11,1%), which is remarkable compared to only 9 different aa in the PB1 ORF (aa 1,2%; 755aa). In contrast, proteins of the other five gene segments had no (NP) or only single point mutations compared to genotype #7 (Supplementary Table 7). This genetic comparison of natural occurring reassortants highlights that multiple genes may affect duckling virulence. Natural phylogenetic analysis in particular of highly and moderate duckling virulent genotype #7 and #8 respectively, indicate that point mutations within PB1 and PB1-F2 may attribute to this phenomenon of shift of duckling virulence.

## Discussion

Numerous different genotypes of HPAIV H5 of clade 2.3.4.4b were circulating in avian species simultaneously in Germany over the last years. When analyzing virulence of an array of genotypes that were detected in the country within week 52 during winter 2021^22,44^, genotype-specific differences in duckling virulence ranging from moderate to highly virulent became evident. In the succeeding months of 2022, two of these genotypes came to dominate the epidemic in Germany in 2022, i.e. genotype #3, and its successor, genotype #4. Both were highly virulent for ducklings with scores of 2.92 and 2.95-3.00 (out of a maximum of 3), respectively. In contrast, less virulent genotypes, such as #5, #6, #8 and #9 with markedly lower IMPI scores ranging from 1.65 down to 1.22 (Fig. 1B; Supplementary Figure 1), faded out in the epidemic of the following months of 2022. In light of these results it is intriguing to argue that genotypes of higher duck virulence may gain epidemiological advantages in wild birds over those with reduced virulence. In this respect our results are not consistent with the ‘avirulence hypothesis’. Alternatively, the “virulence-transmission trade-off” model of virulence evolution of pathogens seems more appropriate in describing the epidemiological situation of HPAIV in Germany in the period 2021/2022. In the latter model, virulence and transmission can be positively correlated at least as long as transmission of a certain viral lineage to other hosts is advantageous compared to other lineages and not limited by the number of susceptible individuals in the given host populations^50,51^, i.e. here the huge multispecies metapopulations of wild aquatic birds and poultry. Such view would be in line with the concept that virulence in a certain host can be higher if a pathogen is a generalist and can easily jump between host species^52^. In this respect, the growing number of susceptible species reported in the course of the recent global spread of the HPAIV 2.3.4.4b virus (now including even species from the Antarctic ecosystem) add to the huge reservoir of a metapopulation of avian hosts accessible to these viruses. In that context, virulence associated with a high case-fatality rate in a host species might not be considered as a limiting factor, but rather as a concomitant of an increased production and release of virus progeny^51^. In fact, a comparison of LPAIV and HPAIV H5N2 viruses in chicken^53^ provided evidence that despite mortality due to HPAIV between day three to day 10 post infection, the reproduction number R_0_ of the HPAIV infection was higher than that of the corresponding LPAIV, leading to early infection of the HPAIV sentinel animals, i.e. between day three and four post infection. Even though the authors acknowledge limitations of their studies with respect to extrapolation to the field, our observations strengthen the view, that all factors promoting transmissibility are key drivers for the current evolution of HPAIV H5. The results of the o.n. infection study that we also performed here, comparing genotype #4 with genotype #8, i.e. a highly prevalent virus with high duckling virulence versus a virus with low prevalence and intermediate duckling virulence, support the latter notion, indicating superior virus shedding of the more virulent genotype also after inoculation along more natural infection routes (Fig. 2). However, it is important to note that individual host-specific factors may also contribute: Several ducks infected with the high virulence genotype #4 proved to be “super-shedders”, while in two animals the viral load in throat swabs was picayune, not exceeding a maximum titre of 2 × 10^3^ VE/mL. In contrast, the two infected sentinel animals reached a titre of 10^10^ VE/mL, a 10^7^-fold difference in virus release (Fig. 2). Beside the quantity of excreted virus, the route of shedding should be considered: While in the group inoculated with high virulence genotype #4 high amounts of virus were present in pharyngeal as well as in cloacal swabs, shedding by the enteral route was almost negligible for the moderate virulence genotype #8. Recently, surface water was demonstrated to be an important medium for HPAIV transmission^54,55^. In conjunction with the notion that shedding of evolutionary successful LPAIV is dominated by the enteral route^28,56^, this finding would clearly emphasize the importance of the enteral route of shedding for the transmission dynamic of HPAIV H5 in wild water birds. The putative relevance of an indirect transmission mode may be another factor in favor of the virulence-transmission trade-off model: If the pathogen is spread by a fomite i.e., by surface water, transmission does not depend on direct interaction of infectious and susceptible individuals. Thus, infection chains can be sustained even though the host was swiftly killed by the pathogen. Dominance of genotypes with high virulence for ducklings would then indicate that, as a net effect, transmission efficacy and high virulence are not negatively correlated for certain clade 2.3.4.4b variants during the enzootic situation in Germany and ultimately contribute to their wide dispersal. However, these assumptions have to be taken with caution, since several limitations of the study have to be considered:

(i) Pekin ducks, as a surrogate of mallards, have been tested here as a single avian species at a very young age: It cannot be excluded that results would differ with other species or other age cohorts. Yet, mallards, being the closest relative in the wild of domestic Pekin ducks, are an abundant species with a global distribution that is deemed to play an important epidemiological role in the field.
(ii) All isolates were derived from diseased or dead hosts, introducing a possible bias towards more virulent genotypes: As such, it seems all the more astonishing that even then, a substantial number of strains have been identified that expressed only moderate pathogenicity in ducklings even in this harsh infection model. In general, HPAIV monitoring in the EU is focused for almost two decades on passive monitoring systems. Few HPAIV detections have been made by active monitoring investigations. Yet, it cannot be excluded that other genotypes exist which predominantly cause clinically mild or even asymptomatic courses of infection that are missed by passive surveillance.
(iii) An inoculation model, the IMPI, was chosen that is not regularly used for HPAIV pathotyping: Routine IVPI pathogenicity testing in chickens most often creates only categorical (yes/no) results (HP or not; dead/alive) that are not useful to pinpoint gradual differences in pathogenicity between strains. In previous experiments^32^ we showed that i.m. inoculation into week-old ducklings, in contrast to IVPI in chickens or occulo nasal application in adult ducks, provided a readout system that allowed a finer distinction of pathogenicity in semiquantitative terms. In this respect age has to be considered. It is well known, that younger ducks are more susceptible to disease^57–59^. Thus, choosing one-week old ducklings represents a sensitive way to distinguish virulence in particular of strains with low or moderate duck virulence like clade 2.3.4.4c or 2.2 viruses^60^. For other strains with high duck virulence, expressing IMPI scores close to the endpoint of 3, like genotypes #1-#4, an infection model using older birds might be more suitable to distinguish differences in virulence^61^. In any case, our results emphasize, that the IMPI is a model system that allows to differentiate duck virulence.
(iv) Intramuscular inoculation is an artificial route and may have skewed the course and outcome of infection: Intramuscular infection is a well approved infection mode for testing vaccine efficacy according the European pharmacopoeia, for example for Newcastle disease. Like the IVPI, it is a parenteral application, thereby circumventing primary local replication at the entry site. A faster systemic spread could explain the differences in onset of a clinical manifestation that was indeed present when testing genotypes #4 and #8 in parallel trials where the same virus has been inoculated occulo-nasally and i.m. However, principal differences in virulence between genotypes were evident regardless of the inoculation mode (parallel testing of o.n. and i.m. routes for genotypes #4 and #8 (Fig. 2)) but the IMPI model provided differentiation of virulence in a pronounced way.
(v) The inoculation dose was not normalized between the groups and differed between 2.86 × 10^3^ (genotype #2; IMPI 2.69) and 5.48 × 10^7^ TCID_50_ (genotype # 4:2; IMPI 2.95) (Table 1): Like in the diagnostic IVPI, we followed the rule that a virus stock should have an HA-titer of >16. Under this rule we observed that the viruses with the lowest (#2) and the highest (#4) viral titers induced almost the same IMPI despite a gap in infectivity of 4 log_10_ steps. This would strengthen the notion deduced from the official (legal) IVPI testing that the titre of infectivity, i.e. the inoculum dose is not a key determinant for pathogenicity provided it exceeds a certain threshold. Indeed, this assessment would be in line with observations from titration experiments in ducks with a clade 2.3.4.4b HPAIV H5N1^29,61^. In this example (Spackman et al.^29^), onset and severity of clinical signs after occulo- nasal infection were indistinguishable for groups inoculated with a dose of 10^4^ or 10^6^ TCID_50_. However, in birds inoculated with 6.3 × 10^1^ TCID_50_ per bird, onset of clinical disease started two days later; this would clearly influence a clinical score as applied here. In conclusion, this would support the notion that the inoculum has to exceed a certain threshold, beyond which a clinical disease index is no longer correlated to the amount of infectivity in the inoculum. Yet, for further standardization of similar experiments, a minimum amount of 10^5^ TCID_50_ should be recommended.

Investigating pathogenesis for high and moderate virulence genotypes #4 and #8, respectively, after occulo-nasal application, verified differences of virulence between the two genotypes, and demonstrated a slower progression to disease after o.n. compared to i.m. virus application: Whereas after i.m. application of genotype #4, nine of ten ducks were dead by 1 dpi, clinical signs started in the o.n. group at 2 dpi, but became apparent only in three of ten ducklings. Likewise, for genotype #8, incubation time was prolonged from 3 dpi to 5 dpi for the i.m. and o.n. inoculated groups, respectively, before individual birds showed signs of disease. However, regardless of the genotype, it became evident, that all diseased birds suffered from a systemic infection, as demonstrated by virus detection in various organs. Abundant viral replication was detected for both genotypes in the brain, with a dominance of viral antigen during the acute phase in the forebrain. Most interestingly, viral antigen was present in histopathological examinations of two out of three i.m. inoculated ducklings infected with moderate duckling virulence genotype #8, at 10 dpi, i.e. at a time when none of the animals showed any clinical signs of disease. Antigen detection was accompanied by severe, subacute, necrotizing meningoencephalitis in both antigen-positive birds. However, the third/remaining duckling presented moderate inflammation, lacking intralesional virus antigen, indicating a potential natural healing process. These observations would argue, that also in animals showing only moderate clinical signs of disease after genotype #8 infection, virus invasion into the CNS was sufficiently controlled. The conclusion of limited brain infection of the moderately virulent genotype #8 could explain the puzzling observation in o.n. inoculated ducklings euthanized at the end of the experiment at 21 dpi, where residual amounts of viral RNA were detected in the brain without histological lesions (Fig. 3). The latter indicates that for some animals, brain invasion was prevented or at least significantly limited after occulo-nasal infection. The rapid onset of infection control across the blood-brain barrier would support the notion that it is related to innate immunity, and independent of the specific immune response^62,63^. In our view, neuronal infection with a virus of moderate virulence for ducklings could provide a valuable system to study these virus-host interactions.

For the majority of genotypes with reduced duckling virulence, several genome segments differed in comparison to dominant high virulence genotype #3. In three of the four genotypes with decreased duckling virulence, i.e. genotypes #5, #6 and #9, at least two of three segments coding for the viral RNA-dependent RNA polymerase (RdRP)^64,65^ were changed, i.e Polymerase basic protein 2 (PB2), Polymerase basic protein 1 (PB1), and Polymerase acidic protein (PA) respectively. In addition, the nucleoprotein (NP) was different compared to genotype #3. NP, forming a helical scaffold that packages and protects the viral genome^66^, is associated to viral RNA (vRNA) and together with the RdRP is the integral part of the viral ribonucleoprotein (vRNP) complex. These observed changes of segments coding for the vRNP complex indicates a contribution to decreased duckling virulence, a notion that is supported by experimental data for other strains^67–71^. However, genotype #4, the genotype that subsequently dominated HPAIV H5N1 epidemiology in 2022 (see Fig. 1B), also differed with respect to RdRP, i.e. PB2, PA and NP, compared to genotype #3 (see Fig. 1A). Considering the complex interactions of RdRP in conjunction with its interplay with NP (for review, see^72,73^), it is conceivable that a new composition in reassorted genotypes affects viral transcription and replication, and thus virulence, in a pleiotropic manner. In this context, differences in virulence would not only be associated with a particular segment, but could also result from the context with other components of the replication machinery, i.e. the gene constellation. In addition, segment 8, which encodes non-structural protein 1 (NS1) and nuclear export protein (NEP), was different in genotype #9 compared to genotype #3 and may have contributed to the reduced virulence in ducklings, as shown in previous studies for other strains^74^.

An exception from this observation is the moderate virulence genotype #8 in which only a single segment, i.e. PB1, differed from the high virulence genotype #7. Specific mutations within PB1 have been reported to facilitate an expanded host range^64^ or to convey adaption to host innate immunity^75^. One striking difference was observed at position 180 (E180G), a site identified as one of two mutations within PB1 (E180D, M317V) that were shown to increase polymerase activity in chicken cells and plaque sizes in chicken and duck cells^71^. On the other side, a higher polymerase activity may not be directly correlated to virulence by producing higher virus loads as indicated by studies on HPAIV H5N8 viruses from outbreaks in 2014–15 to 2016–17, but rather influenced virulence through species-specific levels of induced IFN-ß^76^. This observation is in line with in vivo data, comparing innate immune responses in ducks and chicken with regard to virulence^77^. It remains to be investigated whether this phenomenon is linked to the observed differences in virulence of these specific genotypes.

Likewise, differences in PB1-F2 of moderately virulent genotype #8 are noteworthy. This protein, translated from an alternative open reading frame (ORF) of segment 2^78^ is associated to both pro-apoptotic and polymerase enhancing functions^79,80^: In all tested HP AIV H5N1, but not in the H5N8 viruses (Supplementary Table 8), full length PB1-F2 ORF was present, a finding that is in line with the observations that the ORF is well-conserved in avian influenza virus isolates^81^ and reported to be highly virus-isolate specific with respect to influence on virulence^80^. Studies in chicken indicate, that presence of PB1-F2 contributes to an optimized spread of the virus without increasing the virulence^82,83^, while other studies describe a slightly prolonged survival time of chickens infected with PB1-F2 deletion mutants^84^ and delayed onset of both clinical symptoms and systemic spread of virus in ducks^70^. Besides, aa substitutions in the N-terminal domain of PB1-F2, at positions T51M, V56A and E87G were accompanied with reduced virulence for mallard ducks and shortened time of virus shedding^85^. However, both genotypes, i.e. #7 an #8, that differed in segment 2, had an identical aa composition in these three sites, sharing two sites associated with lowered virulence and one aa site with increased virulence (underlined aa). Combined with the observed differences in duckling virulence, our results would justify further work on the role of PB1 /PB1-F2 in species-specific virulence in Anseriformes.

Analysis of naturally occurring reassortant genotypes of clade 2.3.4.4b HPAIV H5 suggests that high virulence in ducklings was not a limiting factor for epidemiological dominance in Germany. From a swarm of seven concurrently circulating HPAIV H5N1, it appeared that viruses with high duckling virulence were the most successful in terms of geographical spread and duration of circulation^22^, while genotypes with lower virulence remained in the minority during outbreaks and were detected in very limited geographical areas only. Therefore, assuming that the virulence-transmission trade-off model is more consistent with the emergence and dominance of high virulence strains, this would suggest that the size of the susceptible population is not currently a limiting factor for HPAIV evolution. Thus, these strains can be expected to retain high duckling virulence if this continues to promote improved transmissibility, as suggested for the strains studied here. Consequently, breaking this vicious circle would require a reduction in the susceptible population. In wild birds, this can only be achieved by gradually increasing population immunity mediated by survivors of previous infections. In poultry, however, this could be achieved by vaccination.

## Supplementary information

H5_genotypes_ARRIVE Compliance Questionnaire

Supplementary materials_revised

## Data Availability

Data is presented within the paper or in the supplementary files. All reference sequences are available on Zenodo under 10.5281/zenodo.8233814. Sequences in this study are available in the GISAID EpiFluTM database under accession numbers: A/chicken/Germany-NW/AI03705/2021 (accession 5146288), A/seal/Germany-SH/AI05373/2021 (accession 4804850), A/European herring gull/Germany-MV/AI01411/2022 (accession 18006762), A/chicken/Germany-NI/AI04286/2022 (accession 16096050), A/herring gull/Germany-SH/AI01196/2022 (accession 17693298), A/brent goose/Germany-SH/AI02407/2022 (accession 17693301), A/herring gull/Germany-SH/AI07088/2021 (accession 17693297), A/chicken/Germany-MV/AI01026/2022 (accession 18006937), A/pigeon/Germany-NW/AI00951/2022 (accession 10261376), A/red knot/Germany-SH/AI00616/2022 (accession 5098132), A/black-headed gull/Germany-HH/AI01073/2022 (accession 18006938).

## Code availability

GraphPad Prism in software version 8.0.1 (GraphPad Software, San Diego, CA, USA) was used for statistical analyses. SigmaPlot™ in version 11 (SigmaPlot Software, Grafiti LCC, Palo Alto, CA, USA) was also used to double-check the statistical data. Hamamatsu S60 scanner (Hamamatsu Photonics, K.K. Japan) and software NDPview.2 plus in version 2.8.24 were used for histopathological analyses. Geneious Prime in version 2021.0.1, as well as the tool Minimap2 in version v2.24 as well as the phylogenetic analysis tool MEGA X: Molecular Evolutionary Genetics Analysis across computing platforms software version 10.2.4 (Kumar, Stecher, Li, Knyaz, and Tamura 2018)^45^ were used for sequence analyses. Further data on sequences etc. are provided within the supplemental material as well as available upon request.
