# Author Correction: Dominant HPAIV H5N1 genotypes of Germany 2021/2022 are linked to high virulence in Pekin ducklings

**DOI:** 10.1038/s44298-024-00071-z

**Published:** 2024-11-18

**Authors:** Ronja Piesche, Angele Breithaupt, Anne Pohlmann, Ann Kathrin Ahrens, Martin Beer, Timm Harder, Christian Grund

**Affiliations:** 1 Friedrich- Loeffler- Institute, Institute of Diagnostic Virology, Greifswald, Germany; 2 Friedrich- Loeffler- Institute, Department of Experimental Animal Facilities and Biorisk Management (ATB), Greifswald, Germany

**Keywords:** Virology, Influenza virus

Correction to: *npj Viruses* 10.1038/s44298-024-00062-0, published online 6 November 2024

In this article the funding from ‘Kappa-Flu project, under the Horizon Europe Program (grant agreement KAPPA-FLU no. 101084171)’ was omitted. The original article has been corrected.

