# Author Correction: Dominant HPAIV H5N1 genotypes of Germany 2021/2022 are linked to high virulence in Pekin ducklings

**DOI:** 10.1038/s44298-025-00103-2

**Published:** 2025-02-27

**Authors:** Ronja Piesche, Angele Breithaupt, Anne Pohlmann, Ann Kathrin Ahrens, Martin Beer, Timm Harder, Christian Grund

**Affiliations:** 1 Friedrich- Loeffler- Institute, Institute of Diagnostic Virology, Greifswald, Germany; 2 Friedrich- Loeffler- Institute, Department of Experimental Animal Facilities and Biorisk Management (ATB), Greifswald, Germany

**Keywords:** Virology, Influenza virus

Correction to: *npj Viruses* 10.1038/s44298-024-00062-0, published online 06 November 2024

In this article, the funding statement from the European Union (WiLiMan-ID, grant agreement 101083833) was omitted. The original article has been corrected.

