# Supplementary material for: Dominant HPAIV H5N1 genotypes of Germany 2021/2022 are linked to high virulence in Pekin ducklings
Source: Npj Viruses. 2024 Nov 6;2:53. doi: 10.1038/s44298-024-00062-0 (PMC11721377; doi:10.1038/s44298-024-00062-0)
Supplement: Supplementary file 2 — Supplementary materials_revised [file 44298_2024_62_MOESM2_ESM.pdf]

A

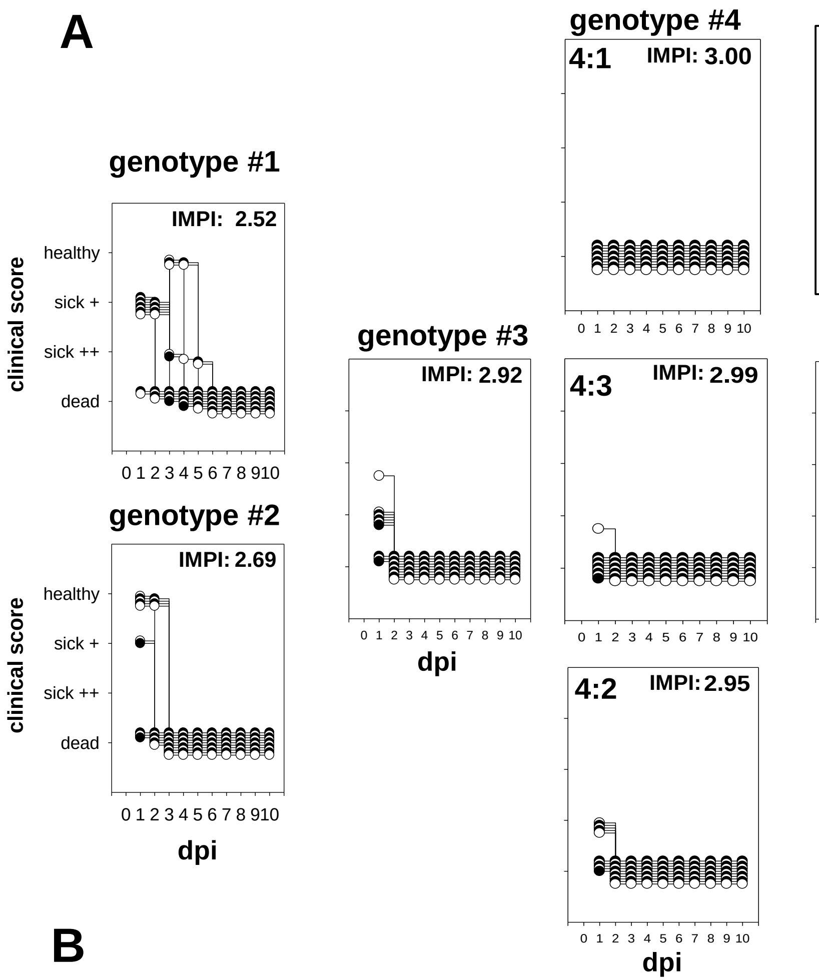
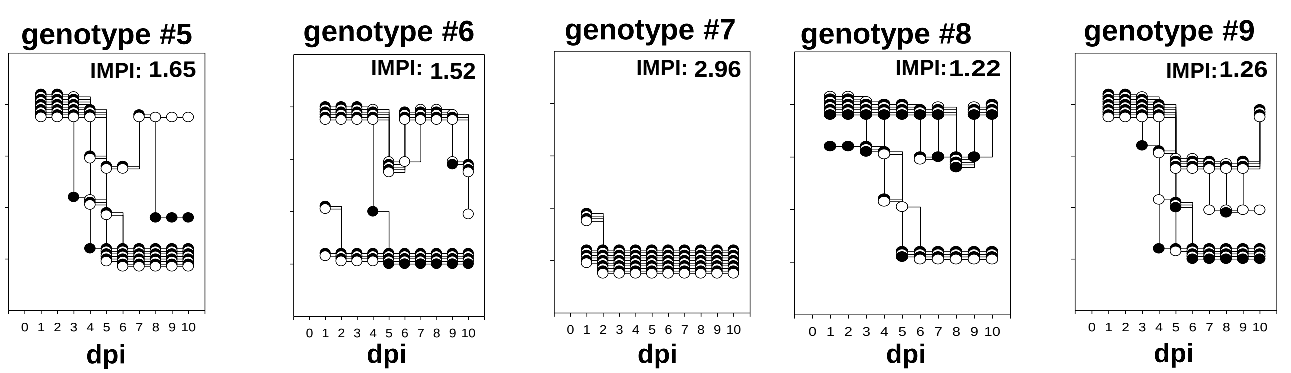


***Supplemental figure 1.***
**Clinical course of ducklings after i.m. inoculation with HPAIV H5Nx genotypes of clade 2.3.4.4b.**
(A) Depicted are individual daily clinical scores after i.m inoculation of seven day old ducklings (Anas plathyrynchos) with indicated genotypes. The resulting IMPI scores are given at the top of each box. (B) Statistical significance was determined by applying Mann- Whitney- U tests using GraphPad Prism version 8.0.1. The table gives p- values as expression for statistical significance. Effect size rU for Mann-Whitney U statistics was calculated using the formula according to Wendt (1972) and can assume a positive value between 0-1, where 1 corresponds to the highest achievable value.

B

| p- value | #1 | #2 | $r_U$ | #3 | $r_U$ | #4:1 | $r_U$ | #4:2 | $r_U$ | #4:3 | $r_U$ | #5 | $r_U$ | #6 | $r_U$ | #7 | $r_U$ | #8 | $r_U$ | #9 | $r_U$ |
|---|---|---|---|---|---|---|---|---|---|---|---|---|---|---|---|---|---|---|---|---|---|
| **#1** | | 0.3315 | 0.3 | 0.0063 | 0.7 | 0.0026 | 0.8 | 0.0031 | 0.7 | 0.0004 | 0.8 | 0.002 | 0.8 | 0.3191 | 0.3 | 0.0024 | 0.6 | 0.0012 | 0.8 | 0.0001 | 0.8 |
| **#2** | | | | 0.0661 | 0.5 | 0.0254 | 0.7 | 0.0002 | 0.6 | 0.0031 | 0.7 | <0.0001 | 1 | 0.1519 | 0.4 | 0.0159 | 0.8 | | | <0.0001 | 1 |
| **#3** | | | | | | 0.0031 | 0.7 | 0.4499 | 0.3 | 0.0174 | 0.6 | <0.0001 | 1 | 0.0516 | 0.5 | 0.1462 | 0.4 | <0.0001 | 1 | <0.0001 | 1 |
| **#4:1** | | | | | | | | 0.0325 | 0.5 | >0.9999 | 0.1 | <0.0001 | 1 | 0.0007 | 0.8 | 0.0867 | 0.3 | <0.0001 | 1 | <0.0001 | 1 |
| **#4:2** | | | | | | | | | | 0.1409 | 0.4 | <0.0001 | 1 | 0.018 | 0.6 | 0.6499 | 0.2 | <0.0001 | 1 | <0.0001 | 1 |
| **#4:3** | | | | | | | | | | | | <0.0001 | 1 | 0.0019 | 0.8 | 0.3034 | 0.2 | <0.0001 | 1 | <0.0001 | 1 |
| **#5** | | | | | | | | | | | | | | 0.9574 | 0 | <0.0001 | 1 | 0.4306 | 0.2 | 0.22 | 0.4 |
| **#6** | | | | | | | | | | | | | | | | 0.0093 | 0.7 | 0.9507 | 0 | >0.9999 | 0 |
| **#7** | | | | | | | | | | | | | | | | | | <0.0001 | 1 | <0.0001 | 1 |
| **#8** | | | | | | | | | | | | | | | | | | | | 0.8871 | 0 |
| **#9** | | | | | | | | | | | | | | | | | | | | | |

p- value below < 0.05

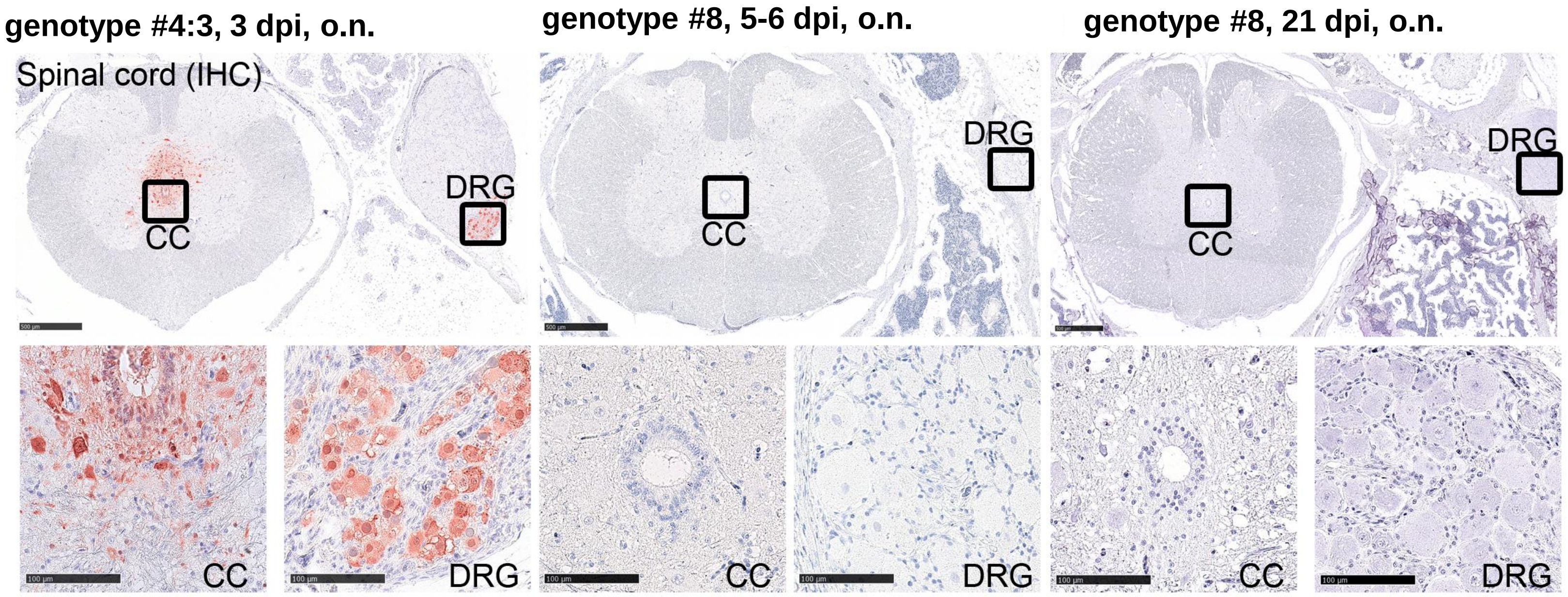


***Supplemental Figure 2.***
Pathological findings and Influenza A virus antigen detection in the spinal cord.
Influenza A virus antigen detection in the central canal (CC) of the spinal cord and dorsal root ganglia (DRG) of ducks inoculated with genotype #4:3 or genotype #8. Virus antigen in the epithelium of the CC, in adjacent neurons and glial cells as well as subordinate neurons in the DRG only after genotype #8. Dpi, days post infection; o.n., occulo-nasally; immunohistochemistry IHC, central canal (CC), dorsal root ganglia (DRG). Bar 500µm (overview) and 100 µm (detail).

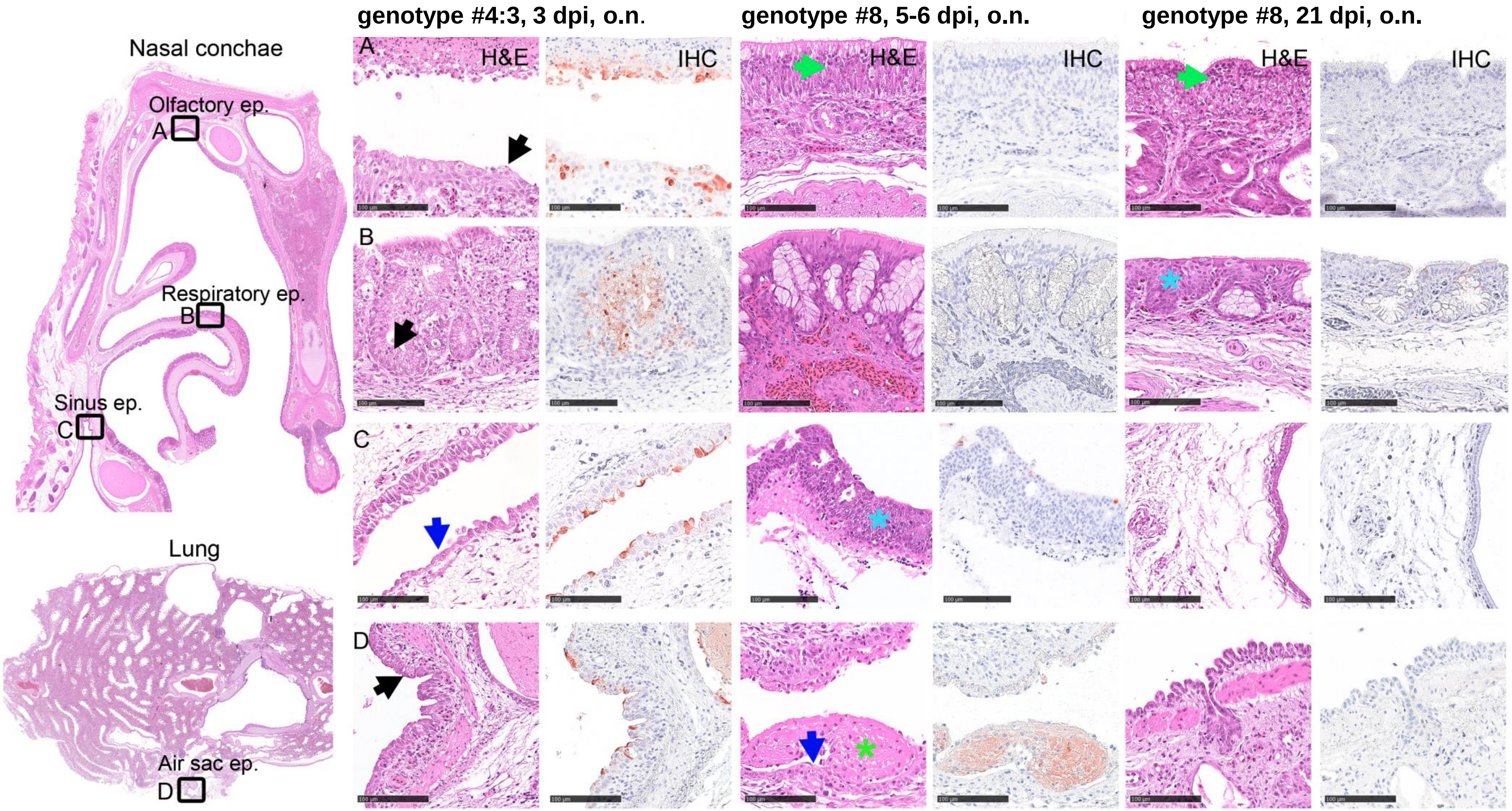


***Supplemental Figure 3.***
Pathological findings and Influenza A virus antigen detection in respiratory tract.
Pathological findings and Influenza A virus antigen detection in the respiratory tract of ducks inoculated with genotype #4:3 or genotype #8. Severe, acute necrotizing rhintis, sinusitis and airsacculitis affecting the olfactory epithelium (A) and respiratory epithelium of conchae (B) and sinus (C) and air sac (D) with abundant intralesional virus antigen detected 3 days after genotype #4:3 inoculation. Black arrows indicate acute epithelial necrosis, blue arrow points to flattened sinus epithelium covering the loss of necrotic mucosal epithelium. Oligofocal antigen found 5-6 days after genotype #8 inoculation in the sinus epithelium (C), the submucosal glands (not shown) and necrotic debris of air sac (D, green asterisk) but not in ducks, 21 dpi. The green arrows point to the irregular olfactory epithelium, the blue asterisk shows squamous hyperplasia/metaplasie, the blue arrow points to flattened air sac epithelium, indicating regeneration of previous infection sites 5-6 dpi and 21 dpi. Dpi, days post infection; hematoxylin-eosin stain, H&E; immunohistochemistry IHC; ep., epithelium. Bar 100µm.

**genotype #4:3, 3 dpi, o.n.**

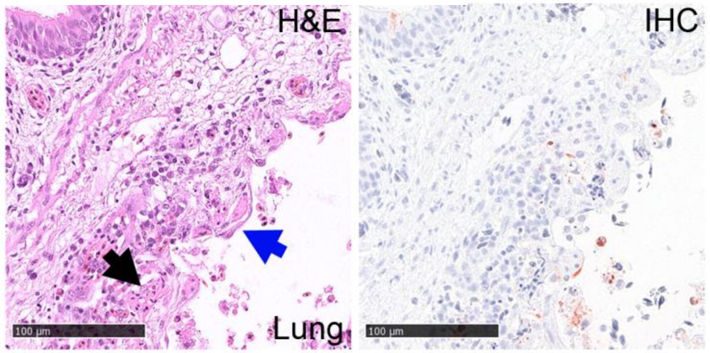


***Supplemental Figure 3.1.***

Pathological findings and Influenza A virus antigen detection in the lung of ducks inoculated with genotype #4.

Severe, acute necrotizing pneumonia with intralesional virus antigen detected 3 days after inoculation in bronchial, parabronchial and capillary epithelium of the lung. Black arrow indicates acute epithelial necrosis, blue arrow points to flattened parabronchial epithelium covering the loss of necrotic epithelium. Dpi, days post infection; hematoxylin-eosin stain, H&E; immunohistochemistry IHC. Bar 100µm.

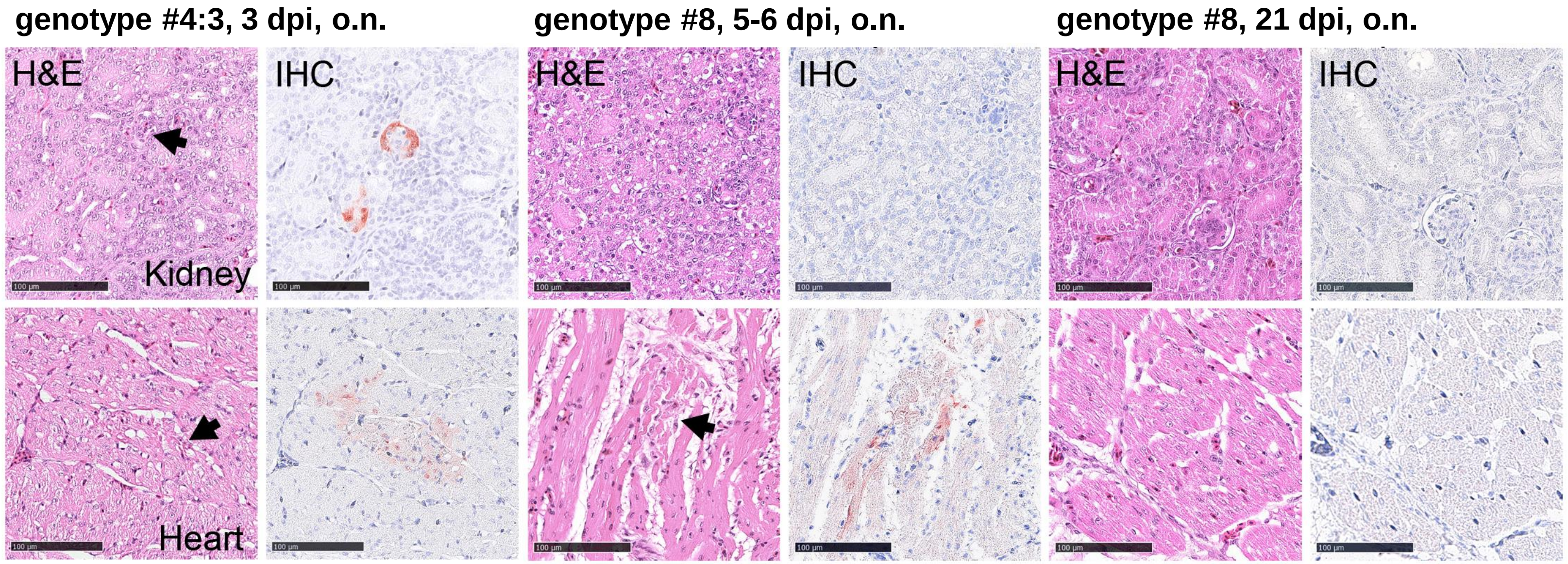


***Supplemental Figure 4.***
Pathological findings and Influenza A virus antigen detection in the kidney and heart.
Depicted are oligofocal acute necrosis of renal tubular epithelium (black arrow) and myocardium (black arrow) with intralesional virus antigen detection 3 days after genotype #4 or 5-6 days after genotype #8 (heart only) inoculation, but not in clinical healthy ducks, 21 dpi. Dpi, days post infection; hematoxylin-eosin stain, H&E; immunohistochemistry IHC. Bar 100µm.

**Supplementary Table 1. Occurrence and distribution of HPAIV H5 genotypes in Germany from october 2021 until the end of 2022.**

| | **Genotype** | **number of entries** | | | **2021** | | **2022** | | **2021-2022** | |
|---|---|---|---|---|---|---|---|---|---|---|
| | **DE[1]** | **captive** | **poultry** | **Wild** | **w40- w52** | **%** | **w1-52** | **%** | | **%** |
| 1 | DE 21-10 N1.1 | | 4 | 4 | 8 | 5.8 | 0 | 0.0 | 8 | 2.2 |
| 2 | **DE 21-10 N1.2** | 5 | 27 | 88 | 60 | 43.8 | 1 | 0.6 | 120 | 33.4 |
| 3 | DE 21-10 N1.3 | | | 1 | 1 | 0.7 | 0 | 0.0 | 1 | 0.3 |
| 4 | DE 21-10 N1.4 | | | 1 | 1 | 0.7 | 0 | 0.0 | 1 | 0.3 |
| 5 | **DE 21-10 N1.5** | 4 | 91 | 87 | 43 | 31.4 | 133 | 81.6 | 176 | 49.0 |
| 8 | **DE 21-11 N1.1** | | 1 | 2 | 3 | 2.2 | 0 | 0.0 | 3 | 0.8 |
| 9 | DE 21-11 N1.2 | | 2 | 1 | 3 | 2.2 | 0 | 0.0 | 3 | 0.8 |
| 10 | DE 21-11 N1.3 | | 2 | 2 | 4 | 2.9 | 0 | 0.0 | 4 | 1.1 |
| 11 | DE 21-11 N1.4 | | | 1 | 1 | 0.7 | 0 | 0.0 | 1 | 0.3 |
| 12 | DE 21-11 N1.5 | | 1 | | 1 | 0.7 | 0 | 0.0 | 1 | 0.3 |
| 13 | DE 21-11 N1.6 | | | 1 | 1 | 0.7 | 0 | 0.0 | 1 | 0.3 |
| 14 | DE 21-12 N1.1 | | 1 | | 1 | 0.7 | 0 | 0.0 | 1 | 0.3 |
| 15 | **DE 21-12 N1.2** | | 5 | 5 | 3 | 2.2 | 7 | 4.3 | 10 | 2.8 |
| 16 | **DE 21-12 N1.3** | | 1 | 4 | 1 | 0.7 | 4 | 2.5 | 5 | 1.4 |
| 17 | **DE 21-12 N1.4** | | | 3 | 1 | 0.7 | 2 | 1.2 | 3 | 0.8 |
| 18 | **DE 21-12 N1.5** | | 5 | 1 | 4 | 2.9 | 2 | 1.2 | 6 | 1.7 |
| 19 | DE 21-12 N1.6 | | | 2 | 1 | 0.7 | 1 | 0.6 | 2 | 0.6 |
| 20 | DE 22-01 N1.1 | | | | | 0.0 | 2 | 1.2 | 2 | 0.6 |
| 21 | DE 22-01 N1.2 | | | | | 0.0 | 1 | 0.6 | 1 | 0.3 |
| 22 | DE 22-01 N2.1 | | | | | 0.0 | 2 | 1.2 | 2 | 0.6 |
| 23 | DE 22-04 N1.1 | | | | | 0.0 | 1 | 0.6 | 1 | 0.3 |
| 24 | DE 22-09 N1.2 | | | | | 0.0 | 5 | 3.1 | 5 | 1.4 |
| 25 | DE 22-11 N1.1 | | | 2 | | 0.0 | 2 | 1.2 | 2 | 0.6 |
| | | 9 | 140 | 205 | 137 | | 163 | 100 | 359 | 100 |

[1] country code according to ISO 3166

* https://doi.org/10.5281/zenodo.6838094

**xy** Genotypes characterized here

xy no virus could be harvested/ isolated

**Supplementary Table 2. Viral load (Viral equivalents/ mL) of organs and swab samples of ducklings inoculated occulo- nasally with Genotype #4:3 vs. Genotype #8.**

**o.n. infection** — **genotype #4 :3**

| | animal | #42 (3dpi) | #43 (3dpi) | #44 (3dpi) | #45 (3dpi) | #46 (3dpi) | #47 (3dpi) | #48 (3dpi) | #50 (3dpi) | #99 (3dpi) | #49 (4dpi) | #549 [S] (5dpi) | #550 [S] (5dpi) | mean | SD |
|---|---|---|---|---|---|---|---|---|---|---|---|---|---|---|---|
| | VE/ml | | | | | | | | | | | | | | |
| **Organs** | brain | 6.03E+03 | 7.64E+02 | 1.74E+05 | 1.56E+03 | 2.45E+02 | 2.42E+02 | 6.83E+01 | 1.51E+03 | 3.29E+05 | 1.38E+03 | 3.64E+05 | 2.07E+02 | 7.32E+04 | 1.31E+05 |
| | lungs | 4.13E+04 | 4.93E+04 | 2.15E+04 | 2.57E+04 | 1.36E+05 | 1.42E+04 | 6.92E+01 | 2.42E+02 | 1.59E+02 | 2.77E+02 | 7.05E+05 | 2.46E+05 | 1.03E+05 | 1.94E+05 |
| | DD | 1.06E+05 | 8.16E+04 | 2.53E+04 | 1.43E+05 | 1.40E+05 | 4.90E+04 | 7.56E+01 | 3.60E+03 | 2.49E+02 | 5.30E+03 | 4.78E+05 | 4.81E+02 | 8.60E+04 | 1.29E+05 |
| | kidney | 3.79E+05 | 1.86E+06 | 7.44E+05 | 7.73E+04 | 2.04E+05 | 7.42E+04 | 5.73E+04 | 2.21E+04 | 2.09E+03 | 2.79E+04 | 1.82E+04 | 1.64E+06 | 4.26E+05 | 6.28E+05 |
| | liver | 1.34E+02 | 2.11E+02 | 1.58E+02 | 1.39E+03 | 2.77E+02 | 4.93E+04 | 3.35E+04 | 1.22E+05 | 3.95E+02 | 6.84E+04 | 2.05E+03 | 4.74E+02 | 2.32E+04 | 3.72E+04 |
| | heart | 2.72E+02 | 2.15E+03 | 2.31E+02 | 1.53E+02 | 2.45E+02 | 4.06E+02 | 1.26E+06 | 4.17E+05 | 3.96E+03 | 2.03E+05 | 1.75E+03 | 5.25E+04 | 1.61E+05 | 3.51E+05 |
| **Excretion** | swab choana 1dpi | neg | 1.75E+03 | 4.73E+06 | 1.83E+07 | 3.20E+06 | 3.46E+07 | 2.26E+07 | 1.21E+06 | 3.92E+03 | 3.53E+06 | X | X | 9.80E+06 | 1.17E+07 |
| | swab choana 2dpi | 2.71E+03 | 2.53E+03 | 4.76E+08 | 6.19E+08 | 1.11E+07 | 4.52E+08 | 4.48E+08 | 2.02E+08 | 7.08E+05 | 1.80E+07 | 4.42E+06 | 5.26E+06 | 1.86E+08 | 2.31E+08 |
| | swab choana 3dpi | 1.86E+03 | 7.12E+02 | 6.20E+06 | 3.61E+06 | 2.64E+06 | 8.70E+06 | 5.10E+06 | 9.59E+06 | 4.44E+05 | 7.38E+04 | 8.06E+08 | 1.02E+10 | 9.20E+08 | 2.81E+09 |
| | swab choana 4dpi | X | X | X | X | X | X | X | X | X | 5.56E+07 | 2.80E+06 | 2.22E+07 | 2.69E+07 | 2.18E+07 |
| | swab choana 5dpi | X | X | X | X | X | X | X | X | X | X | 2.38E+10 | 4.09E+05 | 1.19E+10 | 1.19E+10 |
| | swab cloaca 1 dpi | 1.69E+03 | 3.46E+02 | 1.46E+08 | 1.54E+08 | 1.01E+08 | 1.38E+08 | 6.33E+08 | 4.11E+09 | 1.05E+10 | 1.85E+06 | X | X | 1.58E+09 | 3.21E+09 |
| | swab cloaca 2 dpi | 5.92E+03 | 2.53E+03 | 1.03E+07 | 7.95E+06 | 2.65E+05 | 8.03E+07 | 8.01E+06 | 1.18E+06 | 3.39E+07 | 5.98E+09 | 1.14E+06 | 3.48E+06 | 5.11E+08 | 1.65E+09 |
| | swab cloaca 3 dpi | 5.83E+03 | 6.03E+09 | 1.08E+10 | 1.45E+10 | 6.40E+09 | 7.90E+09 | 5.39E+09 | 1.32E+07 | 1.08E+08 | 5.73E+07 | 2.26E+07 | 2.37E+08 | 4.29E+09 | 4.79E+09 |
| | swab cloaca 4 dpi | X | 1.61E+03 | X | X | X | X | X | X | X | 7.88E+08 | 1.29E+06 | 2.14E+06 | 1.98E+08 | 3.41E+08 |
| | swab cloaca 5 dpi | X | X | X | X | X | X | X | X | X | X | 6.66E+07 | 1.05E+10 | 5.29E+09 | 5.22E+09 |

* cut- off $1.64*10^3$

**o.n. infection** — **genotype #8**

| | animal | #392 (5 dpi) | #395 (5 dpi) | #399 (6 dpi) | #400 (6 dpi) | #390 (21dpi) | #391 (21dpi) | #393 (21dpi) | #396 (21dpi) | #397 (21dpi) | #398 (21dpi) | #66 [S] (6 dpi)* | #64 [S]* (21dpi) | mean | SD |
|---|---|---|---|---|---|---|---|---|---|---|---|---|---|---|---|
| | VE/ml | | | | | | | | | | | | | | |
| **Organs** | brain | 2.53E+05 | ****4776198413,46** | ****1799205033,12725** | ****3699896769,34585** | neg | 4.40E+04 | 8.99E+02 | 4.94E+03 | 3.75E+03 | neg | ****2203649653,10531** | neg | 6.13E+04 | 9.72E+04 |
| | lungs | neg | 2.17E+06 | 2.79E+05 | 3.33E+04 | neg | neg | neg | neg | neg | neg | 6.07E+04 | neg | 6.35E+05 | 8.90E+05 |
| | DD | 2.19E+02 | 8.00E+03 | 1.22E+06 | 5.83E+03 | neg | neg | neg | neg | neg | neg | 2.96E+02 | neg | 2.46E+05 | 4.85E+05 |
| | kidney | 1.36E+03 | 1.43E+05 | 1.25E+05 | 6.19E+03 | neg | neg | neg | neg | neg | neg | 1.85E+04 | neg | 5.88E+04 | 6.19E+04 |
| | liver | neg | 5.01E+05 | 8.01E+06 | 1.34E+05 | neg | neg | neg | neg | neg | neg | 1.02E+06 | neg | 2.42E+06 | 3.24E+06 |
| | heart | 4.59E+03 | 8.29E+05 | 1.15E+05 | 5.81E+04 | neg | neg | neg | neg | neg | neg | 1.93E+05 | neg | 2.40E+05 | 3.01E+05 |
| **Excretion** | swab choana 1dpi | 2.77E+03 | 2.87E+05 | neg | neg | 4.27E+04 | 4.60E+04 | 2.07E+03 | 2.63E+03 | 2.35E+03 | 5.13E+03 | X | X | 4.89E+04 | 9.19E+04 |
| | swab choana 2dpi | neg | 1.47E+05 | 1.31E+05 | 6.75E+04 | 5.68E+04 | 3.55E+05 | neg | neg | 2.82E+06 | 1.95E+03 | neg | 1.96E+03 | 4.48E+05 | 9.03E+05 |
| | swab choana 3dpi | 1.02E+06 | 2.16E+07 | 2.97E+06 | 2.65E+06 | 7.69E+05 | 6.74E+06 | 2.22E+06 | 6.01E+03 | 1.25E+06 | 9.68E+04 | 5.49E+03 | 5.53E+03 | 3.28E+06 | 5.82E+06 |
| | swab choana 4dpi | 1.22E+06 | 2.27E+06 | 1.03E+05 | 9.68E+04 | 6.31E+04 | 5.75E+06 | 1.14E+05 | 5.18E+06 | 5.05E+05 | 6.09E+05 | 4.81E+04 | 3.43E+04 | 1.33E+06 | 1.96E+06 |
| | swab choana 5dpi | 1.30E+04 | 7.04E+03 | 1.02E+05 | 2.39E+04 | neg | 2.84E+04 | 8.00E+03 | 3.29E+05 | 2.07E+03 | 3.73E+04 | 4.03E+05 | 3.96E+04 | 9.03E+04 | 1.34E+05 |
| | swab choana 6 dpi | 1.61E+03 | X | X | neg | neg | neg | neg | 5.27E+04 | neg | neg | 1.55E+06 | 2.25E+04 | 4.06E+05 | 6.59E+05 |
| | swab choana 7 dpi | X | X | X | X | neg | neg | neg | 1.66E+03 | neg | neg | X | neg | | |
| | swab cloaca 1 dpi | neg | neg | neg | neg | neg | neg | neg | 2.50E+03 | neg | neg | X | X | | |
| | swab cloaca 2 dpi | neg | neg | neg | 3.13E+03 | neg | neg | neg | 6.34E+03 | 2.18E+03 | neg | neg | 4.59E+03 | 4.06E+03 | 1.57E+03 |
| | swab cloaca 3 dpi | 1.23E+04 | 2.57E+03 | neg | 2.29E+04 | neg | neg | 1.99E+03 | 1.18E+04 | neg | 4.16E+03 | 2.84E+03 | neg | 8.36E+03 | 7.17E+03 |
| | swab cloaca 4 dpi | neg | 2.01E+04 | neg | neg | neg | neg | 5.21E+03 | 1.58E+03 | neg | neg | neg | neg | 8.98E+03 | 8.03E+03 |
| | swab cloaca 5 dpi | neg | neg | neg | neg | neg | neg | neg | neg | neg | neg | neg | neg | | |
| | swab cloaca 6 dpi | X | X | X | 3.37E+03 | neg | neg | neg | neg | neg | neg | 1.73E+03 | neg | 2.55E+03 | 8.22E+02 |
| | swab cloaca 7 dpi | X | X | X | X | neg | 2.40E+03 | neg | neg | neg | neg | X | neg | | |

* cut- off $1.64*10^3$
**infectious virus recoverable

[S] sentinel animal

Organ panel included brain, lungs, duodenum, kidney, liver and heart of each duckling.
Separate swabs were taken once daily out of the choanal cleft and the cloaca from day 1 pi to day 7 or until the earlier individual date of death of the ducklings.

**Supplementary Table 3. Viral load of organs of surviving ducklings inoculated intramuscularly (IMPI) with different genotypes.**

**ct-value**

**genotype #8**

| animal | #1 | #2 | #3 | #4 | #5 |
|---|---|---|---|---|---|
| brain* | 27.3 | 27.42 | 25.56 | 28.03 | 30.29 |
| lungs | 37.4 | neg | 35.45 | 37.77 | neg |
| DD | neg | 37.4 | 37.44 | neg | neg |
| kidney | neg | neg | neg | neg | neg |
| liver | 36.57 | neg | neg | neg | neg |
| heart | 35.73 | neg | 36.25 | 35.96 | 36.4 |

* no infectious virus recoverable

**genotype #6**

| animal | #1 | #2 | #3 | #4 | #5 | #6 [S] |
|---|---|---|---|---|---|---|
| brain | 32.99 | 33.01 | 23.73 | 34.04 | 33.48 | 31.12 |
| kidney | neg | neg | neg | neg | neg | neg |

**genotype #9**

| animal | #1 | #2 | #3 | #4 | #5 | #6 [S] |
|---|---|---|---|---|---|---|
| brain | 41.07 | 38.19 | neg | 30.07 | 34.51 | neg |
| kidney | neg | neg | neg | neg | neg | neg |

**genotype #5**

| animal | #1 | #2 |
|---|---|---|
| brain | 29.41 | 30.61 |
| kidney | neg | neg |

VE/ml (Viral equivalents/ ml)

samples all derived from surviving animals at day 10 p.i.

**VE/mL**

**genotype #8**

| animal | #1 | #2 | #3 | #4 | #5 |
|---|---|---|---|---|---|
| brain* | $6.9x10^4$ | $6.4x10^4$ | $2.8x10^5$ | $4.2x10^4$ | $9.1x10^3$ |
| lungs | $7.2x10^1$ | neg | $2.7x10^2$ | $5.6x10^1$ | neg |
| DD | neg | $7.2x10^1$ | $7.0x10^1$ | neg | neg |
| kidney | neg | neg | neg | neg | neg |
| liver | 1.3x102 | neg | neg | neg | neg |
| heart | $2.2x10^2$ | neg | $1.6x10^2$ | $1.9x10^2$ | $1.4x10^2$ |

* no infectious virus recoverable

**genotype #6**

| animal | #1 | #2 | #3 | #4 | #5 | #6 [S] |
|---|---|---|---|---|---|---|
| brain | $6.9x10^4$ | $6.9x10^4$ | $7.3x10^7$ | $3.2x10^4$ | $4.8x10^4$ | $2.8x10^5$ |
| kidney | neg | neg | neg | neg | neg | neg |

**genotype #9**

| animal | #1 | #2 | #3 | #4 | #5 | #6 [S] |
|---|---|---|---|---|---|---|
| brain | $1.6x10^2$ | $1.4x10^3$ | neg | $6.2x10^5$ | $2.2x10^4$ | neg |
| kidney | neg | neg | neg | neg | neg | neg |

**genotype #5**

| animal | #1 | #2 |
|---|---|---|
| brain | $1.0x10^6$ | $4.1x10^5$ |
| kidney | neg | neg |

VE/ml (Viral equivalents/ ml)

samples all derived from surviving animals at day 10 p.i.

**Supplementary Table 4.1. Hemagglutinition inhibition assays of IMPI and O/N experiments with genotype #8 (DE 21-12 N1.4).**

| genotype | animal # | inoculation route | dpi | antigens | | | |
|---|---|---|---|---|---|---|---|
| | | | | *genotype 4 | *genotype 8 | **AR8444/2016 | **R2472/2014 |
| #8 | 1 | i.m. | 10 | | 0 | | |
| #8 | 2 | i.m. | 10 | | 0 | | |
| #8 | 3 | i.m. | 10 | | 3 | | |
| #8 | 4 | i.m. | 10 | | 0 | | |
| #8 | 5 | i.m. | 10 | | 0 | | |
| #8 | 390 | o.n. | 21 | 4 | 6 | 4 | 4 |
| #8 | 391 | o.n. | 21 | 6 | 8 | 6 | 6 |
| #8 | 393 | o.n. | 21 | 6 | 7 | 6 | 6 |
| #8 | 396 | o.n. | 21 | 6 | 7 | X | 6 |
| #8 | 397 | o.n. | 21 | 6 | 8 | 6 | 6 |
| #8 | 398 | o.n. | 21 | 6 | 7 | 6 | 6 |
| a- AR8444/16 #43 | | | | 6 | 7 | 8 | 8 |
| a- AR8444/16 #47 | | | | 7 | 9 | 8 | 8 |
| a- AR2472/14 #G27 | | | | 4 | 3 | 5 | 7 |
| a- AR2472/14 #G29 | | | | 4 | 5 | 5 | 8 |
| SPF- negative serum | | | | 0 | 0 | 0 | 0 |

Sampes derived from surviving animals at day 10 p.í or day 21 pi respectively

Two homologous (*) as well as two heterologous (**) antigens of 2016 and 2014 were used.
H5N8 A/Tufted duck/Germany/ AR8444/2016
H5N8 A/turkey/Germany/ R2472/2014

Positive control sera of 2016 and 2014 as well as negative control sera were used.

**Supplementary Table 4. Serological data of IMPI and O/N inoculated animals at the end of experiments.**

**IDVet ID screen Influenza A Antibody Competition Multi- species ELISA**

| genotype | animal # | inoculation route | dpi | S/N (%) | | **MW** |
|---|---|---|---|---|---|---|
| **#8** | 1 | i.m. | 10 | 24.28 | 28.29 | **26.28** |
| **#8** | 2 | i.m. | 10 | 7.45 | 7.24 | **7.35** |
| **#8** | 3 | i.m. | 10 | 9.73 | 9.31 | **9.52** |
| **#8** | 4 | i.m. | 10 | 21.80 | 20.42 | **21.11** |
| **#8** | 5 | i.m. | 10 | 11.45 | 13.94 | **12.69** |

**positive** **<45% S/N (%)**
**negative** **>50% S/N (%)**

**IDEXX Flockchek AI MultiS-Screen (NP)**

| genotype | animal # | inoculation route | dpi | S/N | | **MW** |
|---|---|---|---|---|---|---|
| **#6** | 1 | i.m. | 10 | 0.34 | 0.43 | **0.38** |
| **#6** | 2 | i.m. | 10 | 0.50 | 0.48 | **0.49** |
| **#6** | 3 | i.m. | 10 | 0.27 | 0.29 | **0.28** |
| **#6** | 4 | i.m. | 10 | 0.35 | 0.32 | **0.34** |
| **#6** | 5 | i.m. | 10 | 0.20 | 0.23 | **0.21** |
| **#6** | [S] | i.m. | 10 | 0.48 | 0.50 | **0.49** |
| **#9** | 1 | i.m. | 10 | 0.38 | 0.40 | **0.39** |
| **#9** | 2 | i.m. | 10 | 0.30 | 0.31 | **0.30** |
| **#9** | 3 | i.m. | 10 | 0.24 | 0.27 | **0.26** |
| **#9** | 4 | i.m. | 10 | 0.36 | 0.36 | **0.36** |
| **#9** | 5 | i.m. | 10 | 0.37 | 0.25 | **0.31** |
| **#9** | [S] | i.m. | 10 | 0.39 | 0.39 | **0.39** |
| **#5** | 1 | i.m. | 10 | 0.29 | 0.30 | **0.29** |
| **#5** | 2 | i.m. | 10 | 0.31 | 0.30 | **0.30** |
| | | | | | | |
| **#8** | 390 | o.n. | 21 | 0.46 | 0.40 | **0.43** |
| **#8** | 391 | o.n. | 21 | 0.22 | 0.31 | **0.26** |
| **#8** | 393 | o.n. | 21 | 0.31 | 0.26 | **0.28** |
| **#8** | 396 | o.n. | 21 | 0.26 | 0.31 | **0.28** |
| **#8** | 397 | o.n. | 21 | 0.29 | 0.22 | **0.26** |
| **#8** | 398 | o.n. | 21 | 0.36 | 0.45 | **0.40** |

**positive** **<0,5 S/N**
**negative** **>0,5 S/N**

Sera of ducklings surviving until day 10 pi or 21 dpi respectively.

Competitive ELISA kits for the detection of antibodies against the nucleoprotein of the Influenza A virus were used.

[green box] Mean values of double approach tested sera

**S/N** Signal to noise ratio

**Supplementary Table 5. Sequence information with virus name, country and accession number. We acknowledge the listed originating and submitting laboratories for sharing their data.**

<table>
<tr><th>virus name</th><th>country</th><th>accession</th><th>submitting lab</th><th>originating lab</th></tr>
<tr><td>A/guinea fowl/Germany-NW/AI01184/2020|EPI ISL 661312||A / H5N8</td><td>Germany</td><td>EPI1814671</td><td>Friedrich- Loeffler- Institut</td><td>Chemisches und Veterinäruntersuchungsamt Münsterland-Emscher-Lippe</td></tr>
<tr><td>A/tufted duck/Poland/MB061/2021(H5N5)|EPI ISL 1255051||A / H5N5</td><td>Poland</td><td>EPI1850515</td><td>National Veterinary Research Institut Poland, PIWet-PIB</td><td>National Veterinary Research Institut Poland, PIWet-PIB</td></tr>
<tr><td>A/chicken/Italy/IZSLT-122448 21VIR9218-1/2021|EPI ISL 7733644||A / H5N1</td><td>Italy</td><td>EPI1944474</td><td>Istituto Zooprofilattico Sperimentale Delle Venezie</td><td>Istituto Zooprofilattico Sperimentale del Lazio e della Toscana "M. Aleandri"</td></tr>
<tr><td>A/chicken/Wales/053969/2021|EPI ISL 9012618||A / H5N1</td><td>United Kingdom</td><td>EPI1963373</td><td>Animal and Plant Health Agency (APHA)</td><td>Animal and Plant Health Agency (APHA)</td></tr>
<tr><td>A/hen/Bulgaria/757-6 22VIR778-7/2021|EPI ISL 11007541||A / H5N1</td><td>Bulgaria</td><td>EPI1995169</td><td>Istituto Zooprofilattico Sperimentale delle Venezie</td><td>NDRVMI (National Diagnostic and Research Veterinary Medical Institute)</td></tr>
<tr><td>A/laying hen/Romania/10470 22VIR2749-5/2022|EPI ISL 11922819||A / H5N1</td><td>Romania</td><td>EPI2015014</td><td>Istituto Zooprofilattico Sperimentale delle Venezie</td><td>Institute for Diagnosis & Animal Health (IDAH)</td></tr>
<tr><td>A/chicken/Czech Republic/8028-1/2022|EPI ISL 13955204||A / H5N1</td><td>Czech Republic</td><td>EPI2098393</td><td>State Veterinary Institute Prague</td><td>State Veterinary Institute Prague</td></tr>
<tr><td>A/duck/Moscow/5881/2021|EPI ISL 14931847|OP133621|A / H3N2</td><td>Russian Federation</td><td>EPI2175869</td><td>-</td><td>-</td></tr>
<tr><td>A/mallard/Novosibirsk region/1894k/2019|EPI ISL 400274||A / H4N6</td><td>Russian Federation</td><td>EPI1638465</td><td>National Institute of Animal Health; Japan</td><td>Research Institute of Experimental and Clinical Medicine</td></tr>
<tr><td>A/mallard/Novosibirsk region/3541k/2020|EPI ISL 1241001||A / H12N5</td><td>Russian Federation</td><td>EPI1850481</td><td>National Institute of Animal Health; Japan</td><td>Research Institute of Experimental and Clinical Medicine</td></tr>
<tr><td>A/mandarin duck/Korea/WA585/2021|EPI ISL 6959592||A / H5N1</td><td>Korea</td><td>EPI1938210</td><td>Animal and Plant Quarantine Agency (APQA)</td><td>Animal and Plant Quarantine Agency (O-2144)</td></tr>
<tr><td>A/duck/Italy/21VIR8022-1/2021|EPI ISL 7975818||A / H5N3</td><td>Italy</td><td>EPI1946712</td><td>Istituto Zooprofilattico Sperimentale Delle Venezie</td><td>Istituto Zooprofilattico Sperimentale della Lombardia e dell'Emilia Romagna</td></tr>
<tr><td>A/teal/Italy/21VIR8923-73/2021|EPI ISL 7987343||A / H5N3</td><td>Italy</td><td>EPI1947346</td><td>Istituto Zooprofilattico Sperimentale Delle Venezie</td><td>Istituto Zooprofilattico Sperimentale Delle Venezie</td></tr>
<tr><td>A/swan/Slovenia/2049 22VIR777-3/2021|EPI ISL 11007530||A / H5N1</td><td>Slovenia</td><td>EPI1995081</td><td>Istituto Zooprofilattico Sperimentale Delle Venezie</td><td>University of Ljubljana</td></tr>
<tr><td>A/mandarin duck/Korea/H519/2021|EPI ISL 17606814||A / H5N1</td><td>Korea</td><td>EPI2540373</td><td>Animal and Plant Quarantine Agency (APQA)</td><td>Animal and Plant Quarantine Agency (APQA)</td></tr>
<tr><td>A/chicken/Korea/H57/2022|EPI ISL 17606841||A / H5N1</td><td>Korea</td><td>EPI2540589</td><td>Animal and Plant Quarantine Agency (APQA)</td><td>Animal and Plant Quarantine Agency (APQA)</td></tr>
<tr><td>A/chicken/Korea/H141/2022|EPI ISL 17606857||A / H5N1</td><td>Korea</td><td>EPI2540717</td><td>Animal and Plant Quarantine Agency (APQA)</td><td>Animal and Plant Quarantine Agency (APQA)</td></tr>
<tr><td>A/Anser anser/France/22P007390/2022|EPI ISL 18219237||A / H5N1</td><td>France</td><td>EPI2725871</td><td>ANSES Agence Nationale De Securite Sanitaire De L'alimentation</td><td>Anses (Ploufragan-Plouzané)</td></tr>
<tr><td>A/barnacle goose/Netherlands/2/2014|EPI ISL 267243|MF145805|A / H3N6</td><td>Netherlands</td><td>EPI1010642</td><td>-</td><td>-</td></tr>
<tr><td>A/mallard/Dagestan/1050/2018|EPI ISL 331289||A / H7N3</td><td>Russian Federation</td><td>EPI1319312</td><td>Research Institute of Experimental and Clinical Medicine</td><td>Research Institute of Experimental and Clinical Medicine</td></tr>
<tr><td>A/Eurasian-teal/Egypt/P2-29/2017|EPI ISL 341563||A / H6N2</td><td>Egypt</td><td>EPI1370641</td><td>Animal Health Research Institute</td><td>National Laboratory for Veterinary Quality Control on Poultry production- Animal Health Research Inistitute</td></tr>
<tr><td>A/chicken/Netherlands/17013178-006010/2017|EPI ISL 394155||A / H10N7</td><td>Netherlands</td><td>EPI1604934</td><td>Wageningen Bioveterinary Research</td><td>Wageningen Bioveterinary Research</td></tr>
<tr><td>A/environment/Netherlands/17013178/2017|EPI ISL 394156||A / H10N7</td><td>Netherlands</td><td>EPI1604942</td><td>Wageningen Bioveterinary Research</td><td>Wageningen Bioveterinary Research</td></tr>
<tr><td>A/Garganey/Omsk Region/22/2019|EPI ISL 400285||A / H3N8</td><td>Russian Federation</td><td>EPI1638553</td><td>National Institute of Animal Health; Japan</td><td>Research Institute of Experimental and Clinical Medicine</td></tr>
<tr><td>A/turkey/Germany-SH/AR495-L02996/2017|EPI ISL 436150||A / H5N5</td><td>Germany</td><td>EPI1737031</td><td>Friedrich-Loeffler-Institut</td><td>Landeslabor Schleswig-Holstein</td></tr>
<tr><td>A/turkey/Germany-SH/AR494-L03296/2017|EPI ISL 436696||A / H5N5</td><td>Germany</td><td>EPI1737418</td><td>Friedrich-Loeffler-Institut</td><td>Landeslabor Schleswig-Holstein</td></tr>
<tr><td>A/mallard/Germany-SH/AR1659-L03308/2017|EPI ISL 436708||A / H5N5</td><td>Germany</td><td>EPI1737515</td><td>Friedrich-Loeffler-Institut</td><td>Landeslabor Schleswig-Holstein</td></tr>
<tr><td>A/Anas platyrhynchos/Belgium/11958/2018|EPI ISL 502605|MT406944|A / H1N1</td><td>Belgium</td><td>EPI1774407</td><td>-</td><td>-</td></tr>
<tr><td>A/duck/Mongolia/447/2018|EPI ISL 697695|MW188626|A / H4N6</td><td>Mongolia</td><td>EPI1818097</td><td>-</td><td>-</td></tr>
<tr><td>A/mute swan/Netherlands/21038706-002/2021|EPI ISL 6761019||A / H5N1</td><td>Netherlands</td><td>EPI1930110</td><td>Wageningen Bioveterinary Research</td><td>Wageningen Bioveterinary Research</td></tr>
<tr><td>A/mute swan/Croatia/100/2021|EPI ISL 6507374||A / H5N1</td><td>Croatia</td><td>EPI1941512</td><td>Croatian Veterinary Institute</td><td>Croatian Veterinary Institute, Poultry Centre</td></tr>
<tr><td>A/swan/Romania/10986 22VIR2749-8/2022|EPI ISL 11922822||A / H5N1</td><td>Romania</td><td>EPI2015038</td><td>Istituto Zooprofilattico Sperimentale delle Venezie</td><td>Institute for Diagnosis & Animal Health (IDAH)</td></tr>
<tr><td>A/mallard/Poland/P074w5/2018|EPI ISL 17978924||A / H3N8</td><td>Poland</td><td>EPI2618249</td><td>National Veterinary Research Institute</td><td>National Veterinary Research Institut Poland, PIWet-PIB</td></tr>
<tr><td>A/goose/Netherlands/22000487-001/2022|EPI ISL 9111095||A / H5N1</td><td>Netherlands</td><td>EPI1965210</td><td>Wageningen Bioveterinary Research</td><td>Wageningen Bioveterinary Research</td></tr>
<tr><td>A/red fox/Netherlands/22004172-003/2022|EPI ISL 10915976||A / H5N1</td><td>Netherlands</td><td>EPI1994443</td><td>Wageningen Bioveterinary Research</td><td>Wageningen Bioveterinary Research</td></tr>
<tr><td>A/Eurasian Curlew/Netherlands/1/2022|EPI ISL 11112356||A / H5N1</td><td>Netherlands</td><td>EPI1995687</td><td>Erasmus Medical Center</td><td>Erasmus Medical Center</td></tr>
<tr><td>A/European Herring Gull/Netherlands/2/2022|EPI ISL 11112358||A / H5N1</td><td>Netherlands</td><td>EPI1995703</td><td>Erasmus Medical Center</td><td>Erasmus Medical Center</td></tr>
<tr><td>A/Barnacle Goose/Netherlands/17/2022|EPI ISL 11560236||A / H5N1</td><td>Netherlands</td><td>EPI2008066</td><td>Erasmus Medical Center</td><td>Erasmus Medical Center</td></tr>
<tr><td>A/Fox/Netherlands/EMC3/2022|EPI ISL 12069289||A / H5N1</td><td>Netherlands</td><td>EPI2018080</td><td>Erasmus Medical Center</td><td>Erasmus Medical Center</td></tr>
<tr><td>A/Falco peregrinus/Belgium/4055 0002/2022|EPI ISL 12210335||A / H5N1</td><td>Belgium</td><td>EPI2022934</td><td>Sciensano, Department of Animal Infectious Diseases</td><td>Sciensano - Animal Infectious Diseases</td></tr>
<tr><td>A/white stork/Netherlands/22009973-002/2022|EPI ISL 13300324||A / H5N1</td><td>Netherlands</td><td>EPI2068340</td><td>Wageningen Bioveterinary Research</td><td>Wageningen Bioveterinary Research</td></tr>
<tr><td>A/domestic duck/England/042266/2022|EPI ISL 13370921||A / H5N1</td><td>United Kingdom</td><td>EPI2072325</td><td>Animal and Plant Health Agency (APHA)</td><td>Animal and Plant Health Agency (APHA)</td></tr>
<tr><td>A/chicken/England/046274/2022|EPI ISL 13370923||A / H5N1</td><td>United Kingdom</td><td>EPI2072341</td><td>Animal and Plant Health Agency (APHA)</td><td>Animal and Plant Health Agency (APHA)</td></tr>
<tr><td>A/White-fronted goose/England/311038/2022|EPI ISL 13969429||A / H5N1</td><td>United Kingdom</td><td>EPI2100260</td><td>Animal and Plant Health Agency (APHA)</td><td>Animal and Plant Health Agency (APHA)</td></tr>
<tr><td>A/Branta leucopsis/Belgium/2606 0009/2022|EPI ISL 14392127||A / H5N1</td><td>Belgium</td><td>EPI2122914</td><td>Sciensano, Department of Animal Infectious Diseases</td><td>Sciensano - Animal Infectious Diseases</td></tr>
<tr><td>A/Mallard/Netherlands/18/2022|EPI ISL 15579543||A / H5N1</td><td>Netherlands</td><td>EPI2197854</td><td>Erasmus Medical Center</td><td>Erasmus Medical Center</td></tr>
<tr><td>A/turkey/England/125196/2022|EPI ISL 15585908||A / H5N1</td><td>United Kingdom</td><td>EPI2198625</td><td>Animal and Plant Health Agency (APHA)</td><td>Animal and Plant Health Agency (APHA)</td></tr>
<tr><td>A/turkey/England/138376/2022|EPI ISL 16384129||A / H5N1</td><td>United Kingdom</td><td>EPI2277838</td><td>Animal and Plant Health Agency (APHA)</td><td>Animal and Plant Health Agency (APHA)</td></tr>
<tr><td>A/Turkey/England/156090/2022|EPI ISL 16384148||A / H5N1</td><td>United Kingdom</td><td>EPI2277934</td><td>Animal and Plant Health Agency (APHA)</td><td>Animal and Plant Health Agency (APHA)</td></tr>
<tr><td>A/chicken/England/137719/2022|EPI ISL 16398192||A / H5N1</td><td>United Kingdom</td><td>EPI2281687</td><td>Animal and Plant Health Agency (APHA)</td><td>Animal and Plant Health Agency (APHA)</td></tr>
<tr><td>A/chicken/Czech Republic/185 orig/2023|EPI ISL 16613716||A / H5N1</td><td>Czech Republic</td><td>EPI2305270</td><td>State Veterinary Institute Prague</td><td>State Veterinary Institute Prague</td></tr>
<tr><td>A/Barnacle goose/Scotland/058494/2021|EPI ISL 13453561||A / H5N1</td><td>United Kingdom</td><td>EPI2075614</td><td>Animal and Plant Health Agency (APHA)</td><td>Animal and Plant Health Agency (APHA)</td></tr>
<tr><td>A/Eurasian wigeon/Germany-SH/AI05950/2021|EPI ISL 7748188||A / H5N1</td><td>Germany</td><td>EPI1945106</td><td>Friedrich-Loeffler-Institut</td><td>Landeslabor Schleswig-Holstein</td></tr>
<tr><td>A/white-tailed eagle/Germany-MV/AI05975/2021|EPI ISL 7753272||A / H5N1</td><td>Germany</td><td>EPI1945176</td><td>Friedrich-Loeffler-Institut</td><td>Landesamt für Landwirtschaft, Lebensmittelsicherheit und Fischerei (LALLF)</td></tr>
<tr><td>A/turkey/Germany-MV/AI06035/2021|EPI ISL 7753290||A / H5N1</td><td>Germany</td><td>EPI1945184</td><td>Friedrich-Loeffler-Institut</td><td>Landesamt für Landwirtschaft, Lebensmittelsicherheit und Fischerei (LALLF)</td></tr>
<tr><td>A/lesser white-fronted goose/Germany-MV/AI05973/2021|EPI ISL 7753309||A / H5N1</td><td>Germany</td><td>EPI1945192</td><td>Friedrich-Loeffler-Institut</td><td>Landesamt für Landwirtschaft, Lebensmittelsicherheit und Fischerei (LALLF)</td></tr>
<tr><td>A/white stork/Germany-MV/AI05979/2021|EPI ISL 7753329||A / H5N1</td><td>Germany</td><td>EPI1945200</td><td>Friedrich-Loeffler-Institut</td><td>Landesamt für Landwirtschaft, Lebensmittelsicherheit und Fischerei (LALLF)</td></tr>
<tr><td>A/barnacle goose/Germany-SH/AI06145/2021|EPI ISL 7753407||A / H5N1</td><td>Germany</td><td>EPI1945272</td><td>Friedrich-Loeffler-Institut</td><td>Landeslabor Schleswig-Holstein</td></tr>
<tr><td>A/chicken/Netherlands/21040808-001-005/2021|EPI ISL 7952117||A / H5N1</td><td>Netherlands</td><td>EPI1946308</td><td>Wageningen Bioveterinary Research</td><td>Wageningen Bioveterinary Research</td></tr>
<tr><td>A/greylag goose/Ireland/033062 22VIR1325-7/2021|EPI ISL 11259280||A / H5N1</td><td>Ireland</td><td>EPI1998111</td><td>Istituto Zooprofilattico Sperimentale delle Venezie</td><td>Central Veterinary Research Laboratory</td></tr>
<tr><td>A/goose/Ireland/3869 22VIR2064-2/2022|EPI ISL 11259284||A / H5N1</td><td>Ireland</td><td>EPI1998143</td><td>Istituto Zooprofilattico Sperimentale delle Venezie</td><td>Central Veterinary Research Laboratory</td></tr>
<tr><td>A/Great cormorant/Luxembourg/21243185/2021|EPI ISL 13026237||A / H5N1</td><td>Luxembourg</td><td>EPI2050804</td><td>Luxembourg Institute of Health</td><td>Laboratoire de Médecine vétérinaire de l'Etat</td></tr>
<tr><td>A/chicken/England/058797/2021|EPI ISL 13369759||A / H5N1</td><td>United Kingdom</td><td>EPI2071173</td><td>Animal and Plant Health Agency (APHA)</td><td>Animal and Plant Health Agency (APHA)</td></tr>
<tr><td>A/pink footed goose/Scotland/058488/2021|EPI ISL 13369760||A / H5N1</td><td>United Kingdom</td><td>EPI2071181</td><td>Animal and Plant Health Agency (APHA)</td><td>Animal and Plant Health Agency (APHA)</td></tr>
<tr><td>A/Kestrel/Scotland/058512/2021|EPI ISL 13369761||A / H5N1</td><td>United Kingdom</td><td>EPI2071189</td><td>Animal and Plant Health Agency (APHA)</td><td>Animal and Plant Health Agency (APHA)</td></tr>
<tr><td>A/barnacle goose/Scotland/062003/2021|EPI ISL 13369765||A / H5N1</td><td>United Kingdom</td><td>EPI2071221</td><td>Animal and Plant Health Agency (APHA)</td><td>Animal and Plant Health Agency (APHA)</td></tr>
<tr><td>A/peafowl/England/061723/2021|EPI ISL 13369775||A / H5N1</td><td>United Kingdom</td><td>EPI2071301</td><td>Animal and Plant Health Agency (APHA)</td><td>Animal and Plant Health Agency (APHA)</td></tr>
<tr><td>A/mute swan/Northern Ireland/17269/2021|EPI ISL 13370415||A / H5N1</td><td>United Kingdom</td><td>EPI2071413</td><td>Animal and Plant Health Agency (APHA)</td><td>Animal and Plant Health Agency (APHA)</td></tr>
<tr><td>A/common buzzard/Scotland/294149/2021|EPI ISL 13370418||A / H5N1</td><td>United Kingdom</td><td>EPI2071437</td><td>Animal and Plant Health Agency (APHA)</td><td>Animal and Plant Health Agency (APHA)</td></tr>
<tr><td>A/chicken/Scotland/064936/2021|EPI ISL 13370510||A / H5N1</td><td>United Kingdom</td><td>EPI2071509</td><td>Animal and Plant Health Agency (APHA)</td><td>Animal and Plant Health Agency (APHA)</td></tr>
<tr><td>A/pheasant/England/294628/2021|EPI ISL 13370560||A / H5N1</td><td>United Kingdom</td><td>EPI2071661</td><td>Animal and Plant Health Agency (APHA)</td><td>Animal and Plant Health Agency (APHA)</td></tr>
<tr><td>A/chicken/Northern Ireland/17903/2021|EPI ISL 13370564||A / H5N1</td><td>United Kingdom</td><td>EPI2071693</td><td>Animal and Plant Health Agency (APHA)</td><td>Animal and Plant Health Agency (APHA)</td></tr>
<tr><td>A/domestic duck/Northern Ireland/17961/2021|EPI ISL 13370568||A / H5N1</td><td>United Kingdom</td><td>EPI2071701</td><td>Animal and Plant Health Agency (APHA)</td><td>Animal and Plant Health Agency (APHA)</td></tr>
<tr><td>A/chicken/Scotland/070931/2021|EPI ISL 13370607||A / H5N1</td><td>United Kingdom</td><td>EPI2071781</td><td>Animal and Plant Health Agency (APHA)</td><td>Animal and Plant Health Agency (APHA)</td></tr>
<tr><td>A/common buzzard/England/245061/2021|EPI ISL 13370615||A / H5N1</td><td>United Kingdom</td><td>EPI2071845</td><td>Animal and Plant Health Agency (APHA)</td><td>Animal and Plant Health Agency (APHA)</td></tr>
<tr><td>A/sparrowhawk/England/245233/2021|EPI ISL 13370618||A / H5N1</td><td>United Kingdom</td><td>EPI2071869</td><td>Animal and Plant Health Agency (APHA)</td><td>Animal and Plant Health Agency (APHA)</td></tr>
<tr><td>A/mute swan/England/387121/2021|EPI ISL 13370619||A / H5N1</td><td>United Kingdom</td><td>EPI2071877</td><td>Animal and Plant Health Agency (APHA)</td><td>Animal and Plant Health Agency (APHA)</td></tr>
<tr><td>A/Goshawk/England/387180/2021|EPI ISL 13370630||A / H5N1</td><td>United Kingdom</td><td>EPI2071965</td><td>Animal and Plant Health Agency (APHA)</td><td>Animal and Plant Health Agency (APHA)</td></tr>
<tr><td>A/turkey/England/009687/2022|EPI ISL 13370700||A / H5N1</td><td>United Kingdom</td><td>EPI2072077</td><td>Animal and Plant Health Agency (APHA)</td><td>Animal and Plant Health Agency (APHA)</td></tr>
<tr><td>A/Barnacle goose/Scotland/058494/2021|EPI ISL 13453561||A / H5N1</td><td>United Kingdom</td><td>EPI2075614</td><td>Animal and Plant Health Agency (APHA)</td><td>Animal and Plant Health Agency (APHA)</td></tr>
<tr><td>A/Whooper swan/Scotland/072171/2021|EPI ISL 13486975||A / H5N1</td><td>United Kingdom</td><td>EPI2076402</td><td>Animal and Plant Health Agency (APHA)</td><td>Animal and Plant Health Agency (APHA)</td></tr>
<tr><td>A/Northern Gannet/Netherlands/5/2022|EPI ISL 15267026||A / H5N1</td><td>Netherlands</td><td>EPI2191466</td><td>Erasmus Medical Center</td><td>Erasmus Medical Center</td></tr>
<tr><td>|A/Eurasian wigeon/Germany-SH/AI06143/2021|EPI ISL 7753387||A / H5N1</td><td>Germany</td><td>EPI1945340</td><td>Friedrich-Loeffler-Institut</td><td>Landeslabor Schleswig-Holstein</td></tr>
<tr><td>A/chicken/Germany-MV/AI02141/2021|EPI ISL 5115621||A / H5N8</td><td>Germany</td><td>EPI1920479</td><td>Friedrich-Loeffler-Institut</td><td>Landesamt für Landwirtschaft, Lebensmittelsicherheit und Fischerei (LALLF)</td></tr>
<tr><td>A/turkey/Germany-NI/AI02303/2021|EPI ISL 5115955||A / H5N8</td><td>Germany</td><td>EPI1920496</td><td>Friedrich-Loeffler-Institut</td><td>Lebensmittel- und Veterinärinstitut Oldenburg - Standort Veterinärinstitut</td></tr>
<tr><td>A/chicken/Germany-BW/AI03205/2021|EPI ISL 5143820||A / H5N8</td><td>Germany</td><td>EPI1920944</td><td>Friedrich-Loeffler-Institut</td><td>CVUA Karlsruhe</td></tr>
<tr><td>A/chicken/Germany-NW/AI03148/2021|EPI ISL 5144341||A / H5N8</td><td>Germany</td><td>EPI1920969</td><td>Friedrich-Loeffler-Institut</td><td>Chemisches und Veterinäruntersuchungsamt Ostwestfalen-Lippe</td></tr>
<tr><td>A/domestic goose/Germany-NI/AI02980/2021|EPI ISL 5144725||A / H5N8</td><td>Germany</td><td>EPI1921017</td><td>Friedrich-Loeffler-Institut</td><td>Lebensmittel- und Veterinärinstitut Oldenburg - Standort Veterinärinstitut</td></tr>
<tr><td>A/swan/Germany-NI/AI05045/2021|EPI ISL 5145796||A / H5N8</td><td>Germany</td><td>EPI1921089</td><td>Friedrich-Loeffler-Institut</td><td>Lebensmittel- und Veterinärinstitut Oldenburg - Standort Veterinärinstitut</td></tr>
<tr><td>A/chicken/Germany-TH/AI03512/2021|EPI ISL 5145858||A / H5N8</td><td>Germany</td><td>EPI1921105</td><td>Friedrich-Loeffler-Institut</td><td>Thüringer Landesamt für Verbraucherschutz (TLAV)</td></tr>
<tr><td>A/Buteo buteo/Belgium/2606 0006/2022|EPI ISL 14391865||A / H5N1</td><td>Belgium</td><td>EPI2122907</td><td>Sciensano, Department of Animal Infectious Diseases</td><td>Sciensano - Animal Infectious Diseases</td></tr>
<tr><td>A/red knot/Germany-NI/AI00394/2020|EPI ISL 1206285||A / H5N3</td><td>Germany</td><td>EPI1850174</td><td>Friedrich-Loeffler-Institut</td><td>Lebensmittel- und Veterinärinstitut Oldenburg - Standort Veterinärinstitut</td></tr>
<tr><td>A/red knot/Germany-SH/AI03421/2020|EPI ISL 1205487||A / H5N3</td><td>Germany</td><td>EPI1850158</td><td>Friedrich-Loeffler-Institut</td><td>Landeslabor Schleswig-Holstein</td></tr>
<tr><td>A/red knot/France/21P003249/2021|EPI ISL 1259399||A / H5N3</td><td>France</td><td>EPI1850612</td><td>ANSES Agence Nationale De Securite Sanitaire De L'alimentation</td><td>Anses (Ploufragan-Plouzané)</td></tr>
<tr><td>A/red knot/Germany-SH/AI03424/2020|EPI ISL 1205489||A / H5N3</td><td>Germany</td><td>EPI1850166</td><td>Friedrich-Loeffler-Institut</td><td>Landeslabor Schleswig-Holstein</td></tr>
<tr><td>A/duck/France/22P020165/2022|EPI ISL 14933724||A / H5N1</td><td>France</td><td>EPI2176003</td><td>ANSES Agence Nationale De Securite Sanitaire De L'alimentation</td><td>Anses (Ploufragan-Plouzané)</td></tr>
<tr><td>A/Mallard/Netherlands/10/2022|EPI ISL 15364791||A / H5N2</td><td>Netherlands</td><td>EPI2193378</td><td>Erasmus Medical Center</td><td>Erasmus Medical Center</td></tr>
<tr><td>A/duck/Italy/21VIR8024-4/2021|EPI ISL 7987336||A / H5N3</td><td>Italy</td><td>EPI1947293</td><td>Istituto Zooprofilattico Sperimentale Delle Venezie</td><td>Istituto Zooprofilattico Sperimentale Delle Venezie</td></tr>
<tr><td>A/Chicken/Sweden/SVA210117SZ0004/KN011326-IP3/2021|EPI ISL 943553||A / H5N5</td><td>Sweden</td><td>EPI1843408</td><td>National Veterinary Institute</td><td>National Veterinary Institute, SVA</td></tr>
<tr><td>A/red fox/Denmark/01679-1.01-2/2022|EPI ISL 17447412||A / H5N1</td><td>Denmark</td><td>EPI2576131</td><td>Statens Serum Institute</td><td>Statens Serum Institute</td></tr>
<tr><td>A/fox/Finland/2844 21VIR9619-1/2021|EPI ISL 15518268||A / H5N1</td><td>Finland</td><td>EPI2197214</td><td>Istituto Zooprofilattico Sperimentale Delle Venezie</td><td>Finnish Food Authority</td></tr>
<tr><td>A/pheasant/Finland/12309 21RS3535-4/2021|EPI ISL 15518264||A / H5N1</td><td>Finland</td><td>EPI2197182</td><td>Istituto Zooprofilattico Sperimentale Delle Venezie</td><td>Finnish Food Authority</td></tr>
<tr><td>A/chicken/Germany-BB/AI06242/2021|EPI ISL 11725988||A / H5N1</td><td>Germany</td><td>EPI2009957</td><td>Friedrich-Loeffler-Institut</td><td>Landeslabor Berlin-Brandenburg</td></tr>
<tr><td>A/black-headed gull/Germany-NI/AI05962/2021|EPI ISL 7733048||A / H5N1</td><td>Germany</td><td>EPI1944231</td><td>Friedrich-Loeffler-Institut</td><td>Lebensmittel- und Veterinärinstitut Oldenburg - Standort Veterinärinstitut</td></tr>
<tr><td>A/chicken/Netherlands/21038211-001/2021|EPI ISL 6761005||A / H5N1</td><td>Netherlands</td><td>EPI1930013</td><td>Wageningen Bioveterinary Research</td><td>Wageningen Bioveterinary Research</td></tr>
<tr><td>A/chicken/England/064131/2021|EPI ISL 13370421||A / H5N1</td><td>United Kingdom</td><td>EPI2071462</td><td>Animal and Plant Health Agency (APHA)</td><td>Animal and Plant Health Agency (APHA)</td></tr>
<tr><td>A/barnacle goose/Denmark/24342-1.02/2021-10-30|EPI ISL 11798569||A / H5N1</td><td>Denmark</td><td>EPI2011638</td><td>Statens Serumm Institute</td><td>Statens Serumm Institute</td></tr>
<tr><td>A/mute swan/Poland/MB490-L1/2021|EPI ISL 6937114||A / H5N1</td><td>Poland</td><td>EPI1937676</td><td>National Veterinary Research Institut Poland, PIWet-PIB</td><td>National Veterinary Research Institut Poland, PIWet-PIB</td></tr>
<tr><td>A/red knot/Netherlands/22000409-002/2022|EPI ISL 9616212||A / H5N1</td><td>Netherlands</td><td>EPI1980938</td><td>Wageningen Bioveterinary Research</td><td>Wageningen Bioveterinary Research</td></tr>
<tr><td>A/chicken/Germany-NW/AI03154/2021|EPI ISL 5144407||A / H5N8</td><td>Germany</td><td>EPI1920984</td><td>Friedrich-Loeffler-Institut</td><td>Chemisches und Veterinäruntersuchungsamt Ostwestfalen-Lippe</td></tr>
<tr><td>A/turkey/Germany-NI/AI02615/2021|EPI ISL 5123589||A / H5N8</td><td>Germany</td><td>EPI1920616</td><td>Friedrich-Loeffler-Institut</td><td>Lebensmittel- und Veterinärinstitut Oldenburg - Standort Veterinärinstitut</td></tr>
<tr><td>A/mute swan/Netherlands/20015931-001/2020|EPI ISL 591075||A / H5N8</td><td>Netherlands</td><td>EPI1806857</td><td>Wageningen Bioveterinary Research</td><td>Wageningen Bioveterinary Research</td></tr>
<tr><td>A/mallard/Georgia/DT-23072/2020|EPI ISL 5863359||A / H5N8</td><td>Georgia</td><td>EPI1924195</td><td>Royal Veterinary College</td><td>Erasmus Medical Center</td></tr>
<tr><td>A/domestic goose/Germany-SH/AI02884/2020|EPI ISL 5061890</td><td>Germany</td><td>EPI1919701</td><td>Friedrich-Loeffler-Institut</td><td>Landeslabor Schleswig-Holstein</td></tr>
<tr><td>A/red knot/Germany-SH/AI03421/2020|EPI ISL 1205487</td><td>Germany</td><td>EPI1850159</td><td>Friedrich-Loeffler-Institut</td><td>Landeslabor Schleswig-Holstein</td></tr>
<tr><td>A/barnacle goose/Germany-NI/AI01605/2021|EPI ISL 5099456</td><td>Germany</td><td>EPI1920284</td><td>Friedrich-Loeffler-Institut</td><td>Lebensmittel- und Veterinärinstitut Oldenburg - Standort Veterinärinstitut</td></tr>
<tr><td>A/barnacle goose/Denmark/14537-1/2020|EPI ISL 984684||A / H5N8</td><td>Denmark</td><td>EPI1883003</td><td>Statens Serumm Institute</td><td>Statens Serumm Institute</td></tr>
<tr><td>A/sanderling/Netherlands/22002272-001/2021|EPI ISL 10724257||A / H5N1</td><td>Netherlands</td><td>EPI1990430</td><td>Wageningen Bioveterinary Research</td><td>Wageningen Bioveterinary Research</td></tr>
<tr><td>A/red knot/Netherlands/21041030-008/2021|EPI ISL 8650949||A / H5N1</td><td>Netherlands</td><td>EPI1956654</td><td>Wageningen Bioveterinary Research</td><td>Wageningen Bioveterinary Research</td></tr>
<tr><td>A/turkey/Germany-MV/AI06035/2021|EPI ISL 7753290||A / H5N1</td><td>Germany</td><td>EPI1945185</td><td>Friedrich-Loeffler-Institut</td><td>Landesamt für Landwirtschaft, Lebensmittelsicherheit und Fischerei (LALLF)</td></tr>
<tr><td>A/white-tailed eagle/Germany-MV/AI05975/2021|EPI ISL 7753272||A / H5N1</td><td>Germany</td><td>EPI1945177</td><td>Friedrich-Loeffler-Institut</td><td>Landesamt für Landwirtschaft, Lebensmittelsicherheit und Fischerei (LALLF)</td></tr>
<tr><td>A/Eurasian wigeon/Germany-SH/AI05950/2021|EPI ISL 7748188||A / H5N1</td><td>Germany</td><td>EPI1945104</td><td>Friedrich-Loeffler-Institut</td><td>Landeslabor Schleswig-Holstein</td></tr>
<tr><td>A/long-eared owl/Germany-NI/AI09037/2021|EPI ISL 10347326||A / H5N1</td><td>Germany</td><td>EPI1987728</td><td>Friedrich-Loeffler-Institut</td><td>Lebensmittel- und Veterinärinstitut Oldenburg - Standort Veterinärinstitut</td></tr>
<tr><td>A/chicken/Czech Republic/63/2022|EPI ISL 11327090||A / H5N1</td><td>Czech Republic</td><td>EPI1999375</td><td>State Veterinary Institute Prague</td><td>State Veterinary Institute Prague</td></tr>
<tr><td>A/Withe-tiled eagle/Estonia/TA2124126-1 21VIR10433-11/2021|EPI ISL 7778754||A / H5N1</td><td>Estonia</td><td>EPI1945371</td><td>Istituto Zooprofilattico Sperimentale Delle Venezie</td><td>Estonian Veterinary and Food Laboratory</td></tr>
<tr><td>A/domestic duck/Poland/H1942-N/2021|EPI ISL 6934175||A / H5N1</td><td>Poland</td><td>EPI1937660</td><td>National Veterinary Research Institut Poland, PIWet-PIB</td><td>National Veterinary Research Institut Poland, PIWet-PIB</td></tr>
<tr><td>A/chicken/Netherlands/21038165-006010/2021|EPI ISL 6101848||A / H5N1</td><td>Netherlands</td><td>EPI1925530</td><td>Wageningen Bioveterinary Research</td><td>Wageningen Bioveterinary Research</td></tr>
<tr><td>A/chicken/Poland/H157 22VIR2515-3/2022|EPI ISL 11922809||A / H5N1</td><td>Poland</td><td>EPI2014935</td><td>National Veterinary Research Institut Poland, PIWet-PIB</td><td>National Veterinary Research Institut Poland, PIWet-PIB</td></tr>
<tr><td>A/greylag goose/Germany-SH/AI06144/2021|EPI ISL 7753406||A / H5N1</td><td>Germany</td><td>EPI1945265</td><td>Friedrich-Loeffler-Institut</td><td>Landeslabor Schleswig-Holstein</td></tr>
<tr><td>A/Eurasian teal/Germany-BY/AI05977/2021|EPI ISL 7753367||A / H5N1</td><td>Germany</td><td>EPI1945217</td><td>Friedrich-Loeffler-Institut</td><td>Bayrisches Landesamt für Gesundheit und Lebensmittelsicherheit (LGL)</td></tr>
<tr><td>A/mallard duck/England/388009/2022|EPI ISL 13370702||A / H5N1</td><td>United Kingdom</td><td>EPI2072094</td><td>Animal and Plant Health Agency (APHA)</td><td>Animal and Plant Health Agency (APHA)</td></tr>
<tr><td>A/chicken/England/118935/2022|EPI ISL 15585857||A / H5N1</td><td>United Kingdom</td><td>EPI2198330</td><td>Animal and Plant Health Agency (APHA)</td><td>Animal and Plant Health Agency (APHA)</td></tr>
<tr><td>A/barnacle goose/Netherlands/22002511-001/2022|EPI ISL 10454886||A / H5N1</td><td>Netherlands</td><td>EPI1988043</td><td>Wageningen Bioveterinary Research</td><td>Wageningen Bioveterinary Research</td></tr>
<tr><td>A/Oystercatcher/Netherlands/1/2022|EPI ISL 9261743||A / H5N1</td><td>Netherlands</td><td>EPI1966968</td><td>Wageningen Bioveterinary Research</td><td>Wageningen Bioveterinary Research</td></tr>
<tr><td>A/sandwich tern/Denmark/05712-1.02/2022|EPI ISL 16113225||A / H5N1</td><td>Denmark</td><td>EPI2244200</td><td>Statens Serum Institute</td><td>Statens Serum Institute</td></tr>
<tr><td>A/sandwich tern/Denmark/05802-1.01 2022|EPI ISL 16113229||A / H5N1</td><td>Denmark</td><td>EPI2244192</td><td>Statens Serum Institute</td><td>Statens Serum Institute</td></tr>
<tr><td>A/great cormorant/Germany-MV/AI02666/2022|EPI ISL 14571448||A / H5N1</td><td>Germany</td><td>EPI2131903</td><td>Friedrich-Loeffler-Institut</td><td>Landesamt für Landwirtschaft, Lebensmittelsicherheit und Fischerei (LALLF)</td></tr>
<tr><td>A/Fox/Netherlands/EMC1/2022|EPI ISL 12066188||A / H5N1</td><td>Netherlands</td><td>EPI2018066</td><td>Wageningen Bioveterinary Research</td><td>Wageningen Bioveterinary Research</td></tr>
<tr><td>A/black-headed gull/Poland/MB139/2022|EPI ISL 14917999||A / H5N1</td><td>Poland</td><td>EPI2167235</td><td>National Veterinary Research Institut Poland, PIWet-PIB</td><td>National Veterinary Research Institut Poland, PIWet-PIB</td></tr>
<tr><td>A/Fox/Netherlands/EMC3/2022|EPI ISL 12069289||A / H5N1</td><td>Netherlands</td><td>EPI2018081</td><td>Wageningen Bioveterinary Research</td><td>Wageningen Bioveterinary Research</td></tr>
<tr><td>A/red fox/Netherlands/22004172-003/2022|EPI ISL 10915976||A / H5N1</td><td>Netherlands</td><td>EPI1994444</td><td>Wageningen Bioveterinary Research</td><td>Wageningen Bioveterinary Research</td></tr>
<tr><td>A/chicken/Czech Republic/22968 orig/2022|EPI ISL 16613718||A / H5N1</td><td>Czech Republic</td><td>EPI2305287</td><td>State Veterinary Institute Prague</td><td>State Veterinary Institute Prague</td></tr>
<tr><td>A/Eurasian wigeon/Germany-SH/AI06143/2021|EPI ISL 7753387||A / H5N1</td><td>Germany</td><td>EPI1945339</td><td>Friedrich-Loeffler-Institut</td><td>Landeslabor Schleswig-Holstein</td></tr>
<tr><td>A/domestic duck/England/070195/2021|EPI ISL 13370572||A / H5N1</td><td>United Kingdom</td><td>EPI2071734</td><td>Animal and Plant Health Agency (APHA)</td><td>Animal and Plant Health Agency (APHA)</td></tr>
<tr><td>A/greylag goose/Germany-NI/AR11353-L02142/2016|EPI ISL 262056||A / H5N5</td><td>Germany</td><td>EPI990776</td><td>Friedrich-Loeffler-Institut</td><td>Lebensmittel- und Veterinärinstitut Oldenburg - Standort Veterinärinstitut</td></tr>
</table>

**Supplementary Table 6.1. Statistical analysis of swab samples derived from oculo- nasally inoculated ducklings with genotype #4 and #8.**

Statistical significance was determined by applying Kruskal-Wallis One way ANOVA// Tukey test// Mann- Whitney U test when appropriate using GraphPad Prism version 8.0.1. as well as SigmaPlot version 11.

**swabs choana**

| | Mean rank diff, | Significant? | Summary | Adjusted P Value |
|---|---|---|---|---|
| #4 day 1 vs. #4 day 2 | -9.94 | No | ns | >0,9999 |
| #4 day 1 vs. #4 day 3 | 0.139 | No | ns | >0,9999 |
| #4 day 1 vs. #4 day 4 | -18.8 | No | ns | >0,9999 |
| #4 day 1 vs. #4 day 5 | -12.4 | No | ns | >0,9999 |
| *#4 day 1 vs. #8 day 1* | *40.39* | *No* | *ns* | *0.2075* |
| #4 day 1 vs. #8 day 2 | 31.56 | No | ns | >0,9999 |
| #4 day 1 vs. #8 day 3 | 8.597 | No | ns | >0,9999 |
| #4 day 1 vs. #8 day 4 | 10.85 | No | ns | >0,9999 |
| #4 day 1 vs. #8 day 5 | 30.69 | No | ns | >0,9999 |
| #4 day 1 vs. #8 day 6 | 29.81 | No | ns | >0,9999 |
| **#4 day 1 vs. #8 day 7** | **58.81** | **Yes** | ***** | **0.0491** |
| #4 day 2 vs. #4 day 3 | 10.08 | No | ns | >0,9999 |
| #4 day 2 vs. #4 day 4 | -8.83 | No | ns | >0,9999 |
| #4 day 2 vs. #4 day 5 | -2.5 | No | ns | >0,9999 |
| **#4 day 2 vs. #8 day 1** | **50.33** | **Yes** | ****** | **0.0055** |
| *#4 day 2 vs. #8 day 2* | *41.5* | *No* | *ns* | *0.0551* |
| #4 day 2 vs. #8 day 3 | 18.54 | No | ns | >0,9999 |
| #4 day 2 vs. #8 day 4 | 20.79 | No | ns | >0,9999 |
| #4 day 2 vs. #8 day 5 | 40.64 | No | ns | 0.0523 |
| #4 day 2 vs. #8 day 6 | 39.75 | No | ns | >0,9999 |
| **#4 day 2 vs. #8 day 7** | **68.75** | **Yes** | ****** | **0.0027** |
| #4 day 3 vs. #4 day 4 | -18.9 | No | ns | >0,9999 |
| #4 dav 3 vs. #4 dav 5 | -12.6 | No | ns | >0.9999 |
| #4 day 3 vs. #8 day 1 | 40.25 | No | ns | 0.1091 |
| #4 day 3 vs. #8 day 2 | 31.42 | No | ns | 0.7547 |
| *#4 day 3 vs. #8 day 3* | *8.458* | *No* | *ns* | *>0,9999* |
| #4 day 3 vs. #8 day 4 | 10.71 | No | ns | >0,9999 |
| #4 day 3 vs. #8 day 5 | 30.55 | No | ns | 0.7681 |
| #4 day 3 vs. #8 day 6 | 29.67 | No | ns | >0,9999 |
| **#4 day 3 vs. #8 day 7** | **58.67** | **Yes** | ***** | **0.0304** |
| #4 day 4 vs. #4 day 5 | 6.333 | No | ns | >0,9999 |
| #4 day 4 vs. #8 day 1 | 59.17 | No | ns | 0.1465 |
| #4 day 4 vs. #8 day 2 | 50.33 | No | ns | 0.5543 |
| #4 day 4 vs. #8 day 3 | 27.38 | No | ns | >0,9999 |
| *#4 day 4 vs. #8 day 4* | *29.63* | *No* | *ns* | *>0,9999* |
| #4 day 4 vs. #8 day 5 | 49.47 | No | ns | 0.5838 |
| #4 day 4 vs. #8 day 6 | 48.58 | No | ns | >0,9999 |
| #4 day 4 vs. #8 day 7 | 77.58 | Yes | * | 0.0305 |
| #4 day 5 vs. #8 day 1 | 52.83 | No | ns | >0,9999 |
| #4 day 5 vs. #8 day 2 | 44 | No | ns | >0,9999 |
| #4 day 5 vs. #8 day 3 | 21.04 | No | ns | >0,9999 |
| #4 day 5 vs. #8 day 4 | 23.29 | No | ns | >0,9999 |
| *#4 day 5 vs. #8 day 5* | *43.14* | *No* | *ns* | *>0,9999* |
| #4 day 5 vs. #8 day 6 | 42.25 | No | ns | >0,9999 |
| #4 day 5 vs. #8 day 7 | 71.25 | No | ns | 0.3016 |
| #8 day 1 vs. #8 day 2 | -8.83 | No | ns | >0,9999 |
| #8 day 1 vs. #8 day 3 | -31.8 | No | ns | 0.8547 |
| #8 day 1 vs. #8 day 4 | -29.5 | No | ns | >0,9999 |
| #8 day 1 vs. #8 day 5 | -9.7 | No | ns | >0,9999 |
| #8 day 1 vs. #8 day 6 | -10.6 | No | ns | >0,9999 |
| #8 day 1 vs. #8 day 7 | 18.42 | No | ns | >0,9999 |
| #8 day 2 vs. #8 day 3 | -23 | No | ns | >0,9999 |
| #8 day 2 vs. #8 day 4 | -20.7 | No | ns | >0,9999 |
| #8 day 2 vs. #8 day 5 | -0.86 | No | ns | >0,9999 |
| #8 day 2 vs. #8 day 6 | -1.75 | No | ns | >0,9999 |
| #8 day 2 vs. #8 day 7 | 27.25 | No | ns | >0,9999 |
| #8 day 3 vs. #8 day 4 | 2.25 | No | ns | >0,9999 |
| #8 day 3 vs. #8 day 5 | 22.09 | No | ns | >0,9999 |
| #8 day 3 vs. #8 day 6 | 21.21 | No | ns | >0,9999 |
| #8 day 3 vs. #8 day 7 | 50.21 | No | ns | 0.1796 |
| #8 day 4 vs. #8 day 5 | 19.84 | No | ns | >0,9999 |
| #8 day 4 vs. #8 day 6 | 18.96 | No | ns | >0,9999 |
| #8 day 4 vs. #8 day 7 | 47.96 | No | ns | 0.2768 |
| #8 day 5 vs. #8 day 6 | -0.89 | No | ns | >0,9999 |
| #8 day 5 vs. #8 day 7 | 28.11 | No | ns | >0,9999 |
| #8 day 6 vs. #8 day 7 | 29 | No | ns | >0,9999 |

**swabs cloaca**

| | Mean rank diff, | Significant? | Summary | Adjusted P Value |
|---|---|---|---|---|
| day 1 #4 vs. day 2 #4 | 4.533 | No | ns | >0,9999 |
| day 1 #4 vs. day 3 #4 | -9.97 | No | ns | >0,9999 |
| day 1 #4 vs. day 4 #4 | -0.38 | No | ns | >0,9999 |
| day 1 #4 vs. day 5 #4 | -12.3 | No | ns | >0,9999 |
| day 1 #4 vs. day 1 #8 | 31.2 | No | ns | >0,9999 |
| day 1 #4 vs. day 2 #8 | 24.05 | No | ns | >0,9999 |
| day 1 #4 vs. day 3 #8 | 24.17 | No | ns | 0.7883 |
| day 1 #4 vs. day 4 #8 | 32.38 | No | ns | 0.1117 |
| day 1 #4 vs. day 5 #8 | 40.45 | No | ns | 0.0717 |
| day 1 #4 vs. day 6 #8 | 32.95 | No | ns | >0,9999 |
| day 1 #4 vs. day 7 #8 | 33.28 | No | ns | >0,9999 |
| day 2 #4 vs. day 3 #4 | -14.5 | No | ns | >0,9999 |
| day 2 #4 vs. day 4 #4 | -4.92 | No | ns | >0,9999 |
| day 2 #4 vs. day 5 #4 | -16.8 | No | ns | >0,9999 |
| day 2 #4 vs. day 1 #8 | 26.67 | No | ns | >0,9999 |
| day 2 #4 vs. day 2 #8 | 19.52 | No | ns | >0,9999 |
| day 2 #4 vs. day 3 #8 | 19.64 | No | ns | >0,9999 |
| day 2 #4 vs. day 4 #8 | 27.85 | No | ns | 0.3398 |
| day 2 #4 vs. day 5 #8 | 35.92 | No | ns | 0.1949 |
| day 2 #4 vs. day 6 #8 | 28.42 | No | ns | >0,9999 |
| day 2 #4 vs. day 7 #8 | 28.75 | No | ns | >0,9999 |
| day 3 #4 vs. day 4 #4 | 9.583 | No | ns | >0,9999 |
| dav 3 #4 vs. dav 5 #4 | -2.33 | No | ns | >0.9999 |
| day 3 #4 vs. day 1 #8 | 41.17 | No | ns | 0.6607 |
| day 3 #4 vs. day 2 #8 | 34.02 | No | ns | 0.1492 |
| **day 3 #4 vs. day 3 #8** | **34.14** | **Yes** | ***** | **0.0143** |
| **day 3 #4 vs. day 4 #8** | **42.35** | **Yes** | ****** | **0.0014** |
| **day 3 #4 vs. day 5 #8** | **50.42** | **Yes** | ****** | **0.002** |
| day 3 #4 vs. day 6 #8 | 42.92 | No | ns | 0.0983 |
| day 3 #4 vs. day 7 #8 | 43.25 | No | ns | 0.0902 |
| day 4 #4 vs. day 5 #4 | -11.9 | No | ns | >0,9999 |
| day 4 #4 vs. day 1 #8 | 31.58 | No | ns | >0,9999 |
| day 4 #4 vs. day 2 #8 | 24.43 | No | ns | >0,9999 |
| day 4 #4 vs. day 3 #8 | 24.56 | No | ns | >0,9999 |
| day 4 #4 vs. day 4 #8 | 32.76 | No | ns | >0,9999 |
| day 4 #4 vs. day 5 #8 | 40.83 | No | ns | 0.7016 |
| day 4 #4 vs. day 6 #8 | 33.33 | No | ns | >0,9999 |
| day 4 #4 vs. day 7 #8 | 33.67 | No | ns | >0,9999 |
| day 5 #4 vs. day 1 #8 | 43.5 | No | ns | >0,9999 |
| day 5 #4 vs. day 2 #8 | 36.35 | No | ns | >0,9999 |
| day 5 #4 vs. day 3 #8 | 36.47 | No | ns | >0,9999 |
| day 5 #4 vs. day 4 #8 | 44.68 | No | ns | 0.5117 |
| day 5 #4 vs. day 5 #8 | 52.75 | No | ns | 0.2382 |
| day 5 #4 vs. day 6 #8 | 45.25 | No | ns | >0,9999 |
| day 5 #4 vs. day 7 #8 | 45.58 | No | ns | >0,9999 |
| day 1 #8 vs. day 2 #8 | -7.15 | No | ns | >0,9999 |
| day 1 #8 vs. day 3 #8 | -7.03 | No | ns | >0,9999 |
| day 1 #8 vs. day 4 #8 | 1.179 | No | ns | >0,9999 |
| day 1 #8 vs. day 5 #8 | 9.25 | No | ns | >0,9999 |
| day 1 #8 vs. day 6 #8 | 1.75 | No | ns | >0,9999 |
| day 1 #8 vs. day 7 #8 | 2.083 | No | ns | >0,9999 |
| day 2 #8 vs. day 3 #8 | 0.122 | No | ns | >0,9999 |
| day 2 #8 vs. day 4 #8 | 8.329 | No | ns | >0,9999 |
| day 2 #8 vs. day 5 #8 | 16.4 | No | ns | >0,9999 |
| day 2 #8 vs. day 6 #8 | 8.9 | No | ns | >0,9999 |
| day 2 #8 vs. day 7 #8 | 9.233 | No | ns | >0,9999 |
| day 3 #8 vs. day 4 #8 | 8.206 | No | ns | >0,9999 |
| day 3 #8 vs. day 5 #8 | 16.28 | No | ns | >0,9999 |
| day 3 #8 vs. day 6 #8 | 8.778 | No | ns | >0,9999 |
| day 3 #8 vs. day 7 #8 | 9.111 | No | ns | >0,9999 |
| day 4 #8 vs. day 5 #8 | 8.071 | No | ns | >0,9999 |
| day 4 #8 vs. day 6 #8 | 0.571 | No | ns | >0,9999 |
| day 4 #8 vs. day 7 #8 | 0.905 | No | ns | >0,9999 |
| day 5 #8 vs. day 6 #8 | -7.5 | No | ns | >0,9999 |
| day 5 #8 vs. day 7 #8 | -7.17 | No | ns | >0,9999 |
| day 6 #8 vs. day 7 #8 | 0.333 | No | ns | >0,9999 |

| genotype #4:3 | choana 2 dpi | choana 3 dpi | choana 4 dpi | choana 5 dpi |
|---|---|---|---|---|
| animal 1 | N/A | 2711.51 | 1862.66 | |
| animal 2 | 1754.05 | 2534.29 | 712.29 | |
| animal 3 | 4733619.81 | 476196316.14 | 6203106.40 | |
| animal 4 | 18292482.77 | 619355981.13 | 3612247.66 | |
| animal 5 | 3203263.71 | 11059836.34 | 2635071.19 | |
| animal 6 | 34634150.30 | 451809304.48 | 8697191.85 | |
| animal 7 | 22573354.31 | 448428925.81 | 5102804.03 | |
| animal 8 | 1206676.88 | 202287549.61 | 9589130.31 | |
| animal 9 | 3917.66 | 707979.74 | 444431.30 | |
| animal 10 | 3531773.66 | 18019782.79 | 73840.36 | |

| genotype #8 | choana 2 dpi | choana 3 dpi | choana 4 dpi | choana 5 dpi |
|---|---|---|---|---|
| animal 1 | 2773.29 | neg | 1015255.39 | 1224937.96 |
| animal 2 | 287497.88 | 147352.18 | 21578775.12 | 2267573.23 |
| animal 3 | N/A | 130668.75 | 2971509.87 | 102754.79 |
| animal 4 | neg | 67476.91 | 2654935.07 | 96763.08 |
| animal 5 | 42677.65 | 56772.69 | 768949.64 | 63066.86 |
| animal 6 | 46006.17 | 354779.15 | 6737306.38 | 5754316.09 |
| animal 7 | 2069.17 | neg | 2217056.02 | 114146.82 |
| animal 8 | 2631.27 | N/A | 6010.85 | 5180026.63 |
| animal 9 | 2350.94 | 2819332.62 | 1252849.05 | 504953.06 |
| animal 10 | 5133.85 | 1948.51 | 96763.08 | 609241.95 |

| genotype #4:3 | cloaca 2 dpi | cloaca 3 dpi | cloaca 4 dpi | cloaca 5 dpi |
|---|---|---|---|---|
| animal 1 | 1689.40 | 5921.24 | 5832.97 | |
| animal 2 | 346.37 | 2534.29 | 6028238944.53 | |
| animal 3 | 146461148.90 | 10337005.24 | 10829077952.16 | |
| animal 4 | 154366585.35 | 7947681.08 | 14514162822.28 | |
| animal 5 | 101369291.29 | 264702.21 | 6401516523.45 | |
| animal 6 | 137920881.47 | 80315304.80 | 7899622069.97 | |
| animal 7 | 633468451.41 | 8007592.85 | 5386010366.42 | |
| animal 8 | 4110089971.25 | 1179794.41 | 13244206.32 | |
| animal 9 | 10508609978.32 | 33862567.41 | 108457695.97 | |
| animal 10 | 1851396.46 | 5983136441.93 | 57283361.03 | |

| genotype #8 | cloaca 2 dpi | cloaca 3 dpi | cloaca 4 dpi | cloaca 5 dpi |
|---|---|---|---|---|
| animal 1 | 100.00 | 100.00 | 12268.26 | 100.00 |
| animal 2 | 100.00 | 100.00 | 2572.65 | 20139.35 |
| animal 3 | 100.00 | 100.00 | 100.00 | 100.00 |
| animal 4 | 100.00 | 3127.38 | 22881.88 | 100.00 |
| animal 5 | 100.00 | 100.00 | 100.00 | 100.00 |
| animal 6 | 100.00 | 100.00 | 100.00 | 100.00 |
| animal 7 | 100.00 | 100.00 | 1992.91 | 5211.54 |
| animal 8 | 2496.51 | 6335.29 | 11816.13 | 1578.99 |
| animal 9 | 100.00 | 2180.85 | 100.00 | 100.00 |
| animal 10 | 100.00 | 100.00 | 4160.25 | 100.00 |

| groups | P value | Significantly different (P < 0.05)? | Mann-Whitney U | n coloumn A | n coloumn B | effect size $R_U$ (Ranks biserial correlation by Wendt et a |
|---|---|---|---|---|---|---|
| #4:3 choana 2 dpi vs. 3 dpi | 0.206 | no | 29 | 10 | 10 | 0.42 |
| #4:3 choana 2 dpi vs. 4 dpi | 0.54 | no | 37 | 10 | 10 | 0.26 |
| #4:3 choana 3 dpi vs. 4 dpi | 0.038 | yes | 22 | 10 | 10 | 0.56 |
| | | | | | | |
| #8 choana 2 dpi vs. 3 dpi | 0.072 | no | 12 | 10 | 10 | 0.76 |
| #8 choana 2 dpi vs. 4 dpi | 0.002 | yes | 4 | 10 | 10 | 0.92 |
| #8 choana 3 dpi vs. 4 dpi | 0.326 | no | 36.5 | 10 | 10 | 0.27 |
| | | | | | | |
| #4:3 cloaca 2 dpi vs. 3 dpi | 0.186 | no | 32 | 10 | 10 | 0.36 |
| #4:3 cloaca 2 dpi vs. 4 dpi | 0.186 | no | 32 | 10 | 10 | 0.36 |
| #4:3 cloaca 3 dpi vs. 4 dpi | 0.011 | yes | 16 | 10 | 10 | 0.68 |
| | | | | | | |
| #8 cloaca 2 dpi vs. 3 dpi | 0.28 | no | 39.5 | 10 | 10 | 0.21 |
| #8 cloaca 2 dpi vs. 4 dpi | 0.019 | yes | 23 | 10 | 10 | 0.54 |
| #8 cloaca 3 dpi vs. 4 dpi | 0.148 | no | 32 | 10 | 10 | 0.36 |
| | | | | | | |
| #4:3 choana 2 dpi vs. #8 choana 2 dpi | 0.755 | no | 21 | 10 | 10 | 0.58 |
| #4:3 choana 3 dpi vs. #8 choana 3 dpi | 0.97 | no | 49 | 10 | 10 | 0.02 |
| #4:3 choana 4 dpi vs. #8 choana 4 dpi | 1.97 | no | 49 | 10 | 10 | 0.02 |
| | | | | | | |
| #4:3 cloaca 2 dpi vs. #8 cloaca 2 dpi | 0.001 | yes | 2 | 10 | 10 | 0.96 |
| **#4:3 cloaca 3 dpi vs. #8 cloaca 3 dpi** | **0.004** | **yes** | **3** | **10** | **10** | **0.94** |
| #4:3 cloaca 4 dpi vs. #8 cloaca 4 dpi | 0.029 | yes | 23 | 10 | 10 | 0.54 |

**Supplementary Table 6. Statistical analysis of IMPI clinical scores. Statistical significance was determined by applying unpaired Mann- Whitney- U tests using GraphPad Prism version 8.0.1.**

Diffe

| genotypes | P value | P value summary | Significantly different (P < 0.05)? | Sum of ranks in columns | Mann-Whitney U | n coloumn A | Median of column B | n coloumn B | Difference: Actual | Difference: Hodges-Lehmann | effect size $R_U$ (Ranks biserial correlation by Wendt et al.1972 |
|---|---|---|---|---|---|---|---|---|---|---|---|
| #4:1 vs. #4:3 | >0.9999 | ns | No | 100 . 110 | 45 | 10 | 3.000. n=10 | 10 | 0 | 0 | 0.1 |
| #4:2 vs. #4:2 | 0.1409 | ns | No | 125 . 85 | 30 | 10 | 2.950. n=10 | 10 | -0.05 | 0 | 0.4 |
| #7 vs. #4:3 | 0.3034 | ns | No | 110.5 . 79.50 | 35 | 10 | 3.000. n=9 | 9 | 0 | 0 | 0.2 |
| #3 vs. #4:3 | 0.0174 | * | Yes | 135.5 . 74.50 | 20 | 10 | 2.900. n=10 | 10 | -0.1 | -0.1 | 0.6 |
| #5 vs. #4:3 | <0.0001 | **** | Yes | 155 . 55 | 0 | 10 | 1.900. n=10 | 10 | -1.1 | -1.1 | 1.0 |
| #6 vs. #4:3 | 0.0019 | ** | Yes | 143 . 67 | 12 | 10 | 1.250. n=10 | 10 | -1.75 | -1.7 | 0.8 |
| #9 vs. #4:3 | <0.0001 | **** | Yes | 155 . 55 | 0 | 10 | 1.350. n=10 | 10 | -1.65 | -1.6 | 1.0 |
| #8 vs. #4:3 | <0.0001 | **** | Yes | 145 . 45 | 0 | 10 | 0.7000. n=9 | 9 | -2.3 | -2.3 | 1.0 |
| **#2 vs. #4:3** | **0.0031** | ** | **Yes** | **138.5 . 71.50** | **17** | 10 | **2.750. n=10** | **10** | **-0.25** | **-0.2** | 0.7 |
| **#1 vs. #4:3** | **0.0004** | *** | **Yes** | **154 . 77** | **11** | 10 | **2.500. n=11** | **11** | **-0.5** | **-0.5** | 0.8 |
| #4:2 vs. #4:1 | 0.0325 | * | Yes | 130 . 80 | 25 | 10 | 2.950. n=10 | 10 | -0.05 | -0.05 | 0.5 |
| #7 vs. #4:1 | 0.0867 | ns | No | 115 . 75 | 30 | 10 | 3.000. n=9 | 9 | 0 | 0 | 0.3 |
| #3 vs. #4:1 | 0.0031 | ** | Yes | 140 . 70 | 15 | 10 | 2.900. n=10 | 10 | -0.1 | -0.1 | 0.7 |
| #5 vs. #4:1 | <0.0001 | **** | Yes | 155 . 55 | 0 | 10 | 1.900. n=10 | 10 | -1.1 | -1.1 | 1.0 |
| #6 vs. #4:1 | 0.0007 | *** | Yes | 145 . 65 | 10 | 10 | 1.250. n=10 | 10 | -1.75 | -1.75 | 0.8 |
| #9 vs. #4:1 | <0.0001 | **** | Yes | 155 . 55 | 0 | 10 | 1.350. n=10 | 10 | -1.65 | -1.65 | 1.0 |
| #8 vs. #4:1 | <0.0001 | **** | Yes | 145 . 45 | 0 | 10 | 0.7000. n=9 | 9 | -2.3 | -2.3 | 1.0 |
| #2 vs. #4:1 | 0.0031 | ** | Yes | 140 . 70 | 15 | 10 | 2.750. n=10 | 10 | -0.25 | -0.25 | 0.7 |
| #1 vs. #4:1 | 0.0002 | *** | Yes | 155 . 76 | 10 | 10 | 2.500. n=11 | 11 | -0.5 | -0.5 | 0.8 |
| #7 vs. #4:2 | 0.6499 | ns | No | 92.50 . 97.50 | 38 | 10 | 3.000. n=9 | 9 | 0.05 | 0 | 0.2 |
| #3 vs. #4:2 | 0.4499 | ns | No | 117.5 . 92.50 | 38 | 10 | 2.900. n=10 | 10 | -0.05 | 0 | 0.3 |
| #5 vs. #4:2 | <0.0001 | **** | Yes | 155 . 55 | 0 | 10 | 1.900. n=10 | 10 | -1.05 | -1.05 | 1.0 |
| #6 vs. #4:2 | 0.018 | * | Yes | 135 . 75 | 20 | 10 | 1.250. n=10 | 10 | -1.7 | -1.7 | 0.6 |
| #9 vs. #4:2 | <0.0001 | **** | Yes | 155 . 55 | 0 | 10 | 1.350. n=10 | 10 | -1.6 | -1.6 | 1.0 |
| #8 vs. #4:2 | <0.0001 | **** | Yes | 145 . 45 | 0 | 10 | 0.7000. n=9 | 9 | -2.25 | -2.25 | 1.0 |
| #2 vs. #4:2 | 0.0254 | * | Yes | 132.5 . 77.50 | 23 | 10 | 2.750. n=10 | 10 | -0.2 | -0.2 | 0.6 |
| #1 vs. #4:2 | 0.0026 | ** | Yes | 150 . 81 | 15 | 10 | 2.500. n=11 | 11 | -0.45 | -0.4 | 0.7 |
| #3 vs. #7 | 0.1462 | ns | No | 108 . 82 | 27 | 9 | 2.900. n=10 | 10 | -0.1 | -0.05 | 0.4 |
| #5 vs. #7 | <0.0001 | **** | Yes | 135 . 55 | 0 | 9 | 1.900. n=10 | 10 | -1.1 | -1.1 | 1.0 |
| #6 vs. #7 | 0.0093 | ** | Yes | 120 . 70 | 15 | 9 | 1.250. n=10 | 10 | -1.75 | -1.7 | 0.7 |
| #9 vs. #7 | <0.0001 | **** | Yes | 135 . 55 | 0 | 9 | 1.350. n=10 | 10 | -1.65 | -1.6 | 1.0 |
| #8 vs. #7 | <0.0001 | **** | Yes | 126 . 45 | 0 | 9 | 0.7000. n=9 | 9 | -2.3 | -2.3 | 1.0 |
| #2 vs. #7 | 0.0159 | * | Yes | 117 . 73 | 18 | 9 | 2.750. n=10 | 10 | -0.25 | -0.2 | 0.6 |
| #1 vs. #7 | 0.0024 | ** | Yes | 132 . 78 | 12 | 9 | 2.500. n=11 | 11 | -0.5 | -0.4 | 0.8 |
| #5 vs. #3 | <0.0001 | **** | Yes | 155 . 55 | 0 | 10 | 1.900. n=10 | 10 | -1 | -1 | 1.0 |
| #6 vs. #3 | 0.0516 | ns | No | 130 . 80 | 25 | 10 | 1.250. n=10 | 10 | -1.65 | -1.65 | 0.5 |
| #9 vs. #3 | <0.0001 | **** | Yes | 155 . 55 | 0 | 10 | 1.350. n=10 | 10 | -1.55 | -1.55 | 1.0 |
| #8 vs. #3 | <0.0001 | **** | Yes | 145 . 45 | 0 | 10 | 0.7000. n=9 | 9 | -2.2 | -2.2 | 1.0 |
| #2 vs. #3 | 0.0661 | ns | No | 128.5 . 81.50 | 27 | 10 | 2.750. n=10 | 10 | -0.15 | -0.2 | 0.5 |
| #1 vs. #3 | 0.0063 | ** | Yes | 147 . 84 | 18 | 10 | 2.500. n=11 | 11 | -0.4 | -0.4 | 0.7 |
| #6 vs. #5 | 0.9574 | ns | No | 104 . 106 | 49 | 10 | 1.250. n=10 | 10 | -0.65 | 0 | 0.0 |
| #9 vs. #5 | 0.22 | ns | No | 121.5 . 88.50 | 34 | 10 | 1.350. n=10 | 11 | -0.55 | -0.25 | 0.4 |
| #8 vs. #5 | 0.4306 | ns | No | 110 . 80 | 35 | 10 | 0.7000. n=9 | 9 | -1.2 | -0.2 | 0.2 |
| #2 vs. #5 | <0.0001 | **** | Yes | 55 . 155 | 0 | 10 | 2.750. n=10 | 10 | 0.85 | 0.9 | 1.0 |
| #1 vs. #5 | 0.002 | ** | Yes | 68.50 . 162.5 | 14 | 10 | 2.500. n=11 | 11 | 0.6 | 0.8 | 0.8 |
| #9 vs. #6 | >0.9999 | ns | No | 105 . 105 | 50 | 10 | 1.350. n=10 | 10 | 0.1 | 0 | 0.0 |
| #8 vs. #6 | 0.9507 | ns | No | 99 . 91 | 44 | 10 | 0.7000. n=9 | 9 | -0.55 | 0.05 | 0.0 |
| #2 vs. #6 | 0.1519 | ns | No | 86 . 124 | 31 | 10 | 2.750. n=10 | 10 | 1.5 | 1.45 | 0.4 |
| #1 vs. #6 | 0.3191 | ns | No | 95.50 . 135.5 | 41 | 10 | 2.500. n=11 | 11 | 1.25 | 1.2 | 0.3 |
| #8 vs. #9 | 0.8871 | ns | No | 98 . 92 | 43 | 10 | 0.7000. n=9 | 9 | -0.65 | 0 | 0.0 |
| #2 vs. #9 | <0.0001 | **** | Yes | 55 . 155 | 0 | 10 | 2.750. n=10 | 10 | 1.4 | 1.35 | 1.0 |
| #1 vs. #9 | 0.0001 | *** | Yes | 61 . 170 | 6 | 10 | 2.500. n=11 | 11 | 1.15 | 1.2 | 0.9 |
| #2 vs. #9 | <0.0001 | **** | Yes | 45 . 145 | 0 | 9 | 2.750. n=10 | 10 | 2.05 | 1.8 | 1.0 |
| #1 vs. #9 | 0.0012 | ** | Yes | 54.50 . 155.5 | 9.5 | 9 | 2.500. n=11 | 11 | 1.8 | 1.3 | 0.8 |
| #2 vs. #8 | | Exact | **** | Two-tailed | 145 | | 0,7000, n=9 | 2,750, n=10 | 2.05 | | |
| #1 vs. #8 | | Exact | ** | Two-tailed | 55.5 | | 0,7000, n=9 | 2,500, n=11 | 1.8 | | |
| #1 vs. #2 | 0.3315 | ns | No | 124 . 107 | 41 | 10 | 2.500. n=11 | 11 | -0.25 | -0.2 | 0.3 |
| #1 vs. #4:3 | 0.0004 | *** | Yes | 154 . 77 | 11 | 10 | 2.500. n=11 | 11 | -0.5 | -0.5 | 0.8 |
| #1 vs. #4:1 | 0.0002 | *** | Yes | 155 . 76 | 10 | 10 | 2.500. n=11 | 11 | -0.5 | -0.5 | 0.8 |
| #1 vs. #4:2 | 0.0026 | ** | Yes | 150 . 81 | 15 | 10 | 2.500. n=11 | 11 | -0.45 | -0.4 | 0.7 |
| #1 vs. #7 | 0.0024 | ** | Yes | 132 . 78 | 12 | 9 | 2.500. n=11 | 11 | -0.5 | -0.4 | 0.8 |
| #1 vs. #3 | 0.0063 | ** | Yes | 147 . 84 | 18 | 10 | 2.500. n=11 | 11 | -0.4 | -0.4 | 0.7 |
| #1 vs. #5 | 0.002 | ** | Yes | 68.50 . 162.5 | 14 | 10 | 2.500. n=11 | 11 | 0.6 | 0.8 | 0.8 |
| #1 vs. #6 | 0.3191 | ns | No | 95.50 . 135.5 | 41 | 10 | 2.500. n=11 | 11 | 1.25 | 1.2 | 0.3 |
| #1 vs. #9 | 0.0001 | *** | Yes | 61 . 170 | 6 | 10 | 2.500. n=11 | 11 | 1.15 | 1.2 | 0.9 |
| #1 vs. #8 | 0.0012 | ** | Yes | 54.50 . 155.5 | 9.5 | 9 | 2.500. n=11 | 11 | 1.8 | 1.3 | 0.8 |
| #1 vs. #2 | 0.3315 | ns | No | 124 . 107 | 41 | 10 | 2.500. n=11 | 11 | -0.25 | -0.2 | 0.3 |
| **#4:3 o.n. vs. #4:3 IMPI** | **<0,0001** | **** | **Yes** | **155 , 55** | **0** | **10** | **2,300, n=10** | **10** | **-0.7** | **-0.7** | 1.0 |
| **#8 o.n. vs. #8 IMPI** | **0.0383** | * | **Yes** | **115 , 75** | **20** | **9** | **0,000, n=10** | **10** | **-0.7** | **-0.6** | 0.6 |
| **#8 o.n. vs. #4:3 o.n.** | **<0,0001** | **** | **Yes** | **155 , 55** | **0** | **10** | **0,000, n=10** | **10** | **-2.3** | **-2.3** | 1.0 |

Graphpad version.

| | | #4:3 4286/22 | #4:1 1196/22 | #4:2 2407/22 | #7 951/22 | #3 1411/22 | #5 7088/22 | #6 1026/22 | #9 1073/22 | #8 616/22 | #2 5373/22 | #1 3705/22 |
|---|---|---|---|---|---|---|---|---|---|---|---|---|
| #4:3 | 4286/22 | | ns | ns | ns | * | **** | ** | **** | **** | ** | *** |
| #4:1 | 1196/22 | ns | | * | ns | ** | **** | *** | **** | **** | ** | *** |
| #4:2 | 2407/22 | ns | * | | ns | ns | **** | ** | **** | **** | * | ** |
| #7 | 951/22 | ns | ns | ns | | ns | **** | ** | **** | **** | * | ** |
| #3 | 1411/22 | * | ** | ns | ns | | **** | ns | **** | **** | ns | ** |
| #5 | 7088/22 | **** | **** | **** | **** | **** | | ns | ns | ns | **** | ** |
| #6 | 1026/22 | ** | *** | ** | ** | ns | ns | | ns | ns | ns | ns |
| #9 | 1073/22 | **** | **** | **** | **** | **** | ns | ns | | ns | **** | *** |
| #8 | 616/22 | **** | **** | **** | **** | **** | ns | ns | ns | | **** | ** |
| #2 | 5373/22 | ** | ** | * | * | ns | **** | * | **** | **** | | ns |
| #1 | 3705/22 | *** | *** | ** | ** | ** | ** | * | *** | ** | ns | |

| | | #4:3 4286/22 | #4:1 1196/22 | #4:2 2407/22 | #7 951/22 | #3 1411/22 | #5 7088/22 | #6 1026/22 | #9 1073/22 | #8 616/22 | #2 5373/22 | #1 3705/22 |
|---|---|---|---|---|---|---|---|---|---|---|---|---|
| #4:3 | 4286/22 | | | | | * | * | * | * | * | * | * |
| #4:1 | 1196/22 | | | * | | * | * | * | * | * | * | * |
| #4:2 | 2407/22 | | * | | | | * | * | * | * | * | * |
| #7 | 951/22 | | | | | | * | * | * | * | * | * |
| #3 | 1411/22 | * | * | | | | * | | * | * | | * |
| #5 | 7088/22 | * | * | * | * | * | | | | | * | * |
| #6 | 1026/22 | * | * | * | * | | | | | | | |
| #9 | 1073/22 | * | * | * | * | * | | | | | * | * |
| #8 | 616/22 | * | * | * | * | * | | | | | * | * |
| #2 | 5373/22 | * | * | * | * | | * | * | * | * | | |
| #1 | 3705/22 | * | * | * | * | * | * | * | * | * | | |

| #4:3 | day 1 | day 2 | day 3 | day 4 | day 5 | day 6 | day 7 | day 8 | day 9 | day 10 | evaluation |
|---|---|---|---|---|---|---|---|---|---|---|---|
| duckling # 1 | 3 | 3 | 3 | 3 | 3 | 3 | 3 | 3 | 3 | 3 | **3** |
| duckling # 2 | 3 | 3 | 3 | 3 | 3 | 3 | 3 | 3 | 3 | 3 | **3** |
| duckling # 3 | 3 | 3 | 3 | 3 | 3 | 3 | 3 | 3 | 3 | 3 | **3** |
| duckling # 4 | 3 | 3 | 3 | 3 | 3 | 3 | 3 | 3 | 3 | 3 | **3** |
| duckling # 5 | 3 | 3 | 3 | 3 | 3 | 3 | 3 | 3 | 3 | 3 | **3** |
| duckling # 6 | 3 | 3 | 3 | 3 | 3 | 3 | 3 | 3 | 3 | 3 | **3** |
| duckling # 7 | 3 | 3 | 3 | 3 | 3 | 3 | 3 | 3 | 3 | 3 | **3** |
| duckling # 8 | 3 | 3 | 3 | 3 | 3 | 3 | 3 | 3 | 3 | 3 | **3** |
| duckling # 9 | 3 | 3 | 3 | 3 | 3 | 3 | 3 | 3 | 3 | 3 | **3** |
| duckling # 10 | 2 | 3 | 3 | 3 | 3 | 3 | 3 | 3 | 3 | 3 | **2.9** |

| #4:1 | day 1 | day 2 | day 3 | day 4 | day 5 | day 6 | day 7 | day 8 | day 9 | day 10 | evaluation |
|---|---|---|---|---|---|---|---|---|---|---|---|
| duckling # 1 | 3 | 3 | 3 | 3 | 3 | 3 | 3 | 3 | 3 | 3 | **3** |
| duckling # 2 | 3 | 3 | 3 | 3 | 3 | 3 | 3 | 3 | 3 | 3 | **3** |
| duckling # 3 | 3 | 3 | 3 | 3 | 3 | 3 | 3 | 3 | 3 | 3 | **3** |
| duckling # 4 | 3 | 3 | 3 | 3 | 3 | 3 | 3 | 3 | 3 | 3 | **3** |
| duckling # 5 | 3 | 3 | 3 | 3 | 3 | 3 | 3 | 3 | 3 | 3 | **3** |
| duckling # 6 | 3 | 3 | 3 | 3 | 3 | 3 | 3 | 3 | 3 | 3 | **3** |
| duckling # 7 | 3 | 3 | 3 | 3 | 3 | 3 | 3 | 3 | 3 | 3 | **3** |
| duckling # 8 | 3 | 3 | 3 | 3 | 3 | 3 | 3 | 3 | 3 | 3 | **3** |
| duckling # 9 | 3 | 3 | 3 | 3 | 3 | 3 | 3 | 3 | 3 | 3 | **3** |
| duckling # 10 | 3 | 3 | 3 | 3 | 3 | 3 | 3 | 3 | 3 | 3 | **3** |

| #4:2 | day 1 | day 2 | day 3 | day 4 | day 5 | day 6 | day 7 | day 8 | day 9 | day 10 | evaluation |
|---|---|---|---|---|---|---|---|---|---|---|---|
| duckling # 1 | 3 | 3 | 3 | 3 | 3 | 3 | 3 | 3 | 3 | 3 | **3** |
| duckling # 2 | 3 | 3 | 3 | 3 | 3 | 3 | 3 | 3 | 3 | 3 | **3** |
| duckling # 3 | 3 | 3 | 3 | 3 | 3 | 3 | 3 | 3 | 3 | 3 | **3** |
| duckling # 4 | 3 | 3 | 3 | 3 | 3 | 3 | 3 | 3 | 3 | 3 | **3** |
| duckling # 5 | 3 | 3 | 3 | 3 | 3 | 3 | 3 | 3 | 3 | 3 | **3** |
| duckling # 6 | 2 | 3 | 3 | 3 | 3 | 3 | 3 | 3 | 3 | 3 | **2.9** |
| duckling # 7 | 2 | 3 | 3 | 3 | 3 | 3 | 3 | 3 | 3 | 3 | **2.9** |
| duckling # 8 | 2 | 3 | 3 | 3 | 3 | 3 | 3 | 3 | 3 | 3 | **2.9** |
| duckling # 9 | 2 | 3 | 3 | 3 | 3 | 3 | 3 | 3 | 3 | 3 | **2.9** |
| duckling # 10 | 2 | 3 | 3 | 3 | 3 | 3 | 3 | 3 | 3 | 3 | **2.9** |

| #7 | day 1 | day 2 | day 3 | day 4 | day 5 | day 6 | day 7 | day 8 | day 9 | day 10 | evaluation |
|---|---|---|---|---|---|---|---|---|---|---|---|
| duckling # 1 | 3 | 3 | 3 | 3 | 3 | 3 | 3 | 3 | 3 | 3 | **3** |
| duckling # 2 | 3 | 3 | 3 | 3 | 3 | 3 | 3 | 3 | 3 | 3 | **3** |
| duckling # 3 | 3 | 3 | 3 | 3 | 3 | 3 | 3 | 3 | 3 | 3 | **3** |
| duckling # 4 | 3 | 3 | 3 | 3 | 3 | 3 | 3 | 3 | 3 | 3 | **3** |
| duckling # 5 | 3 | 3 | 3 | 3 | 3 | 3 | 3 | 3 | 3 | 3 | **3** |
| duckling # 6 | 3 | 3 | 3 | 3 | 3 | 3 | 3 | 3 | 3 | 3 | **3** |
| duckling # 7 | 2 | 3 | 3 | 3 | 3 | 3 | 3 | 3 | 3 | 3 | **2.9** |
| duckling # 8 | 2 | 3 | 3 | 3 | 3 | 3 | 3 | 3 | 3 | 3 | **2.9** |
| duckling # 9 | 2 | 3 | 3 | 3 | 3 | 3 | 3 | 3 | 3 | 3 | **2.9** |
| duckling # 10 | 2 | 3 | 3 | 3 | 3 | 3 | 3 | 3 | 3 | 3 | **X** |

| #3 | day 1 | day 2 | day 3 | day 4 | day 5 | day 6 | day 7 | day 8 | day 9 | day 10 | evaluation |
|---|---|---|---|---|---|---|---|---|---|---|---|
| duckling # 1 | 3 | 3 | 3 | 3 | 3 | 3 | 3 | 3 | 3 | 3 | **3** |
| duckling # 2 | 3 | 3 | 3 | 3 | 3 | 3 | 3 | 3 | 3 | 3 | **3** |
| duckling # 3 | 3 | 3 | 3 | 3 | 3 | 3 | 3 | 3 | 3 | 3 | **3** |
| duckling # 4 | 2 | 3 | 3 | 3 | 3 | 3 | 3 | 3 | 3 | 3 | **2.9** |
| duckling # 5 | 2 | 3 | 3 | 3 | 3 | 3 | 3 | 3 | 3 | 3 | **2.9** |
| duckling # 6 | 2 | 3 | 3 | 3 | 3 | 3 | 3 | 3 | 3 | 3 | **2.9** |
| duckling # 7 | 2 | 3 | 3 | 3 | 3 | 3 | 3 | 3 | 3 | 3 | **2.9** |
| duckling # 8 | 2 | 3 | 3 | 3 | 3 | 3 | 3 | 3 | 3 | 3 | **2.9** |
| duckling # 9 | 2 | 3 | 3 | 3 | 3 | 3 | 3 | 3 | 3 | 3 | **2.9** |
| duckling # 10 | 1 | 3 | 3 | 3 | 3 | 3 | 3 | 3 | 3 | 3 | **2.8** |

| #5 | day 1 | day 2 | day 3 | day 4 | day 5 | day 6 | day 7 | day 8 | day 9 | day 10 | evaluation |
|---|---|---|---|---|---|---|---|---|---|---|---|
| duckling # 1 | 0 | 0 | 2 | 3 | 3 | 3 | 3 | 3 | 3 | 3 | **2.3** |
| duckling # 2 | 0 | 0 | 0 | 2 | 3 | 3 | 3 | 3 | 3 | 3 | **2** |
| duckling # 3 | 0 | 0 | 0 | 2 | 3 | 3 | 3 | 3 | 3 | 3 | **2** |
| duckling # 4 | 0 | 0 | 0 | 2 | 3 | 3 | 3 | 3 | 3 | 3 | **2** |
| duckling # 5 | 0 | 0 | 0 | 1 | 3 | 3 | 3 | 3 | 3 | 3 | **1.9** |
| duckling # 6 | 0 | 0 | 0 | 1 | 3 | 3 | 3 | 3 | 3 | 3 | **1.9** |
| duckling # 7 | 0 | 0 | 0 | 0 | 2 | 3 | 3 | 3 | 3 | 3 | **1.7** |
| duckling # 8 | 0 | 0 | 0 | 0 | 2 | 3 | 3 | 3 | 3 | 3 | **1.7** |
| duckling # 9 | 0 | 0 | 0 | 0 | 1 | 1 | 0 | 2 | 2 | 2 | **0.8** |
| duckling # 10 | 0 | 0 | 0 | 0 | 1 | 1 | 0 | 0 | 0 | 0 | **0.2** |
| duckling # 11 [S] | x | 0 | 0 | 2 | 3 | 3 | 3 | 3 | 3 | 3 | **2** |

| #6 | day 1 | day 2 | day 3 | day 4 | day 5 | day 6 | day 7 | day 8 | day 9 | day 10 | evaluation |
|---|---|---|---|---|---|---|---|---|---|---|---|
| duckling # 1 | 3 | 3 | 3 | 3 | 3 | 3 | 3 | 3 | 3 | 3 | **3** |
| duckling # 2 | 3 | 3 | 3 | 3 | 3 | 3 | 3 | 3 | 3 | 3 | **3** |
| duckling # 3 | 2 | 3 | 3 | 3 | 3 | 3 | 3 | 3 | 3 | 3 | **2.9** |
| duckling # 4 | 2 | 3 | 3 | 3 | 3 | 3 | 3 | 3 | 3 | 3 | **2.9** |
| duckling # 5 | 0 | 0 | 0 | 2 | 3 | 3 | 3 | 3 | 3 | 3 | **2** |
| duckling # 6 | 0 | 0 | 0 | 0 | 1 | 1 | 0 | 0 | 1 | 2 | **0.5** |
| duckling # 7 | 0 | 0 | 0 | 0 | 1 | 0 | 0 | 0 | 1 | 1 | **0.3** |
| duckling # 8 | 0 | 0 | 0 | 0 | 1 | 0 | 0 | 0 | 0 | 1 | **0.2** |
| duckling # 9 | 0 | 0 | 0 | 0 | 1 | 0 | 0 | 0 | 0 | 1 | **0.2** |
| duckling # 10 | 0 | 0 | 0 | 0 | 1 | 0 | 0 | 0 | 0 | 1 | **0.2** |
| duckling # 11 [S] | x | 0 | 0 | 0 | 0 | 0 | 0 | 0 | 0 | 1 | **0.1** |

| #9 | day 1 | day 2 | day 3 | day 4 | day 5 | day 6 | day 7 | day 8 | day 9 | day 10 | evaluation |
|---|---|---|---|---|---|---|---|---|---|---|---|
| duckling # 1 | 0 | 0 | 1 | 3 | 3 | 3 | 3 | 3 | 3 | 3 | **2.2** |
| duckling # 2 | 0 | 0 | 0 | 2 | 3 | 3 | 3 | 3 | 3 | 3 | **2** |
| duckling # 3 | 0 | 0 | 0 | 1 | 2 | 3 | 3 | 3 | 3 | 3 | **1.8** |
| duckling # 4 | 0 | 0 | 0 | 1 | 2 | 3 | 3 | 3 | 3 | 3 | **1.8** |
| duckling # 5 | 0 | 0 | 0 | 0 | 2 | 3 | 3 | 3 | 3 | 3 | **1.7** |
| duckling # 6 | 0 | 0 | 0 | 0 | 1 | 1 | 2 | 2 | 2 | 2 | **1** |
| duckling # 7 | 0 | 0 | 0 | 0 | 1 | 1 | 1 | 2 | 1 | 0 | **0.6** |
| duckling # 8 | 0 | 0 | 0 | 0 | 1 | 1 | 1 | 1 | 1 | 0 | **0.5** |
| duckling # 9 | 0 | 0 | 0 | 0 | 1 | 1 | 1 | 1 | 1 | 0 | **0.5** |
| duckling # 10 | 0 | 0 | 0 | 0 | 1 | 1 | 1 | 1 | 1 | 0 | **0.5** |
| duckling # 11 [S] | x | 0 | 0 | 0 | 0 | 1 | 0 | 0 | 0 | 0 | **0.1** |

| #8 | day 1 | day 2 | day 3 | day 4 | day 5 | day 6 | day 7 | day 8 | day 9 | day 10 | evaluation |
|---|---|---|---|---|---|---|---|---|---|---|---|
| duckling # 1 | 1 | 1 | 1 | 2 | 3 | 3 | 3 | 3 | 3 | 3 | **2.3** |
| duckling # 2 | 0 | 0 | 1 | 2 | 3 | 3 | 3 | 3 | 3 | 3 | **2.1** |
| duckling # 3 | 0 | 0 | 1 | 1 | 3 | 3 | 3 | 3 | 3 | 3 | **2** |
| duckling # 4 | 0 | 0 | 0 | 1 | 2 | 3 | 3 | 3 | 3 | 3 | **1.8** |
| duckling # 5 | 0 | 0 | 0 | 0 | 1 | 2 | 2 | 1 | 1 | 0 | **0.7** |
| duckling # 6 | 0 | 0 | 0 | 0 | 1 | 2 | 2 | 1 | 0 | 0 | **0.6** |
| duckling # 7 | 0 | 0 | 0 | 0 | 1 | 2 | 2 | 1 | 0 | 0 | **0.6** |
| duckling # 8 | 0 | 0 | 0 | 0 | 1 | 1 | 2 | 1 | 0 | 0 | **0.5** |
| duckling # 9 | 0 | 0 | 0 | 0 | 1 | 1 | 1 | 1 | 0 | 0 | **0.4** |
| duckling # 10 | X | X | X | X | X | X | X | X | X | X | **X** |

| #2 | day 1 | day 2 | day 3 | day 4 | day 5 | day 6 | day 7 | day 8 | day 9 | day 10 | evaluation |
|---|---|---|---|---|---|---|---|---|---|---|---|
| duckling # 1 | 3 | 3 | 3 | 3 | 3 | 3 | 3 | 3 | 3 | 3 | **3** |
| duckling # 2 | 3 | 3 | 3 | 3 | 3 | 3 | 3 | 3 | 3 | 3 | **3** |
| duckling # 3 | 3 | 3 | 3 | 3 | 3 | 3 | 3 | 3 | 3 | 3 | **3** |
| duckling # 4 | 1 | 3 | 3 | 3 | 3 | 3 | 3 | 3 | 3 | 3 | **2.8** |
| duckling # 5 | 1 | 3 | 3 | 3 | 3 | 3 | 3 | 3 | 3 | 3 | **2.8** |
| duckling # 6 | 0 | 3 | 3 | 3 | 3 | 3 | 3 | 3 | 3 | 3 | **2.7** |
| duckling # 7 | 0 | 0 | 3 | 3 | 3 | 3 | 3 | 3 | 3 | 3 | **2.4** |
| duckling # 8 | 0 | 0 | 3 | 3 | 3 | 3 | 3 | 3 | 3 | 3 | **2.4** |
| duckling # 9 | 0 | 0 | 3 | 3 | 3 | 3 | 3 | 3 | 3 | 3 | **2.4** |
| duckling # 10 | 0 | 0 | 3 | 3 | 3 | 3 | 3 | 3 | 3 | 3 | **2.4** |

| #1 | day 1 | day 2 | day 3 | day 4 | day 5 | day 6 | day 7 | day 8 | day 9 | day 10 | evaluation |
|---|---|---|---|---|---|---|---|---|---|---|---|
| duckling # 1 | 3 | 3 | 3 | 3 | 3 | 3 | 3 | 3 | 3 | 3 | **3** |
| duckling # 2 | 3 | 3 | 3 | 3 | 3 | 3 | 3 | 3 | 3 | 3 | **3** |
| duckling # 3 | 1 | 3 | 3 | 3 | 3 | 3 | 3 | 3 | 3 | 3 | **2.8** |
| duckling # 4 | 1 | 3 | 3 | 3 | 3 | 3 | 3 | 3 | 3 | 3 | **2.8** |
| duckling # 5 | 1 | 1 | 3 | 3 | 3 | 3 | 3 | 3 | 3 | 3 | **2.6** |
| duckling # 6 | 1 | 1 | 2 | 3 | 3 | 3 | 3 | 3 | 3 | 3 | **2.5** |
| duckling # 7 | 1 | 1 | 2 | 3 | 3 | 3 | 3 | 3 | 3 | 3 | **2.5** |
| duckling # 8 | 1 | 1 | 0 | 2 | 3 | 3 | 3 | 3 | 3 | 3 | **2.2** |
| duckling # 9 | 1 | 1 | 0 | 0 | 2 | 3 | 3 | 3 | 3 | 3 | **1.9** |
| duckling # 10 | 1 | 1 | 0 | 0 | 2 | 3 | 3 | 3 | 3 | 3 | **1.9** |
| duckling # 11 [S] | - | 0 | 0 | 2 | 3 | 3 | 3 | 3 | 3 | 3 | **2** |

**Supplementary Table 7. Analysis of Protein sequences of relevant genotype segments**

| | PB2 | | PB1 | | PA | | PA-X | | HA | | NP | | NA | | M | | M2 | | NS | | NS-NEG | |
|---|---|---|---|---|---|---|---|---|---|---|---|---|---|---|---|---|---|---|---|---|---|---|
| aa | 760 | | 757 | | 706 | | 253 | | 567 | | 499 | | 470 | | 252 | | 97 | | 224 | | | |
| **Genotype** | total | unique | total | unique | total | unique | total | unique | **total** | **unique** | total | unique | **total** | **unique** | **total** | **unique** | **total** | **unique** | **total** | unique | **total** | unique |
| #3 | | | | | * | | * | | | | | | * | | | | | | | | * | |
| #4 (3) | 7 | 2 | 4 | 1 | 10 | - | 4 | 1 | 3 | 1 | 4 | - | 4 | - | - | - | - | - | 3 | - | - | - |
| #4 (1) | 8 | 2 | 3 | - | 11 | 1 | 4 | - | 2 | - | 6 | 1 | 6 | 1 | 1 | 1 | 1 | - | 4 | - | 1 | - |
| #4 (2) | 7 | | 3 | - | 11 | 1 | 5 | - | 2 | 1 | 5 | - | 6 | 1 | - | - | - | - | 4 | - | 1 | - |
| #7 | 5 | | 9 | 4 | 2 | - | 3 | 1 | 2 | - | 10 | - | 7 | 2 | 1 | 1 | 1 | - | 4 | - | - | - |
| #5 | 5 | 2 | 2 | 2 | 15 | 8 | 5 | 4 | 3 | - | 2 | 1 | 11 | 5 | 2 | 2 | 2 | 2 | 6 | - | 1 | - |
| #6 | 3 | | 6 | 3 | 14 | 4 | 5 | 2 | | 1 | 2 | - | 5 | 1 | - | - | - | - | 4 | - | - | - |
| #8 | 8 | 3 | 7 | 2 | 3 | 1 | 3 | - | 3 | 1 | 10 | - | 5 | - | - | - | - | - | 4 | - | - | - |
| #9 | 4 | 1 | 5 | 3 | 12 | 2 | 3 | - | 3 | 3 | 3 | 1 | 5 | 1 | - | - | - | - | 68 | | 21 | 15 |
| #1 | 15 | 10 | 15 | 4 | 16 | - | 7 | - | 4 | 1 | 6 | - | | | 1 | 1 | 1 | 1 | | | 10 | 1 |
| #2 | 9 | 4 | 13 | - | 19 | 2 | 9 | 2 | | 1 | 5 | 1 | | | - | - | - | - | | | 9 | - |

PB2: amino acids (1-760)

| Genotype | 61 | 63 | 76 | 79 | # | # | # | # | # | # | # | # | # | # | # | # | # | # | # | # | # | # | # | # | # | # | # | # | # | # | # | # | # | # | # |
|---|---|---|---|---|---|---|---|---|---|---|---|---|---|---|---|---|---|---|---|---|---|---|---|---|---|---|---|---|---|---|---|---|---|---|---|
| #3 | K | I | T | G | V | R | K | E | H | I | F | V | I | V | R | A | V | D | I | N | I | K | Q | Q | T | T | P | K | E | A | T | T | D | D | S |
| #4 (1) | . | . | . | S | . | Q | . | . | . | . | . | . | . | . | . | . | . | . | V | S | . | R | . | . | I | . | T | . | . | . | . | . | . | . | N |
| #4 (2) | . | . | . | S | . | | . | G | . | . | . | . | . | . | . | . | . | . | V | | . | R | . | . | I | . | T | . | . | . | . | . | . | . | N |
| #4 (3) | . | . | . | S | . | . | . | G | . | . | . | . | . | . | . | . | . | E | V | . | . | R | . | . | . | . | . | R | . | . | . | . | . | . | N |
| #5 | R | . | . | S | . | . | . | . | . | . | . | . | . | . | . | . | . | . | . | . | . | R | . | . | . | . | . | . | . | . | . | A | . | . | N |
| #9 | . | . | . | S | . | . | . | . | . | . | L | . | . | . | . | . | . | . | . | . | . | R | . | . | . | . | . | . | . | . | . | . | . | . | N |
| #6 | . | . | . | S | . | . | . | . | . | . | . | . | . | . | . | . | . | . | . | . | . | R | . | . | . | . | . | . | . | . | . | . | . | . | N |
| #7 | . | . | . | S | . | . | . | . | . | . | . | . | . | . | . | . | . | . | . | . | . | R | . | . | . | . | . | . | . | . | I | . | . | G | N |
| #8 | . | V | . | S | . | . | . | . | . | . | . | . | . | . | K | . | . | . | . | . | . | R | K | . | . | . | . | . | . | . | I | . | . | G | N |
| #2 | . | . | I | S | . | . | . | . | . | V | . | . | . | . | . | T | A | . | . | . | . | R | . | . | . | N | . | . | . | . | . | . | Y | . | N |
| #1 | . | . | . | S | I | . | R | . | Q | V | . | I | V | I | . | . | . | . | . | . | V | R | . | H | . | . | . | . | K | T | . | . | Y | . | N |

PB1: amino acids (1-757)

| Genotype | 16 | 20 | 62 | 69 | # | # | # | # | # | # | # | # | # | # | # | # | # | # | # | # | # | # | # | # | # | # | # | # | # | # | # | # | # |
|---|---|---|---|---|---|---|---|---|---|---|---|---|---|---|---|---|---|---|---|---|---|---|---|---|---|---|---|---|---|---|---|---|---|
| #3 | D | T | G | I | E | T | V | V | A | T | K | D | E | I | I | N | K | M | I | M | A | S | K | I | I | L | V | E | G | R | S | V | E |
| #7 | | | | V | | | | | | | | | | | | T | | | | | | | | | | | | | | | | | |
| #5 | N | | | | G | | | | | A | | | | | | | R | | | I | | | R | | V | | | | | K | | | G |
| #8 | N | | | | | | | | | A | | | G | | | | | | | | | | R | | V | I | | | | K | | | |
| #4:1 | | | | | | | | | | A | | | | | | | | | | | | | | | | | | | | K | G | | |
| #4:2 | | | | | | | | I | | A | | | | | | | | | | | | | | | | | | | | K | G | | |
| #4:3 | | | | | | | | | | A | | | | | | | | | | | | | | | | | | | | K | G | | |
| #6 | | N | | | | | | | | A | | | | | | | | V | | | | | | | V | | | | | K | | I | |
| #9 | | | R | | | | | | | A | | | | | | | | | V | | | | | | V | | | | D | | | | |
| #2 | N | | | | | | I | | S | A | R | E | | | | | | | | | T | P | R | V | V | | I | | | K | | | |
| #1 | N | | | | | A | I | | S | A | R | E | | V | L | | | | | | T | P | | | V | | I | G | | K | | | |

PB1-F2

| Genotype | 4 | 6 | 7 | 8 | # | # | # | # | # | # | # | # | # | # | # | # | # | # | # | # | # | # | # | # | # | # | # | # | # |
|---|---|---|---|---|---|---|---|---|---|---|---|---|---|---|---|---|---|---|---|---|---|---|---|---|---|---|---|---|---|
| #8 | E | D | T | P | Q | S | R | K | R | P | D | Q | V | D | M | K | A | L | F | S | T | Q | G | K | L | R | S | F | E |
| #7 | G | | | Q | | | Q | R | K | L | G | R | A | | | | | | S | | | | | | | | | | |
| #3 | G | | | Q | | | Q | R | K | L | G | R | A | | | | | | S | | | | | | | | | | |
| #4:1 | G | | | Q | | | Q | R | K | L | G | R | A | | | | | | S | | I | | | | | | | | |
| #4:2 | G | | | Q | R | | Q | R | K | L | G | R | A | | | | | | S | | I | | | | | | | | |
| #4:3 | G | | | Q | R | | Q | R | K | L | G | R | A | | | | | | S | | I | | | | | | | | |
| #5 | G | | | Q | | | | R | K | L | G | R | A | A | | R | | | S | | | | | E | | | | | |
| #6 | G | | | Q | | | Q | R | K | L | G | R | | | | | | | S | | | | | | | | | | |
| #9 | G | | | Q | | | Q | R | K | L | G | R | A | | | | | | S | | | | | | | | | S | |
| | | | | | | | | | | | | | | | d* | | d* | $i^{\#}$ | | $v^{\#}$ | $c^{\#}$ | $c^{\#}$ | $c^{\#}$ | $i^{\#}$ | $i^{\#}$ | $i^{\#}$ | $i^{\#}$ | | d* |

*Marjuki et al., 2010 # Kamal et al., 2017

i: inflamatory; c: cytotoxic; ref: Kamal et al. , 2017 reviewed; d: duck virulence; v: mouse virulence

PA: amino acids (1-706)

| Genotype | 7 | 16 | 50 | # | # | # | # | # | # | # | # | # | # | # | # | # | # |
|---|---|---|---|---|---|---|---|---|---|---|---|---|---|---|---|---|---|
| #3 | E | D | M | E | T | N | C | K | N | L | L | D | P | T | R | E | I |
| #7 | . | . | T | . | . | . | . | . | . | . | . | . | . | . | G | . | . |
| #5 | G | Y | . | D | I | S | Y | . | K | V | I | N | S | S | G | A | L |
| #8 | . | . | T | . | . | . | . | E | . | . | . | . | . | . | G | . | . |

PA-X: amino acids (1-253)

| Genotype | 18 | 27 | 61 | # | # | # | # | # |
|---|---|---|---|---|---|---|---|---|
| #3 | E | D | M | E | R | P | R | T |
| #7 | . | . | T | . | K | . | L | . |
| #5 | G | Y | . | D | . | . | . | A |
| #8 | . | . | T | . | K | Q | . | . |

HA: amino acids (1-567)

| Genotype | 10 | # | # | # | # | # | # | # | # |
|---|---|---|---|---|---|---|---|---|---|
| #3 | T | P | V | I | I | I | S | I | S |
| #7 | . | S | L | . | . | . | . | . | . |
| #5 | I | . | . | . | K | M | . | M | . |
| #8 | . | S | L | . | . | . | F | . | F |

NP: amino acids (1-499)

| Genotype | 41 | 44 | 51 | 52 | 85 | 86 | # | # | # | # | # |
|---|---|---|---|---|---|---|---|---|---|---|---|
| #3 | I | C | E | H | A | G | V | A | I | S | R |
| #7 | V | G | D | Y | K | E | M | . | V | L | K |
| #5 | . | . | D | . | . | . | . | V | . | . | . |
| #8 | V | G | D | Y | K | E | M | . | V | L | K |

NA: amino acids (1-470)

| Genotype | 6 | 8 | 10 | 17 | 46 | # | # | # | # | # | # | # | # | # |
|---|---|---|---|---|---|---|---|---|---|---|---|---|---|---|
| #3 | R | I | T | T | S | P | I | I | D | E | ? | V | A | T |
| #7 | . | . | . | I | P | . | S | M | N | . | K | I | . | . |
| #5 | K | T | I | I | P | S | . | . | N | D | K | . | E | S |
| #8 | . | . | . | I | P | . | . | M | N | . | K | . | . | . |

M1: amino acids (1-252)

| Genotype | # | # | # |
|---|---|---|---|
| #3 | E | S | D |
| #7 | G | . | . |
| #5 | . | N | N |
| #8 | . | . | . |

M2: amino acids (1-98)

| Genotype | # | # | # |
|---|---|---|---|
| #3 | E | S | D |
| #7 | G | . | . |
| #5 | . | N | N |
| #8 | . | . | . |

NS Neg: amino acids (1-122)

| Genotype | # |
|---|---|
| #3 | G |
| #7 | . |
| #5 | E |
| #8 | . |

NS: amino acids (1-224)

| Genotype | 79 | 95 | # | # | # | # | # | # |
|---|---|---|---|---|---|---|---|---|
| #3 | I | L | R | N | I | Y | V | R |
| #7 | M | . | K | . | . | N | I | . |
| #5 | M | . | K | T | L | D | . | G |
| #8 | M | P | K | . | . | N | . | . |

**Supplementary Table 8. Onset of disease per animal between genotype #4:3 and #8 between i.m. and o.n. inoculation**

| | | animal identification number | onset of disease | time to onset of disease [d] | death after onset of disease [d] | death by trial end | euthanized/ found dead | number of diseased animals (morbidity) | | | | |
|---|---|---|---|---|---|---|---|---|---|---|---|---|
| o.n. inoculation | genotype #4 | #42 | peracutely deceased | 2 | 3 | | peracutely deceased | | 12 | 12 | Morbidity | 100.0 |
| | | #43 | CNS disorder | 2 | 3 | | euthanized | | | 12 | Mortality | 100.0 |
| | | #44 | CNS disorder | 2 | 3 | | euthanized | | | 12 | Lethality | 100.0 |
| | | #45 | peracutely deceased | 3 | 3 | | peracutely deceased | | | | | |
| | | #46 | CNS disorder | 3 | 3 | | euthanized | | | | | |
| | | #47 | CNS disorder | 3 | 3 | | euthanized | | | | | |
| | | #48 | CNS disorder | 3 | 3 | | euthanized | (12/12) | | | | |
| | | #50 | peracutely deceased | 3 | 3 | | peracutely deceased | | | | | |
| | | #99 | peracutely deceased | 3 | 3 | | peracutely deceased | | | | | |
| | | #49 | CNS disorder | 4 | 4 | | euthanized | | | | | |
| | | arithmetic mean | | 2.8 | 3.1 | | | | | | | |
| | | #549 [S] | peracutely deceased | 5 | 5 | | peracutely deceased | | | | | |
| | | #550 [S] | peracutely deceased | 5 | 5 | | peracutely deceased | | | | | |
| i.m. inoculation | genotype #4 | #1 | peracutely deceased | 1 | 1 | | peracutely deceased | | 10 | 10 | Morbidity | 100.0 |
| | | #2 | peracutely deceased | 1 | 1 | | peracutely deceased | | | 10 | Mortality | 100.0 |
| | | #3 | peracutely deceased | 1 | 1 | | peracutely deceased | | | 10 | Lethality | 100.0 |
| | | #4 | peracutely deceased | 1 | 1 | | peracutely deceased | | | | | |
| | | #5 | peracutely deceased | 1 | 1 | | peracutely deceased | | | | | |
| | | #6 | peracutely deceased | 1 | 1 | | peracutely deceased | (10/10) | | | | |
| | | #7 | peracutely deceased | 1 | 1 | | peracutely deceased | | | | | |
| | | #8 | peracutely deceased | 1 | 1 | | peracutely deceased | | | | | |
| | | #9 | peracutely deceased | 1 | 1 | | peracutely deceased | | | | | |
| | | #10 | CNS disorder | 1 | 1 | | euthanized | | | | | |
| | | x | | | | | | | | | | |
| | | x | | | | | | | | | | |
| o.n. inoculation | genotype #8 | #392 | CNS disorder | 5 | 5 | | euthanized | | 12 | 7 | Morbidity | 58.3 |
| | | #395 | CNS disorder | 5 | 5 | | euthanized | | | 5 | Mortality | 41.7 |
| | | #399 | peracutely deceased | 6 | 6 | | peracutely deceased | | | 5 | Lethality | 71.4 |
| | | #400 | CNS disorder | 6 | 6 | | euthanized | | | | | |
| | | #390 | | | | 21 | | | | | | |
| | | #391 | | | | 21 | | | | | | |
| | | #393 | | | | 21 | | (5/12) | | | | |
| | | #396 | | | | 21 | | | | | | |
| | | #397 | | | | 21 | | | | | | |
| | | #398 | | | | 21 | | | | | | |
| | | arithmetic mean | | 5.5 | 5.5 | | | | | | | |
| | | #66 [S] | CNS disorder | 6 | 6 | | euthanized | | | | | |
| | | #64 [S] | | | | 10 | euthanized | | | | | |
| i.m. inoculation | genotype #8 | #1 | CNS disorder | 1 | 4 | | euthanized | | 9 | 9 | Morbidity | 100.0 |
| | | #2 | CNS disorder | 3 | 4 | | euthanized | | | 4 | Mortality | 44.4 |
| | | #3 | peracutely deceased | 3 | 4 | | peracutely deceased | | | 4 | Lethality | 44.4 |
| | | #4 | CNS disorder | 5 | 5 | | euthanized | | | | | |
| | | #5 | reduced general condition | 5 | | 10 | euthanized | | | | | |
| | | #6 | reduced general condition | 5 | | 10 | euthanized | | | | | |
| | | #7 | reduced general condition | 5 | | 10 | euthanized | (4/9) | | | | |
| | | #8 | reduced general condition | 5 | | 10 | euthanized | | | | | |
| | | #9 | reduced general condition | 5 | | 10 | euthanized | | | | | |
| | | arithmetic mean | | 4.1 | 4.3 | | | | | | | |
| | | x | | | | | | | | | | |
| | | x | | | | | | | | | | |
| | | x | | | | | | | | | | |

**Supplementary Table 9: Virus antigen detection following oculo-nasal (genotype #4:3 and #8) or intramuscular infection (genotype #8) of pekin ducklings. The number given indicates the number of positive animals per group (n=3/group), the color code refers to the maximum score for distribution of antigen within this group: green = none; yellow = focal / oligofocal; orange = multifocal; red = coalescing; magenta = diffuse**

| | | route | on | on | on | im |
|---|---|---|---|---|---|---|
| | | virus | A/chicken/DE-NI/AI 4286/2022 | A/red knot/DE-SH/AI 616/2022 | A/red knot/DE-SH/AI 616/2022 | A/red knot/DE-SH/AI 616/2022 |
| | | genotype | #4 | #8 | #8 | #8 |
| | | dpi | 3 dpi | 5 dpi | 21 dpi, survivors | 10 dpi, survivors |
| | **Tissue** | **cell type** | | | | |
| **Central nervous system** | forebrain | neuron | 3 | 3 | 0 | 2 |
| | | glial cell | 3 | 3 | 0 | 1 |
| | | meninges | 0 | 1 | 0 | 0 |
| | | epithelium, ependyma | 2 | 1 | 0 | 0 |
| | cerebellum | neuron | 2 | 1 | 0 | 0 |
| | | glial cell | 2 | 1 | 0 | 0 |
| | brain stem | neuron | 3 | 2 | 0 | 1 |
| | | glial cell | 1 | 1 | 0 | 0 |
| | spinal cord | neuron | 3 | 0 | 0 | 0 |
| | | glial cell | 2 | 0 | 0 | 0 |
| | | neuron, dorsal root ganglia | 3 | 0 | 0 | 0 |
| | | epithelium, ependyma, central canal | 3 | 0 | 0 | 0 |
| **Conchae** | vestibulum | epithelium, squamous | 3 | 0 | 0 | 0 |
| | conchae, respiratory | epithelium, ciliated | 2 | 0 | 0 | 0 |
| | | epithelium, glands, lining mucosa | 2 | 0 | 0 | 0 |
| | | epithelium, glands, submucosa | 3 | 2 | 0 | 0 |
| | | epithelium, sinus / duct | 2 | 1 | 0 | 0 |
| | conchae, olfactory | epithelium, olfactory | 2 | 0 | 0 | 0 |
| | | epithelium, bowmans gland | 2 | 0 | 0 | 0 |
| **Lung** | | epithelium, bronchi, goblet | 0 | 0 | 0 | 0 |
| | | epithelium, bronchi, ciliated | 1 | 0 | 0 | 0 |
| | | endothelium | 1 | 0 | 0 | 0 |
| | | epithelium, parabronchi, atria , capillary | 3 | 0 | 0 | 0 |
| | | epithelium, airsac/ostium | 1 | 2 | 0 | 0 |
| **Kidney** | | epithelium, tubular | 2 | 0 | 0 | 0 |
| **Heart** | | cardiomyocyte | 2 | 1 | 0 | 0 |

**Legend:**

| **Score** | **Definition, incidence** | **Descriptive term** |
|---|---|---|
| 0 | no antigen | |
| 1 | focal / oligofocal (<5% | minimal |
| 2 | multifocal (6%–40%) | mild |
| 3 | coalescing (41%–80% | moderate |
| 4 | diffuse >80% | severe |

**note (not for publication)**
**cerebrum = telencephalon, diencephalon contains the thalamus and hypothalamus**
**forebrain = cerebrum, thalamus, hypothalamus, pituitary gland, limbic system, and the olfactory bulb**

**Supplementary Table 10. Clinical outcome per animal and day of IMPI for all groups**

| | animal | day 1 | day 2 | day 3 | day 4 | day 5 | day 6 | day 7 | day 8 | day 9 | day 10 | result IMPI per animal and day | cause of death |
|---|---|---|---|---|---|---|---|---|---|---|---|---|---|
| **genotype #1** | #1 | 3 | 3 | 3 | 3 | 3 | 3 | 3 | 3 | 3 | 3 | **3** | peracutely deceased |
| | #2 | 3 | 3 | 3 | 3 | 3 | 3 | 3 | 3 | 3 | 3 | **3** | peracutely deceased |
| | #3 | 1 | 3 | 3 | 3 | 3 | 3 | 3 | 3 | 3 | 3 | **2.8** | peracutely deceased |
| | #4 | 1 | 3 | 3 | 3 | 3 | 3 | 3 | 3 | 3 | 3 | **2.8** | peracutely deceased |
| | #5 | 1 | 1 | 3 | 3 | 3 | 3 | 3 | 3 | 3 | 3 | **2.6** | peracutely deceased |
| | #6 | 1 | 1 | 2 | 3 | 3 | 3 | 3 | 3 | 3 | 3 | **2.5** | euthanized |
| | #7 | 1 | 1 | 2 | 3 | 3 | 3 | 3 | 3 | 3 | 3 | **2.5** | euthanized |
| | #8 | 1 | 1 | 0 | 2 | 3 | 3 | 3 | 3 | 3 | 3 | **2.2** | euthanized |
| | #9 | 1 | 1 | 0 | 0 | 2 | 3 | 3 | 3 | 3 | 3 | **1.9** | euthanized |
| | #10 | 1 | 1 | 0 | 0 | 2 | 3 | 3 | 3 | 3 | 3 | **1.9** | euthanized |
| | # 11 [S] | X | 0 | 0 | 2 | 3 | 3 | 3 | 3 | 3 | 3 | **2** | euthanized |
| **genotype #2** | #1 | 3 | 3 | 3 | 3 | 3 | 3 | 3 | 3 | 3 | 3 | **3** | peracutely deceased |
| | #2 | 3 | 3 | 3 | 3 | 3 | 3 | 3 | 3 | 3 | 3 | **3** | peracutely deceased |
| | #3 | 3 | 3 | 3 | 3 | 3 | 3 | 3 | 3 | 3 | 3 | **3** | peracutely deceased |
| | #4 | 1 | 3 | 3 | 3 | 3 | 3 | 3 | 3 | 3 | 3 | **2.8** | peracutely deceased |
| | #5 | 1 | 3 | 3 | 3 | 3 | 3 | 3 | 3 | 3 | 3 | **2.8** | peracutely deceased |
| | #6 | 0 | 3 | 3 | 3 | 3 | 3 | 3 | 3 | 3 | 3 | **2.7** | peracutely deceased |
| | #7 | 0 | 0 | 3 | 3 | 3 | 3 | 3 | 3 | 3 | 3 | **2.4** | peracutely deceased |
| | #8 | 0 | 0 | 3 | 3 | 3 | 3 | 3 | 3 | 3 | 3 | **2.4** | peracutely deceased |
| | #9 | 0 | 0 | 3 | 3 | 3 | 3 | 3 | 3 | 3 | 3 | **2.4** | peracutely deceased |
| | #10 | 0 | 0 | 3 | 3 | 3 | 3 | 3 | 3 | 3 | 3 | **2.4** | peracutely deceased |
| **genotype #3** | #1 | 3 | 3 | 3 | 3 | 3 | 3 | 3 | 3 | 3 | 3 | **3** | peracutely deceased |
| | #2 | 3 | 3 | 3 | 3 | 3 | 3 | 3 | 3 | 3 | 3 | **3** | peracutely deceased |
| | #3 | 3 | 3 | 3 | 3 | 3 | 3 | 3 | 3 | 3 | 3 | **3** | peracutely deceased |
| | #4 | 2 | 3 | 3 | 3 | 3 | 3 | 3 | 3 | 3 | 3 | **2.9** | euthanized |
| | #5 | 2 | 3 | 3 | 3 | 3 | 3 | 3 | 3 | 3 | 3 | **2.9** | euthanized |
| | #6 | 2 | 3 | 3 | 3 | 3 | 3 | 3 | 3 | 3 | 3 | **2.9** | euthanized |
| | #7 | 2 | 3 | 3 | 3 | 3 | 3 | 3 | 3 | 3 | 3 | **2.9** | euthanized |
| | #8 | 2 | 3 | 3 | 3 | 3 | 3 | 3 | 3 | 3 | 3 | **2.9** | euthanized |
| | #9 | 2 | 3 | 3 | 3 | 3 | 3 | 3 | 3 | 3 | 3 | **2.9** | euthanized |
| | #10 | 1 | 3 | 3 | 3 | 3 | 3 | 3 | 3 | 3 | 3 | **2.8** | euthanized |
| **genotype #4:1** | #1 | 3 | 3 | 3 | 3 | 3 | 3 | 3 | 3 | 3 | 3 | **3** | peracutely deceased |
| | #2 | 3 | 3 | 3 | 3 | 3 | 3 | 3 | 3 | 3 | 3 | **3** | peracutely deceased |
| | #3 | 3 | 3 | 3 | 3 | 3 | 3 | 3 | 3 | 3 | 3 | **3** | peracutely deceased |
| | #4 | 3 | 3 | 3 | 3 | 3 | 3 | 3 | 3 | 3 | 3 | **3** | peracutely deceased |
| | #5 | 3 | 3 | 3 | 3 | 3 | 3 | 3 | 3 | 3 | 3 | **3** | peracutely deceased |
| | #6 | 3 | 3 | 3 | 3 | 3 | 3 | 3 | 3 | 3 | 3 | **3** | peracutely deceased |
| | #7 | 3 | 3 | 3 | 3 | 3 | 3 | 3 | 3 | 3 | 3 | **3** | peracutely deceased |
| | #8 | 3 | 3 | 3 | 3 | 3 | 3 | 3 | 3 | 3 | 3 | **3** | peracutely deceased |
| | #9 | 3 | 3 | 3 | 3 | 3 | 3 | 3 | 3 | 3 | 3 | **3** | peracutely deceased |
| | #10 | 3 | 3 | 3 | 3 | 3 | 3 | 3 | 3 | 3 | 3 | **3** | peracutely deceased |
| **genotype #4:2** | #1 | 3 | 3 | 3 | 3 | 3 | 3 | 3 | 3 | 3 | 3 | **3** | peracutely deceased |
| | #2 | 3 | 3 | 3 | 3 | 3 | 3 | 3 | 3 | 3 | 3 | **3** | peracutely deceased |
| | #3 | 3 | 3 | 3 | 3 | 3 | 3 | 3 | 3 | 3 | 3 | **3** | peracutely deceased |
| | #4 | 3 | 3 | 3 | 3 | 3 | 3 | 3 | 3 | 3 | 3 | **3** | peracutely deceased |
| | #5 | 3 | 3 | 3 | 3 | 3 | 3 | 3 | 3 | 3 | 3 | **3** | peracutely deceased |
| | #6 | 2 | 3 | 3 | 3 | 3 | 3 | 3 | 3 | 3 | 3 | **2.9** | euthanized |
| | #7 | 2 | 3 | 3 | 3 | 3 | 3 | 3 | 3 | 3 | 3 | **2.9** | euthanized |
| | #8 | 2 | 3 | 3 | 3 | 3 | 3 | 3 | 3 | 3 | 3 | **2.9** | euthanized |
| | #9 | 2 | 3 | 3 | 3 | 3 | 3 | 3 | 3 | 3 | 3 | **2.9** | euthanized |
| | #10 | 2 | 3 | 3 | 3 | 3 | 3 | 3 | 3 | 3 | 3 | **2.9** | euthanized |
| **genotype #4:3** | #1 | 3 | 3 | 3 | 3 | 3 | 3 | 3 | 3 | 3 | 3 | **3** | peracutely deceased |
| | #2 | 3 | 3 | 3 | 3 | 3 | 3 | 3 | 3 | 3 | 3 | **3** | peracutely deceased |
| | #3 | 3 | 3 | 3 | 3 | 3 | 3 | 3 | 3 | 3 | 3 | **3** | peracutely deceased |
| | #4 | 3 | 3 | 3 | 3 | 3 | 3 | 3 | 3 | 3 | 3 | **3** | peracutely deceased |
| | #5 | 3 | 3 | 3 | 3 | 3 | 3 | 3 | 3 | 3 | 3 | **3** | peracutely deceased |
| | #6 | 3 | 3 | 3 | 3 | 3 | 3 | 3 | 3 | 3 | 3 | **3** | peracutely deceased |
| | #7 | 3 | 3 | 3 | 3 | 3 | 3 | 3 | 3 | 3 | 3 | **3** | peracutely deceased |
| | #8 | 3 | 3 | 3 | 3 | 3 | 3 | 3 | 3 | 3 | 3 | **3** | peracutely deceased |
| | #9 | 3 | 3 | 3 | 3 | 3 | 3 | 3 | 3 | 3 | 3 | **3** | peracutely deceased |
| | #10 | 2 | 3 | 3 | 3 | 3 | 3 | 3 | 3 | 3 | 3 | **2.9** | euthanized |
| **genotype #5** | #1 | 0 | 0 | 2 | 3 | 3 | 3 | 3 | 3 | 3 | 3 | **2.3** | euthanized |
| | #2 | 0 | 0 | 0 | 2 | 3 | 3 | 3 | 3 | 3 | 3 | **2** | euthanized |
| | #3 | 0 | 0 | 0 | 2 | 3 | 3 | 3 | 3 | 3 | 3 | **2** | euthanized |
| | #4 | 0 | 0 | 0 | 2 | 3 | 3 | 3 | 3 | 3 | 3 | **2** | euthanized |
| | #5 | 0 | 0 | 0 | 1 | 3 | 3 | 3 | 3 | 3 | 3 | **1.9** | peracutely deceased |
| | #6 | 0 | 0 | 0 | 1 | 3 | 3 | 3 | 3 | 3 | 3 | **1.9** | peracutely deceased |
| | #7 | 0 | 0 | 0 | 0 | 2 | 3 | 3 | 3 | 3 | 3 | **1.7** | euthanized |
| | #8 | 0 | 0 | 0 | 0 | 2 | 3 | 3 | 3 | 3 | 3 | **1.7** | euthanized |
| | #9 | 0 | 0 | 0 | 0 | 1 | 1 | 0 | 2 | 2 | 2 | **0.8** | euthanized |
| | #10 | 0 | 0 | 0 | 0 | 1 | 1 | 0 | 0 | 0 | 0 | **0.2** | euthanized |
| | # 11 [S] | x | 0 | 0 | 2 | 3 | 3 | 3 | 3 | 3 | 3 | **2** | euthanized |
| **genotype #6** | #1 | 3 | 3 | 3 | 3 | 3 | 3 | 3 | 3 | 3 | 3 | **3** | peracutely deceased |
| | #2 | 3 | 3 | 3 | 3 | 3 | 3 | 3 | 3 | 3 | 3 | **3** | peracutely deceased |
| | #3 | 2 | 3 | 3 | 3 | 3 | 3 | 3 | 3 | 3 | 3 | **2.9** | euthanized |
| | #4 | 2 | 3 | 3 | 3 | 3 | 3 | 3 | 3 | 3 | 3 | **2.9** | euthanized |
| | #5 | 0 | 0 | 0 | 2 | 3 | 3 | 3 | 3 | 3 | 3 | **2** | euthanized |
| | #6 | 0 | 0 | 0 | 0 | 1 | 1 | 0 | 0 | 1 | 2 | **0.5** | euthanized |
| | #7 | 0 | 0 | 0 | 0 | 1 | 0 | 0 | 0 | 1 | 1 | **0.3** | euthanized |
| | #8 | 0 | 0 | 0 | 0 | 1 | 0 | 0 | 0 | 0 | 1 | **0.2** | euthanized |
| | #9 | 0 | 0 | 0 | 0 | 1 | 0 | 0 | 0 | 0 | 1 | **0.2** | euthanized |
| | #10 | 0 | 0 | 0 | 0 | 1 | 0 | 0 | 0 | 0 | 1 | **0.2** | euthanized |
| | # 11 [S] | x | 0 | 0 | 0 | 0 | 0 | 0 | 0 | 0 | 1 | **0.1** | euthanized |
| **genotype #7** | #1 | 3 | 3 | 3 | 3 | 3 | 3 | 3 | 3 | 3 | 3 | **3** | peracutely deceased |
| | #2 | 3 | 3 | 3 | 3 | 3 | 3 | 3 | 3 | 3 | 3 | **3** | peracutely deceased |
| | #3 | 3 | 3 | 3 | 3 | 3 | 3 | 3 | 3 | 3 | 3 | **3** | peracutely deceased |
| | #4 | 3 | 3 | 3 | 3 | 3 | 3 | 3 | 3 | 3 | 3 | **3** | peracutely deceased |
| | #5 | 3 | 3 | 3 | 3 | 3 | 3 | 3 | 3 | 3 | 3 | **3** | peracutely deceased |
| | #6 | 3 | 3 | 3 | 3 | 3 | 3 | 3 | 3 | 3 | 3 | **3** | peracutely deceased |
| | #7 | 2 | 3 | 3 | 3 | 3 | 3 | 3 | 3 | 3 | 3 | **2.9** | euthanized |
| | #8 | 2 | 3 | 3 | 3 | 3 | 3 | 3 | 3 | 3 | 3 | **2.9** | euthanized |
| | #9 | 2 | 3 | 3 | 3 | 3 | 3 | 3 | 3 | 3 | 3 | **2.9** | euthanized |
| | #10 | 2 | 3 | 3 | 3 | 3 | 3 | 3 | 3 | 3 | 3 | **X** | euthanized |
| **genotype #8** | #1 | 1 | 1 | 1 | 2 | 3 | 3 | 3 | 3 | 3 | 3 | **2.3** | peracutely deceased |
| | #2 | 0 | 0 | 1 | 2 | 3 | 3 | 3 | 3 | 3 | 3 | **2.1** | euthanized |
| | #3 | 0 | 0 | 1 | 1 | 3 | 3 | 3 | 3 | 3 | 3 | **2** | euthanized |
| | #4 | 0 | 0 | 0 | 1 | 2 | 3 | 3 | 3 | 3 | 3 | **1.8** | euthanized |
| | #5 | 0 | 0 | 0 | 0 | 1 | 2 | 2 | 1 | 1 | 0 | **0.7** | euthanized |
| | #6 | 0 | 0 | 0 | 0 | 1 | 2 | 2 | 1 | 0 | 0 | **0.6** | euthanized |
| | #7 | 0 | 0 | 0 | 0 | 1 | 2 | 2 | 1 | 0 | 0 | **0.6** | euthanized |
| | #8 | 0 | 0 | 0 | 0 | 1 | 1 | 2 | 1 | 0 | 0 | **0.5** | euthanized |
| | #9 | 0 | 0 | 0 | 0 | 1 | 1 | 1 | 1 | 0 | 0 | **0.4** | euthanized |
| **genotype #9** | #1 | 0 | 0 | 1 | 3 | 3 | 3 | 3 | 3 | 3 | 3 | **2.2** | peracutely deceased |
| | #2 | 0 | 0 | 0 | 2 | 3 | 3 | 3 | 3 | 3 | 3 | **2** | euthanized |
| | #3 | 0 | 0 | 0 | 1 | 2 | 3 | 3 | 3 | 3 | 3 | **1.8** | euthanized |
| | #4 | 0 | 0 | 0 | 1 | 2 | 3 | 3 | 3 | 3 | 3 | **1.8** | euthanized |
| | #5 | 0 | 0 | 0 | 0 | 2 | 3 | 3 | 3 | 3 | 3 | **1.7** | euthanized |
| | #6 | 0 | 0 | 0 | 0 | 1 | 1 | 2 | 2 | 2 | 2 | **1** | euthanized |
| | #7 | 0 | 0 | 0 | 0 | 1 | 1 | 1 | 2 | 1 | 0 | **0.6** | euthanized |
| | #8 | 0 | 0 | 0 | 0 | 1 | 1 | 1 | 1 | 1 | 0 | **0.5** | euthanized |
| | #9 | 0 | 0 | 0 | 0 | 1 | 1 | 1 | 1 | 1 | 0 | **0.5** | euthanized |
| | #10 | 0 | 0 | 0 | 0 | 1 | 1 | 1 | 1 | 1 | 0 | **0.5** | euthanized |
| | # 11 [S] | x | 0 | 0 | 0 | 0 | 1 | 0 | 0 | 0 | 0 | **0.1** | euthanized |

clinical disease categorised as [0)] healthy, [1] sick, [2] severely sick and [3] dead; also cause of death is added

IMPI individual results are calculated from the mean value of all days/animal and give a value between 0-3.
The score is based on the established IVPI score for pathotyping of AIV strains.
A score > 1.2 indicates a highly pathogenic strain, while a score <1.2 indicates low pathogenicity.
